# Enaminones in a multicomponent synthesis of 4-aryldihydropyridines for potential applications in photoinduced intramolecular electron-transfer systems

**DOI:** 10.3762/bjoc.8.50

**Published:** 2012-03-26

**Authors:** Nouria A Al-Awadi, Maher R Ibrahim, Mohamed H Elnagdi, Elizabeth John, Yehia A Ibrahim

**Affiliations:** 1 Chemistry Department, Faculty of Science, Kuwait University, P.O. Box 5969, Safat 13060, Kuwait

**Keywords:** enaminones, 1,4-dihydropyridines, microwaves, multi-component reactions, photoluminescence

## Abstract

An efficient three component reaction with enaminones, primary amines and aldehydes resulted in easy access to 1,4-dihydropyridines with different substituents at the 1-, 3-, 4- and 5-positions. Microwaves improved the reaction yield, reducing also considerably the reaction time and the amount of solvent used. Chiral primary amines gave chiral 1-substituted-1,4-dihydropyridines. The 4-(1-naphthyl) and 4-(phenanthren-9-yl)dihydropyridine derivatives exhibited an interesting photoluminescence behavior, which suggests their potential application as suitable photoinduced intramolecular electron-transfer systems.

## Introduction

**Scheme 1:**
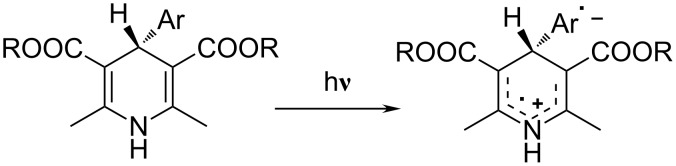
2,6-Dihydropyridine-3,5-dicarboxylates as useful organic dyads.

There is a lot of interest in supramolecular assemblies based on transition-metal ions, which have proved to be useful for a variety of light-induced applications, from molecular machines to systems that mimic chlorophyll photosynthesis [1–6]. Recently, 4-aryl-2,6-dihydropyridine-3,5-dicarboxylates have been investigated as useful organic dyads for the vectorial transport of energy or charge transfer [7–8] (Scheme 1). A few photochemical applications of dyads of this structure have been demonstrated including their use in photosensitive polymers [9–10], in biosensors or in the mapping of enzyme kinetics by means of the fluorescence similarity to NADH [11–13].

Moreover, there has been recent interest in the synthesis of dihydropyridine derivatives, due to their wide range of biological activity [14–15], by a one-pot three-component reaction with aliphatic/aromatic amines, ethyl propiolate and benzaldehyde [14], or by a cascade reaction of 1-phenylpropynone or ethyl propiolate with primary amines and aldehyde [15].

**Scheme 2:**
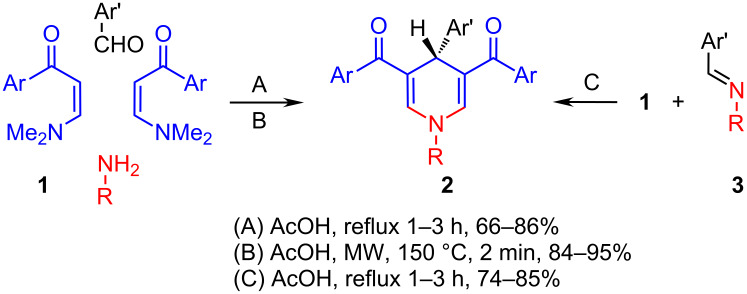
Synthesis of dihydropyridine derivatives from enaminones.

Enaminones are versatile starting materials for the synthesis of many classes of organic compounds and heterocyclic systems [16–17], and are prepared by various methods, for example, **1** is readily obtained in excellent yield by the condensation of different methylketones with dimethylformamide dimethylacetal (DMFDMA) [16–17]. In this work we investigated the potential utility of **1** in a three-component synthesis of dihydropyridines (DHP) (Scheme 2). This is expected to produce DHP with no substitution at the 2-position and different substituents at the 1-, 3-, 4- and 5-positions. This system contains the characteristic cyclic enaminone chromophore, which is expected to exhibit strong UV absorption with a maximum around 350 nm and extending to the border of the visible region. In the presence of an appropriate electron-acceptor substituent in position 4, the absorbed UV irradiation can cause intramolecular electron transfer, thus converting light into charge separation over a distance of ca. 6 Å. This expectation is based on the recent studies of DHPs containing the enaminocarboxylate chromophore with suitable substituents in the 4-position [7–8]. The DHP products reported in the present synthesis allow an easy method for a wide range of DHP derivatives having this expected characteristic of a photoinduced intramolecular electron-transfer system.

## Results and Discussion

In the present work we have investigated the synthesis of DHPs **2** from **1**, aromatic aldehydes, and ammonia or primary amines, in a three-component one-pot reaction. First, we investigated different conditions to achieve this goal (Scheme 2, Table 1). Thus, the reaction (2.1:1:1 molar ratios) of **1**, different primary amines or ammonium acetate, and aromatic aldehydes in acetic acid under reflux (condition A) for 2–4 h gave the corresponding dihydropyridine derivatives **2a**–**o** in 66–86% yields. Conducting this reaction in a microwave at 150 °C increased the yields to 84–95%, decreased the reaction time to 2 min and also reduced the amount of the solvent used by ca. 90% (condition B, Scheme 2). Alternatively, compounds **2** were obtained also in good yield by reacting one equiv of the appropriate Schiff’s base **3** with two equiv of the enaminones **1** in acetic acid (condition C, Scheme 2). Table 1 summarizes the dihydropyridines prepared and the yields obtained under different reaction conditions shown in Scheme 2.

**Table 1:** Synthesis of dihydropyridine derivatives **2a**–**o**, reaction conditions and % yield.

Compound	R	Ar	Ar’	Conditions (% yield)

**2a**	H	C_6_H_5_	C_6_H_5_	A (68), B (94)
**2b**	H	C_6_H_5_	*p*-ClC_6_H_4_	A (70), B (92)
**2c**	H	C_6_H_5_	*p*-CH_3_C_6_H_4_	A (72)
**2d**	H	2-thienyl	C_6_H_5_	A (74)
**2e**	H	2-furyl	C_6_H_5_	A (75)
**2f**	C_6_H_5_	C_6_H_5_	C_6_H_5_	A (66), B (95), C (76)
**2g**	*p*-HOC_6_H_4_	C_6_H_5_	C_6_H_5_	A (85), B(93), C (74)
**2h**	C_6_H_5_	2-furyl	C_6_H_5_	A (68), B (91)
**2i**	*p*-CH_3_OC_6_H_4_	2-furyl	C_6_H_5_	A (66)
**2j**	*p*-CH_3_OC_6_H_4_	*p*-ClC_6_H_4_	C_6_H_5_	A (85)
**2k**	*p*-CH_3_OC_6_H_4_	2-thienyl	C_6_H_5_	A (86), B (92), C (85)
**2l**	C_6_H_5_	2-thienyl	C_6_H_5_	A (84), B (90), C (84)
**2m**	*o*-NCC_6_H_4_	C_6_H_5_	C_6_H_5_	C (77)
**2n**	*t*-butyl	C_6_H_5_	C_6_H_5_	A (78), B (84)
**2o**	CH_2_CO_2_H	C_6_H_5_	C_6_H_5_	A (73)

A: **1** (2.1 mmol), ArCHO (1 mmol), amine or ammonium acetate (1 mmol) in AcOH (10 mL) heated under reflux for 1–3 h; B: **1** (2.1 mmol), ArCHO (1 mmol), amine or ammonium acetate (1 mmol) in AcOH (1 mL) heated in MW at 150 °C for 2 min; C: **1** (2.2 mmol), **3** (1 mmol) and heating under reflux in AcOH for 1–3 h.

**Scheme 3:**
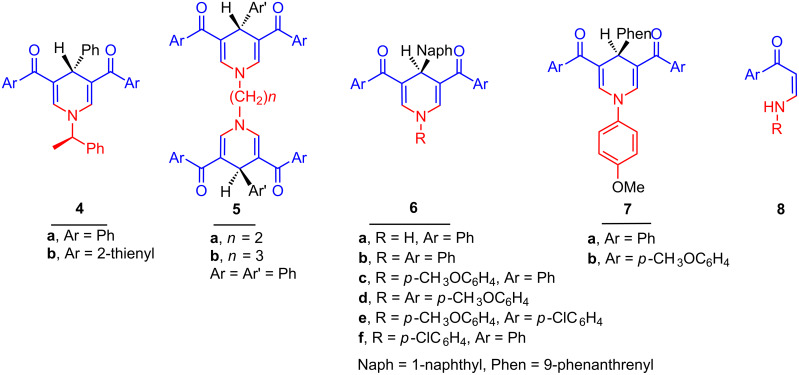
Dihydropyridine derivatives **4**–**7** and enaminone **8**.

**Figure 1:**
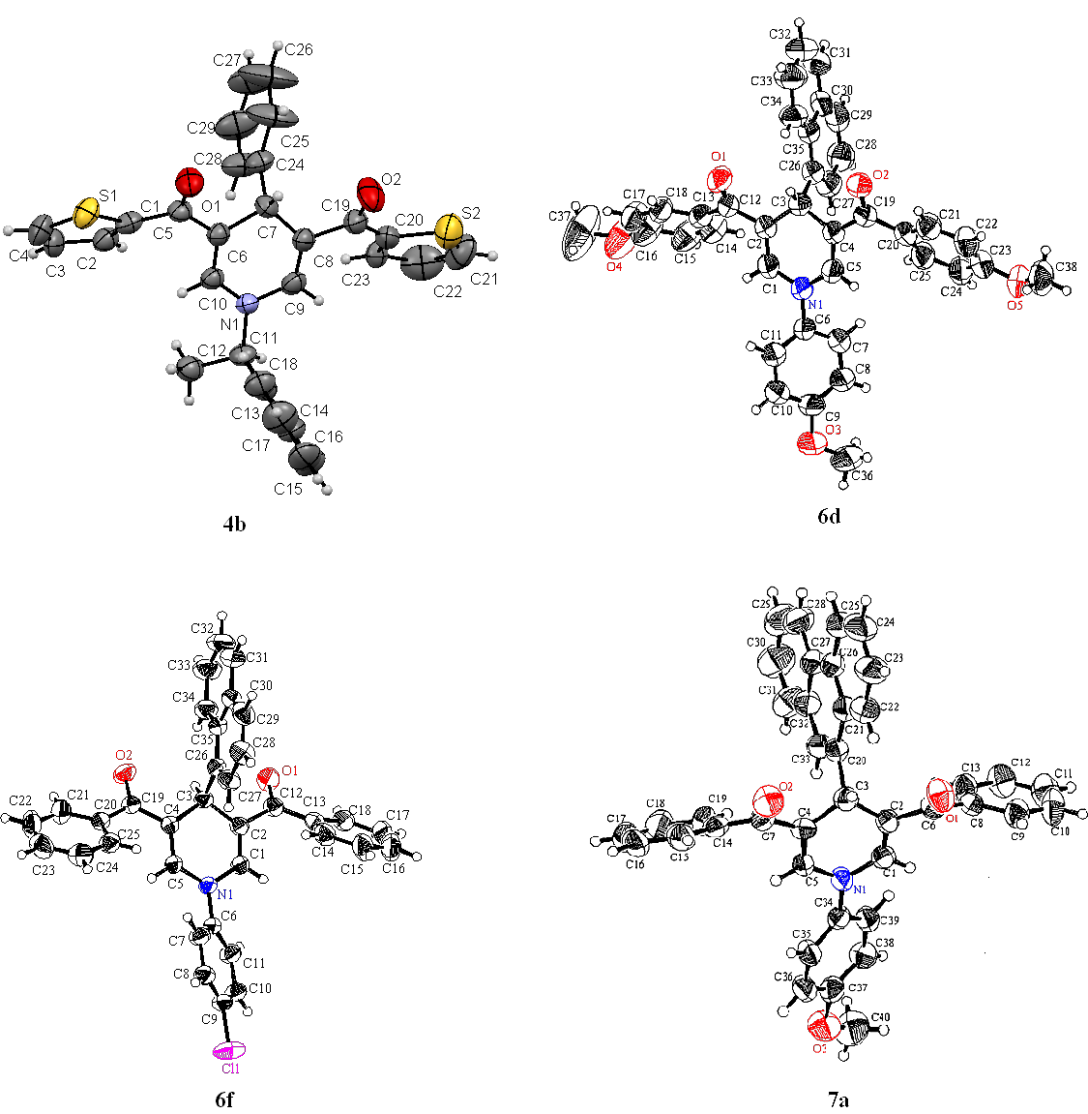
ORTEP of compounds **4b**, **6d**, **6f** and **7a**.

This study was extended to include the synthesis of the chiral (*R*)-1-(1-phenylethyl)dihydropyridines **4a**,**b** obtained in 78% yield by heating in acetic acid and in 93–94% yield by microwave irradiation with *R*-1-phenylethylamine in this three-component reaction. The bis(dihydropyridines) **5a**,**b** were obtained in 75–92% yield with ethylenediamine and 1,3-diaminopropane as the primary amines, respectively. The 4-(1-naphthyl)dihydropyridines **6a**–**f** and 4-(phenanthren-9-yl)dihydropyridine derivatives **7a**,**b** were obtained from 1-naphthaldehyde and phenanthrene-9-carboxaldehyde in moderate yields after heating in acetic acid for 24 h (Scheme 3). The intermediate *N*-substituted enaminones **8** were isolated as the main product when the reaction was conducted for shorter time [15]. The longer reaction time and the low yields are attributed to the steric hindrance of the bulky naphthyl and phenanthryl groups. The flanking dione groups in positions 3 and 5 keep the aryl groups in position 4 perpendicular to the dienaminoketone moiety of the dihydropyridine ring, and this is shown in the X-ray crystal structure of **4b**, **6d**,**f** and **7a** (Figure 1) [18].

**Figure 2:**
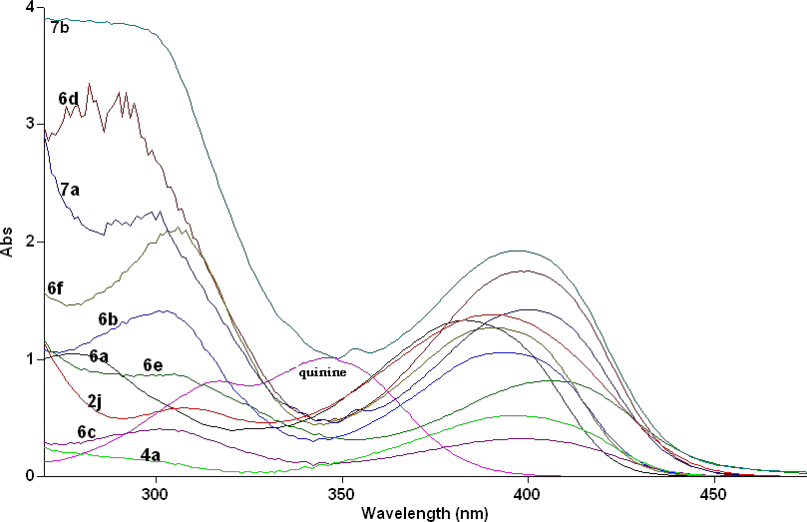
Absorption spectra of compounds **2j**, **6a**–**f** and **7a**,**b** in acetonitrile (1 × 10^−4^ M).

**Figure 3:**
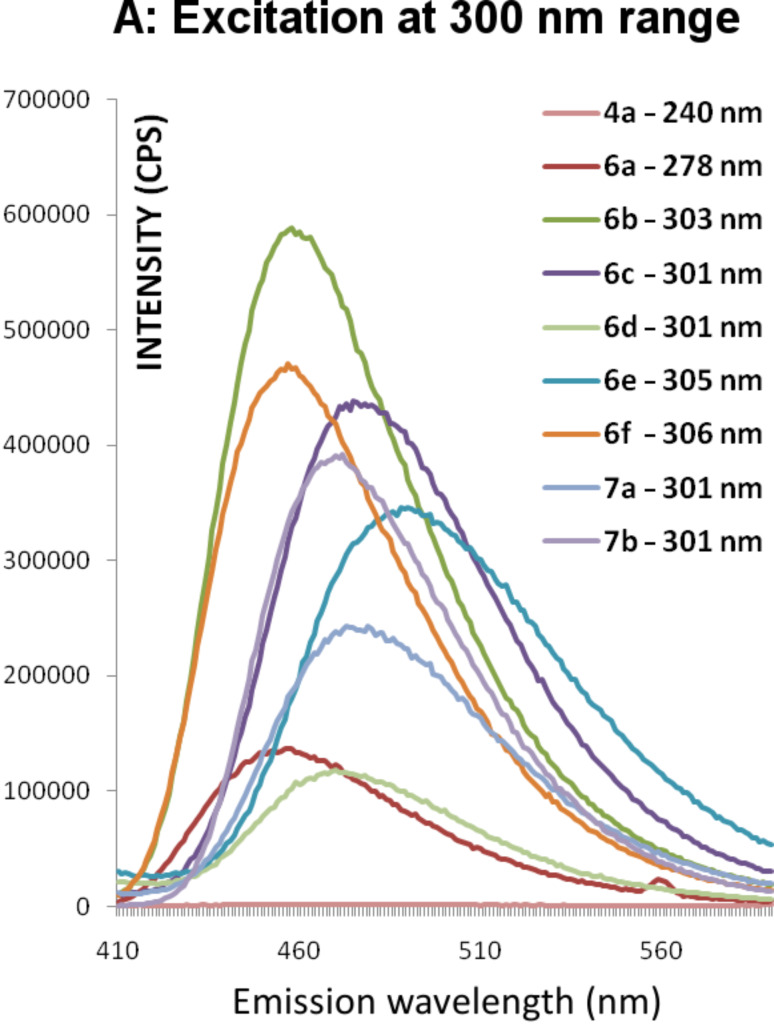
Emission spectra of compounds **4a**, **6a**–**f** and **7a**,**b** after excitation at their absorption λ_max_ in the range of 240–306 nm in acetonitrile (1 × 10^−4^ M).

**Figure 4:**
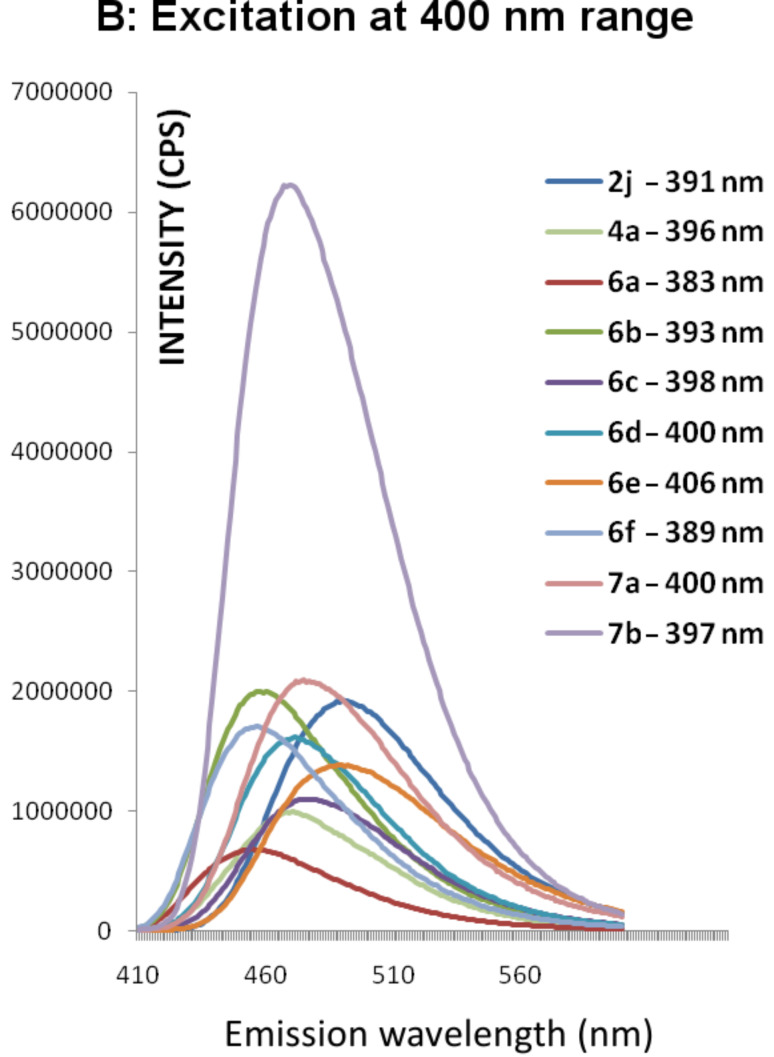
Emission spectra of compounds **2j**, **4a**, **6a**–**f** and **7a**,**b** after excitation at their absorption λ_max_ in the range of 383–406 nm in acetonitrile (1 × 10^−4^ M).

Compounds **6** and **7** are similar to the recently reported dihydropyridine dicarboxylate derivatives and are expected to act as photoinduced intramolecular electron-transfer systems [7–8]. Table 2 shows the UV–vis absorption–emission maxima of compounds **6a**–**f** and **7a**,**b**. The investigated compounds exhibit absorption spectra (Figure 2) with λ_max_ = 277–308 nm and 389–406 nm. The shorter absorption wavelength is attributable to the aryl groups and the longer absorption is due to the DHP moiety [8]. Upon excitation at each of these two λ_max_ these compounds gave fluorescence spectra (Figure 3 and Figure 4) with λ_max_ = 454–492 nm (Table 2). This photoluminescence behavior of **6** and **7** resembles that of dihydropyridinedicarboxylate derivatives [7–8], which suggests their potential application as suitable photoinduced intramolecular electron-transfer systems. For comparison the absorption and emission spectra of **2j** and **4a** have also been measured, and the results indicate weak emissions relative to **7b**. This compound, with the *p*-methoxyphenyl groups in the 1, 3 and 5 positions, showed the most intense absorption (Figure 2) and emission spectra (Figure 4) upon excitation in the 400 nm ranges. Relative fluorescence quantum yields (Table 2) were measured at 25 °C, taking quinine bisulfate (in 0.1 M H_2_SO_4_, 22 °C) as standard (Φ_f_ = 0.58 at λ_ex_ = 350 nm, Φ_f_ = 0.55 at λ_ex_ = 365 nm).

**Table 2:** The absorption and fluorescence of **6a**–**f**, **7a**,**b**, **2j** and **4a**.

Compound^a^	λ_max_^b^	log ε_max_	λ_em_^c^	Φ_f_^d^	Φ_f_^e^

**6a**	383 277	4.125 4.019	454 456	0.0176	0.007
**6b**	393 303	4.024 4.149	457 457	0.045	0.024
**6c**	398 301	3.509 3.608	476 476	0.050	0.030
**6d**	400 294	4.243 4.502	467 469	0.013	0.009
**6e**	406 305	3.911 3.941	486 488	0.035	0.025
**6f**	389 306	4.102 4.327	456 456	0.034	0.017
**7a**	400 301	4.152 4.354	475 475	0.024	0.014
**7b**	397 301	4.284 4.590	466 475	0.096	0.057
**2j**	391 308	4.140 3.766	492	0.034	0.015
**4a**	396 240	3.716 3.745	468	0.035	0.021

^a^All spectra were measured for a 1 × 10^−4^ M solution in acetonitrile; ^b^absorption and excitation; ^c^emission; ^d^taking quinine bisulfate Φ_f_ = 0.58 as standard at λ_ex_ 350 nm; ^e^taking quinine bisulfate Φ_f_ = 0.55 at λ_ex_ 365 nm.

**Scheme 4:**
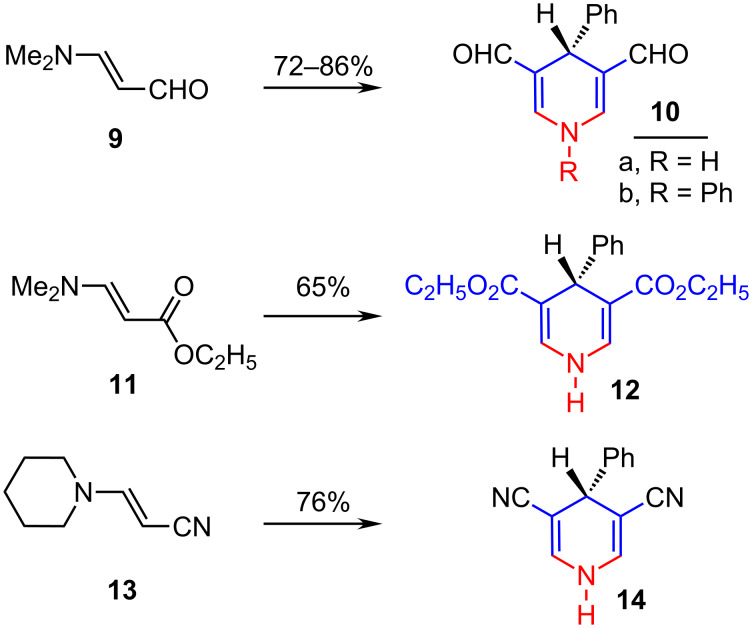
Synthesis of dihydropyridines from an enamino aldehyde, an enamino ester and an enaminonitrile.

This synthesis of dihydropyridines was extended to enamino aldehyde **9**, enamino ester **11** and enaminonitrile **13**. Thus, 1,4-dihydropyridine-3,5-dicarboxaldehyde **10a**,**b**, 1,4-dihydropyridine-3,5-dicarboxylate **12** and 1,4-dihydropyridine-3,5-dicarbonitrile **14** were successfully obtained by reacting β-*N,N*-dimethylaminoacrolein (**9**), ethyl β-*N,N*-dimethylaminoacrylate (**11**) or β-piperidinoacrylonitrile (**13**) with the appropriate aldehyde and primary amine under the same reaction conditions (A, B) (Scheme 4).

**Scheme 5:**
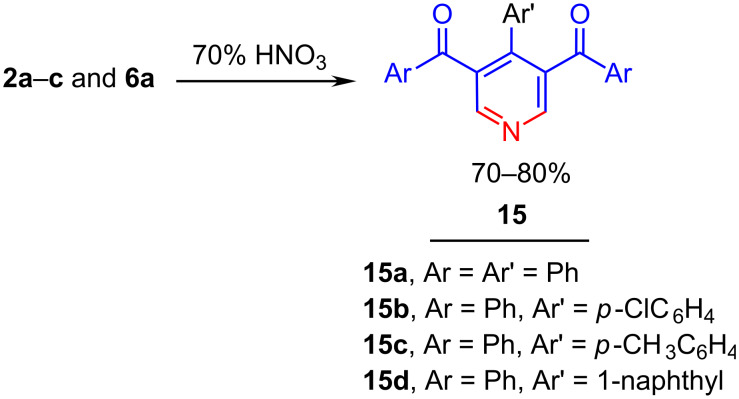
Nitric acid oxidation of dihydropyridines **2a**–**c** and **6a**.

**Figure 5:**
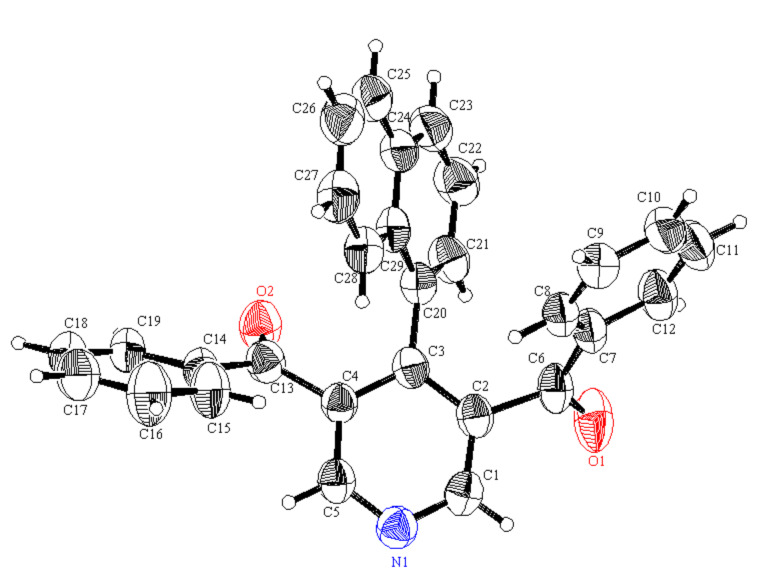
ORTEP of compound **15d**.

Compounds **2a**–**c** and **6a** were readily oxidized to the corresponding pyridine derivatives **15a**–**d** by stirring in aqueous nitric acid (70%) at 5 °C to room temperature (Scheme 5). The X-ray structure data of **15d** (Figure 5) [18] indicates the nonplanarity of the different aryl groups with respect to any of the conjugated systems involved in the pyridine ring.

## Conclusion

The present work offers an alternative and efficient method for the synthesis of dihydropyridines with potentially wide applicability, compared to the recently reported [14] synthesis of 3,5-dibenzoyl-1,4-disubstituted-dihydropyridine derivatives. The present method has the following advantages:

1. The starting enaminones **1** can be readily synthesized from any methylketone, whereas the reported method is limited to arylpropynones.
2. This is a one-pot three-component reaction; on the other hand, the reported method involves two steps starting with the reaction of phenylpropynone with a primary amine, followed by reaction with different aldehydes.
3. The synthesis of suitable substituted derivatives, such as **6** and **7**, possessing interesting fluorescence and structural characteristics for remarkable photoluminescence behavior, which suggests their potential application as suitable photoinduced intramolecular electron-transfer systems.
4. This method can be extended to the synthesis of enaminoaldehydes **10**, enaminoesters **12** and enaminonitriles **14**.

## Experimental

General: All melting points are uncorrected. The microwave oven used was a single-mode cavity explorer microwave (CEM Corporation, NC, USA) and irradiation was conducted in heavy-walled pyrex tubes (capacity 10 mL). IR spectra were recorded in KBr disks on a Perkin Elmer System 2000 FTIR spectrophotometer. ^1^H and ^13^C NMR spectra were recorded on Bruker DPX 400, 400 MHz, Avance II 600, 600 MHz super-conducting NMR spectrometers. Mass spectra were measured on GCMSDFS-Thermo and with LCMS by using Agilent 1100 series LC/MSD with an API-ES/APCI ionization mode. Microanalyses were performed on LECO CH NS-932 Elemental Analyzer. The UV–vis absorption spectra were scanned by using a Varian Cary 5 instrument in the wavelength range 250–450 nm with dry, clean quartz cuvettes of 1.0 cm path length. From the spectra obtained, absorbance values at λ_max_ were used to calculate the extinction coefficient. The emission spectra were measured at the same concentration after excitation at the specified λ shown in Figure 2, by using a Horiba-Jobin Vyon Fluromax-4 instrument. Relative fluorescence quantum yields were measured at 25 °C taking quinine bisulfate (in 0.1 M H_2_SO_4_, 22 °C) as standard (Φ_f_ = 0.58 at λ_ex_ = 350 nm, Φ_f_ = 0.55 at λ_ex_ = 365 nm) [19]. X-rays structures were determined by single-crystal X-ray crystallography RIGAKU RAPID II. Enaminones **1** were prepared according to the previously reported procedure [16–17] and compound **8** was identical with an authentic sample that was prepared as reported [15].

## Supporting Information

File 1 Experimental procedures and characterization of compounds, including copies of ^1^H and ^13^C NMR spectra.
