# Supplementary material for: Enaminones in a multicomponent synthesis of 4-aryldihydropyridines for potential applications in photoinduced intramolecular electron-transfer systems
Source: Beilstein J Org Chem. 2012 Mar 26;8:441–7. doi: 10.3762/bjoc.8.50 (PMC3326623; doi:10.3762/bjoc.8.50)
Supplement: File 1 — Experimental procedures and characterization of compounds, including copies of 1H and 13C NMR spectra. [file Beilstein_J_Org_Chem-08-441-s001.pdf]

Supporting Information

for

Enaminones in a multicomponent synthesis of 4-aryldihydropyridines for potential applications in photoinduced intramolecular electron-transfer systems

Nouria A. Al-Awadi*, Maher R. Ibrahim, Mohamed H. Elnagdi, Elizabeth John and Yehia A. Ibrahim

Address: Chemistry Department, Faculty of Science, Kuwait University, P.O. Box 5969, Safat 13060, Kuwait

Email: Nouria A. Al-Awadi - n.alawadi@ku.edu.kw
*Corresponding author

Experimental procedures and characterization of compounds, including copies of ^{1}H and ^{13}C NMR spectra.

Contents

Experimental details, analytical and spectral data of products ……………….S2–S14
^{1}H NMR and ^{13}C NMR of all products ……………………………………...S15–S49

Synthesis

Synthesis of dihydropyridines **2**, **4**, **10**, **12** *and* **14**: *General procedure*

Method A: A mixture of the appropriate enaminones **1** (2.2 mmol) [1,2], aromatic aldehyde (1 mmol) and ammonium acetate (0.228 g, 1.5 mmol), or aromatic amine (1 mmol) in glacial acetic acid (20 mL) was heated under reflux for 1–3 h. The mixture was then poured into ice water (ca. 50 g) and the precipitate was collected by filtration and crystallized from the proper solvent to yield compounds **2a–o** (Table 1), **4a**,**b**, **10a**,**b**, **12** and **14**.

Method B: A mixture of the starting material in few drops of acetic acid (0.5 mL) was irradiated in a microwave oven for 2 min at 160 °C, and after cooling, ice water (5 mL) was added. The precipitated product was collected and crystallized from the proper solvent to give **2**, (Table 1) **4a**,**b** and **10b**.

Method C: A mixture of the appropriate enaminones **1** (2.2 mmol) and the appropriate Schiff's base **3** (1.0 mmol) in acetic acid (20 mL) was heated under reflux for 2 h. After cooling, ice water (50 mL) was added and the yellow precipitate formed was collected and crystallized from ethanol to give **2** (Table 1).

3,5-Dibenzoyl-4-phenyl-1,4-dihydropyridine (**2a**). Yellow needles from ethyl acetate; mp 245–246 °C (lit. [3] mp 246–248 °C). MS: *m/z* = 365 (M^+, 80%), 288 (100%). LCMS: *m*/*z* = 366 (M + 1); IR: 3412, 3241, 3031, 2963, 1633, 1614, 1571, 1490, 1371, 1220, 1115, 1080, 968; ^{1}H NMR (400 MHz, DMSO-d_6): δ 9.28 (t, 1H, NH, *J* = 5.6), 7.54–7.50 (m, 2H), 7.44 (m, 8H), 7.35 (d, 2H, *J* = 7.4), 7.27 (t, 2H, *J* = 7.6), 7.17–7.13 (m, 1H), 7.13 (d, 2H, *J* = 5.6), 5.41 (s, 1H); ^{13}C NMR (100 MHz, 100 MHz, DMSO-d_6): δ 193.6, 146.8, 139.29, 139.27, 130.7, 128.3, 128.1, 128.0, 127.6, 126.0, 116.3, 35.7. Anal. calc. for $C_{25}H_{19}NO_2$ (365.4): C 82.17; H 5.24; N 3.83. Found: C 82.08; H 5.18; N 3.77.

4-p-Chlorophenyl-3,5-dibenzoyl-1,4-dihydropyridine (**2b**). Yellow needles from ethyl acetate, mp 232–234 °C. LCMS = 400 (M + 1); MS: *m/z* = 401 (M + 2, 32%), 399 (M^+, 100%), 288 (80%), 105 (25%); IR: 3417, 3236, 3160, 3044, 1655, 1621, 1566, 1481, 1369, 1224, 1176, 1084, 970, 727; ^{1}H NMR (400 MHz, $CDCl_3$): δ 7.45–7.41 (m, 6H), 7.38–7.33 (m, 6H), 7.18 (d, 2H, *J* = 8.4), 6.93 (d, 2H, *J* = 8.4), 6.76 (t, 1H, *J* = 4.8), 5.56

(s, 1H); ^{13}C NMR (100 MHz, $CDCl_3$): δ 194.6, 144.7, 139.1, 137.4, 132.1, 131.0, 129.5, 128.4, 128.33, 128.26, 118.4, 36.2; Anal. calc. for $C_{25}H_{18}ClNO_2$ (399.9): C 75.09; H 4.54; N 3.50. Found: C 75.10; H 4.39; N 3.47.

3,5-Dibenzoyl-4-p-tolyl-1,4-dihydropyridine (**2c**). Yellow needles from ethanol, mp 228–230 °C. MS: *m*/*z* = 379 (M^+, 85%), 288 (100%), 105 (80%); IR: 3430, 3240, 3151, 3031, 2995, 1629, 1560, 1474, 1369, 1223, 1176, 1116, 969, 762; ^{1}H NMR (400 MHz, $CDCl_3$): δ 7.45–7.39 (m, 6H), 7.34 (d, 2H, *J* = 8.0), 7.30–7.26 (m, 4H), 7.01 (d, 2H, *J* = 8.0), 6.89 (d, 2H, *J* = 5.2), 6.75 (t, 1H, *J* = 5.2), 5.54 (s, 1H), 2.23 (s, 3H); ^{13}C NMR (100 MHz, DMSO-d_6): δ 194.3, 144.5, 139.8, 139.6, 135.4, 131.2, 129.2, 128.8, 128.5, 128.1, 116.9, 35.8, 21.1. Anal. calc. for $C_{26}H_{21}NO_2$ (379.5): C 82.30; H 5.58; N 3.69. Found: C 82.18; H 5.39; N 3.67.

3,5-Bis(2-thiophenecarbonyl)-4-phenyl-1,4-dihydropyridine (**2d**). Yellow needles from ethanol, mp 315–317 °C. MS: *m*/*z* = 377 (M^+, 90%), 300 (100%), 111 (35%); IR: 3413, 3100, 3022, 1661, 1591, 1468, 1412, 1365, 1290, 1199, 1086, 742, 719; ^{1}H NMR (400 MHz, DMSO-d_6): δ 9.41 (br, 1H, NH), 7.85 (dd, 2H, *J* = 4.8, 1.2), 7.58 (dd, 2H, *J* = 3.6, 1.2), 7.55 (br, 2H), 7.28 (dd, 2H, *J* = 7.6, 1.2), 7.21 (t, 2H, *J* = 7.6), 7.15 (dd, 2H, *J* = 4.8, 3.6), 7.08 (t, 1H, *J* = 7.8), 5.36 (s, 1H); ^{13}C NMR (100 MHz, DMSO-d_6): δ 184.4, 146.6, 143.4, 137.5, 132.2, 131.3, 128.0, 127.9, 127.8, 126.1, 115.9, 36.7. Anal. calc. for $C_{21}H_{15}NO_2S_2$ (377.5): C 66.82; H 4.01; N 3.71; S 16.99. Found: C 66.78; H 4.08; N 3.70; S 16.98.

3,5-Bis(2-furoyl)-4-phenyl-1,4-dihydropyridine (**2e**). Yellow crystals from ethanol, mp 238–240 °C. MS: *m*/*z* = 345 (M^+, 75%), 224 (100%), 168 (25%); IR: 3327, 3106, 2946, 1698, 1664, 1598, 1404, 1318, 1206, 1118, 819; ^{1}H NMR (400 MHz, DMSO-d_6): δ 9.56 (t, 1H, *J* = 5.4), 7.89 (d, 2H, *J* = 0.6), 7.85 (d, 2H, *J* = 5.4), 7.25 (d, 2H, *J* = 8.4), 7.19 (t, 2H, *J* = 8.0), 7.11 (d, 2H, *J* = 3.0), 7.07 (t, 1H, *J* = 7.8), 6.63 (dd, 2H, *J* = 3.0, 0.6), 5.34 (s, 1H); ^{13}C NMR (100 MHz, DMSO-d_6): δ 178.8, 152.3, 146.7, 146.1, 137.6, 128.0, 127.7, 126.0, 116.9, 115.6, 111.9, 35.4. Anal. calc. for $C_{21}H_{15}NO_4$ (345.4): C 73.04; H 4.38; N 4.06. Found: C 73.00; H 4.28; N 4.00.

3,5-Dibenzoyl-1,4-diphenyl-1,4-dihydropyridine (**2f**). Yellow needles from ethanol, mp 296–298 °C. MS: *m*/*z* = 441 (M^+, 80%), 364 (100%), 105 (50%); IR: 3058, 3024, 2894, 1657, 1624, 1592, 1568, 1491, 1448, 1344, 1285, 1236, 1144, 1071, 978, 695; ^{1}H NMR (400 MHz, $CDCl_3$): δ 7.59–7.55 (m, 4H), 7.52–746 (m, 4H), 7.44–7.39 (m, 6H), 7.32 (s, 2H), 7.29 (m, 3H), 7.17 (m, 3H), 5.72 (s, 1H); ^{13}C NMR (100 MHz, DMSO-d_6): δ 193.9, 145.5, 142.9, 139.6, 138.6, 131.4, 130.0, 128.5, 128.34, 128.28, 128.0, 126.8, 126.4, 121.3, 118.8, 36.3. Anal. calc. for $C_{31}H_{23}NO_2$ (441.5): C 84.33; H 5.25 ; N 3.17. Found: C 84.25; H 5.21 ; N 3.12.

3,5-Dibenzoyl-1-(p-hydroxyphenyl)-4-phenyl-1,4-dihydropyridine (**2g**). Yellow needles from ethanol, mp 330–332 °C. MS: *m*/*z* = 457 (M^+, 80%), 380 (100%), 105 (50%); IR: 3257, 3057, 3030, 2971, 1656, 1626, 1552, 1512, 1448, 1337, 1281, 1236, 1147, 1115, 980, 719; ^{1}H NMR (400 MHz, DMSO-d_6): δ 9.72 (s, 1H, OH), 7.53 (dt, 6H, *J* = 8.4, 1.2), 7.43 (dt, 6H, *J* = 8.4, 1.2), 7.30 (t, 2H, *J* = 8.0), 7.24 (d, 2H, *J* = 8.4), 7.17 (s, 2H), 7.14 (t, 1H, *J* = 7.8), 6.76 (d, 2H, *J* = 8.4), 5.46 (s, 1H); ^{13}C NMR (150 MHz, DMSO-d_6): δ 193.8, 156.5, 145.8, 140.6, 138.7, 134.9, 131.3, 128.5, 128.3, 128.2, 128.0, 126.4, 123.6, 118.1, 116.2, 36.0. Anal. calc. for $C_{31}H_{23}NO_3$ (457.5): C 81.38; H 5.07; N 3.06. Found: C 81.25; H 5.01 ; N 3.10.

3,5-Bis(2-furoyl)-1,4-diphenyl-1,4-dihydropyridine (**2h**). Yellow needles from ethanol, mp 229–230 °C. MS: *m*/*z* = 421 (M^+, 80%), 364 (100%), 105 (50%); IR: 3090, 3022, 2914, 1662, 1618, 1598, 1563, 1491, 1463, 1348, 1299, 1245, 1168, 1117, 1074, 740; ^{1}H NMR (400 MHz, DMSO-d_6): δ 8.06 (s, 2H), 7.93 (dd, 2H, *J* = 1.8, 0.8), 7.66 (d, 2H, *J* = 7.6), 7.54 (t, 2H, *J* = 7.8), 7.38 (t, 1H, *J* = 8.0), 7.35 (d, 2H, *J* = 8.8), 7.28 (dd, 2H, *J* = 1.6, 0.8), 7.23 (t, 2H, *J* = 7.8), 7.11 (t, 1H, *J* = 7.6), 6.64 (dd, 2H, *J* = 3.6, 1.6), 5.41 (s, 1H); ^{13}C NMR (100 MHz, DMSO-d_6): δ 179.0, 151.9, 146.8, 145.4, 143.1, 138.1, 130.1, 128.2, 128.0, 126.7, 126.4, 121.4, 118.2, 118.0, 112.1, 35.9. Anal. calc. for $C_{27}H_{19}NO_4$ (421.5): C 76.95; H 4.54; N 3.32. Found: C 76.90; H 4.51; N 3.22.

3,5-Bis(2-furoyl)-1-(p-methoxyphenyl)-4-phenyl-1,4-dihydropyridine (**2i**). Yellow needles from ethanol, mp 220–222 °C. MS: *m*/*z* = 451 (M^+, 90%), 374 (100%), 331 (25%); IR: 3122, 3012, 2928, 1657, 1610, 1560, 1512, 1337, 1292, 1246, 1170, 1115, 1077, 746; ^{1}H NMR (400 MHz, DMSO-d_6): δ 7.96 (s, 2H), 7.92 (dd, 2H, *J* = 1.6, 0.8), 7.60 (dd, 2H, *J* = 8.0. 1.6), 7.36 (dd, 2H, *J* = 8.0, 1.2), 7.26 (dd, 2H, *J* = 3.6, 0.8), 7.23 (t, 2H, *J* = 8.0), 7.11 (t, 1H, *J* = 7.4), 7.08 (dd, 2H, *J* = 8.0, 1.6), 6.65 (dd, 2H, *J* = 3.6, 1.6), 5.40 (s, 1H), 3.81 (s, 3H, OCH_3); ^{13}C NMR (100 MHz, DMSO-d_6): δ 178.9, 158.1, 151.9, 146.7, 145.6, 138.9, 136.5, 128.2, 128.0, 126.3, 123.5, 117.8, 117.6, 115.1, 112.1, 55.5, 35.6. Anal. calc. for $C_{28}H_{21}NO_5$ (451.5): C 74.49; H 4.69; N 3.10. Found: C 74.40; H 4.51; N 3.02.

3,5-Bis(p-chlorobenzoyl)-1-(p-methoxyphenyl)-4-phenyl-1,4-dihydropyridine (**2j**). Yellow needles from ethanol, mp 196–198 °C. MS: *m*/*z* = 543 (M + 4, 8%), 541 (M + 2, 50%), 539 (M^+, 94%), 462 (100%), 400 (20%); IR: 3061, 3024, 2958, 1626, 1590, 1508, 1476, 1457, 1289, 1237, 1085, 826, 747, 692; ^{1}H NMR (400 MHz, DMSO-d_6): δ 7.58 (d, 4H, *J* = 8.4), 7.50 (d, 4H, *J* = 8.4), 7.43 (t, 2H, *J* = 7.4), 7.40 (d, 2H, *J* = 8.8), 7.30 (t, 2H, *J* = 7.8), 7.27 (s, 2H), 7.16 (t, 1H, *J* = 7.4), 6.97 (d, 2H, *J* = 8.8), 5.44 (s, 1H), 3.75 (s, 3H); ^{13}C NMR (100 MHz, DMSO-d_6): δ 192.6, 158.0, 145.5, 140.6, 137.3, 136.1, 136.0, 130.2, 128.5, 128.3, 128.0, 126.4, 123.4, 118.1, 114.9, 55.5, 36.0. Anal. calc. for $C_{32}H_{23}Cl_2NO_3$ (540.5): C 71.12; H 4.29; N 2.59. Found: C 71.05; H 4.21; N 2.52.

3,5-Bis(2-thiophenecarbonyl)-1-(p-methoxyphenyl)-4-phenyl-1,4-dihydropyridine (**2k**). Yellow needles from ethanol, mp 228–230 °C. MS: *m*/*z* = 483 (M^+, 90%), 406 (100%), 111 (60%); IR: 3100, 3024, 2914, 1661, 1610, 1569, 1510, 1415, 1338, 1285, 1226, 1122, 1073, 1024, 822, 738; ^{1}H NMR (400 MHz, DMSO-d_6): δ 7.90 (dd, 2H, *J* = 4.8, 0.8), 7.76 (dd, 2H, *J* = 3.6, 0.8), 7.65 (s, 2H), 7.57 (d, 2H, *J* = 8.8), 7.40 (d, 2H, *J* = 8.0), 7.26 (t, 2H, *J* = 7.6), 7.16 (dd, 2H, *J* = 4.8, 3.6), 7.12 (t, 1H, *J* = 7.8), 7.03 (d, 2H, *J* = 8.8), 5.43 (s, 1H), 3.79 (s, 3H); ^{13}C NMR (100 MHz, DMSO-d_6): δ 184.8, 157.9, 145.5, 143.2, 138.6, 136.5, 133.0, 132.3, 128.22, 128.15, 128.06, 126.4, 123.4, 118.0, 114.9, 55.5, 37.1. Anal. calc. for $C_{28}H_{21}NO_3S_2$ (483.6): C 69.54; H 4.38; N 2.90; S 13.26. Found: C 69.45; H 4.31; N 2.82; S 13.21.

3,5-Bis(2-thiophenecarbonyl)-1,4-diphenyl-1,4-dihydropyridine (**2l**). Yellow needles from ethanol, mp 298–300 °C. MS: *m*/*z* = 453 (M^+, 90%), 376 (100%), 342 (20%); IR: 3092, 3023, 2920, 1659, 1595, 1569, 1491, 1415, 1342, 1286, 1232, 1122, 1070, 852, 740; ^{1}H NMR (400 MHz, DMSO-d_6): δ 7.91 (dd, 2H, *J* = 4.8, 1.2), 7.79 (dd, 2H, *J* = 3.6, 1.2), 7.75 (s, 2H), 7.63 (d, 2H, *J* = 7.8), 7.51 (t, 2H, *J* = 8.0), 7.40 (d, 2H, *J* = 7.6), 7.36 (t, 1H, *J* = 7.4), 7.25 (t, 2H, *J* = 7.8), 7.16 (dd, 2H, *J* = 4.8, 3.6), 7.12 (t, 1H, *J* = 7.8), 5.44 (s, 1H); ^{13}C NMR (100 MHz, DMSO-d_6): δ 184.9, 145.2, 143.1, 143.0, 137.8, 133.1, 132.4, 129.9, 128.21, 128.19, 128.0, 126.5, 126.4, 121.3, 118.5, 37.2. Anal. calc. for $C_{27}H_{19}NO_2S_2$ (453.6): C 71.50; H 4.22; N 3.09; S 14.14. Found: C 71.45; H 4.17 ; N 3.02; S 14.11.

3,5-Dibenzoyl-1-(o-cyanophenyl)-4-phenyl-1,4-dihydropyridine (**2m**). Yellow needles from ethanol, mp 225–227 °C. MS: *m*/*z* = 466 (M^+, 100%), 389 (100%), 105 (20%); IR: 3063, 3024, 2971, 2223, 1638, 1571, 1489, 1333, 1275, 1229, 1089, 787, 754; ^{1}H NMR (600 MHz, DMSO-d_6): δ 7.93 (d, 1H, *J* = 8.8), 7.74 (t, 1H, *J* = 7.6), 7.62 (d, 1H, *J* = 8.0), 7.58 (d, 4H, *J* = 7.8), 7.53–7.49 (m, 5H), 7.42 (t, 4H, *J* = 7.8), 7.29 (t, 2H, *J* = 7.6), 7.27 (s, 2H), 7.17 (t, 1H, *J* = 7.8), 5.54 (s, 1H); ^{13}C NMR (100 MHz, DMSO-d_6): δ 193.9, 145.4, 145.3, 140.1, 138.3, 135.0, 134.2, 131.6, 128.5, 128.4, 128.3, 128.21, 128.18, 126.6, 125.7, 118.7, 116.6, 108.0, 36.3. Anal. calc. for $C_{32}H_{22}N_2O_2$ (466.5): C 82.38; H 4.75; N 6.00. Found: C 82.25; H 4.71 ; N 6.02.

3,5-dibenzoyl-4-phenyl-1-tert-butyl-1,4-dihydropyridine (**2n**). Yellow needles from ethanol, mp 218–220 °C. MS: *m*/*z* = 421 (M^+, 90%), 364 (80%), 288 (100%); IR: 3082, 3033, 2913, 1671, 1593, 1556, 1443, 1410, 1352, 1256, 1212, 1024, 1070, 864, 737; ^{1}H NMR (400 MHz, DMSO-d_6): δ 7.56–7.52 (m, 2H), 7.50–7.44 (m, 8H), 7.31 (d, 2H, *J* = 7.6), 7.25 (s, 2H), 7.23 (t, 2H, *J* = 7.8), 7.13 (t, 1H, *J* = 7.8), 5.38 (s, 1H), 1.30 (s, 9H); ^{13}C NMR (100 MHz, $CDCl_3$): δ 194.5, 146.1, 139.6, 138.0, 130.8, 128.5, 128.3, 128.2, 127.9, 126.3, 119.2, 57.9 36.7, 29.2. Anal. calc. for $C_{29}H_{27}NO_2$ (421.5): C 82.63; H 6.46; N 3.32. Found: C 82.55; H 6.41; N 3.30.

(3,5-Dibenzoyl-4-phenyl-4H-pyridin-1-yl)acetic acid (**2o**). Yellow needles from ethyl acetate, mp 258–260 °C. MS: *m/z* = 423 (M^+, 90%), 364 (80%), 288 (100%); IR: 3082, 3033, 2913, 1671, 1593, 1556, 1443, 1410, 1352, 1256, 1212, 1024, 1070, 864, 737; ^{1}H NMR (400 MHz, $CDCl_3$): δ 7.52–7.42 (m, 4H), 7.39 (t, 4H, *J* = 7.8), 7.31 (t, 4H, *J* = 7.6), 7.19 (t, 2H, *J* = 7.8), 7.05 (t, 1H, *J* = 7.8), 6.76 (s, 2H), 5.52 (s, 1H), 4.06 (s, 2H), 2.91 (br, 1H); ^{13}C NMR (150 MHz, $CDCl_3$): δ 194.7, 170.3, 145.7, 141.6, 139.0, 131.1, 128.6, 128.34, 128.29, 128.25, 126.4, 119.2, 55.5, 36.5. Anal. calc. for $C_{27}H_{21}NO_4$ (423.5): C 76.58; H 5.00; N 3.31. Found: C 76.55; H 5.01; N 3.30.

3,5-Dibenzoyl-4-phenyl-1-(1-phenylethyl)-1,4-dihydropyridine (**4a**). Yellow needles from ethanol, yield 78% (method A), 94% (method B), mp 289–290 °C. MS: *m/z* = 469 (M^+, 80%), 364 (95%), 288 (50%); IR: 3095, 3027, 2983, 1659, 1599, 1563, 1411, 1365, 1287, 1230, 1201, 1090, 939, 734; ^{1}H NMR (400 MHz, DMSO-d_6): δ 7.44–7.38 (m, 11H), 7.32 (t, 4H, *J* = 7.8), 7.18 (t, 4H, *J* = 7.8), 7.13 (t, 1H, *J* = 7.6), 6.97 (dd, 2H, *J* = 3.6, 1.2), 5.60 (s, 1H), 4.71 (q, 1H, *J* = 7.0), 1.67 (d, 3H, *J* = 7.0); ^{13}C NMR (100 MHz, DMSO-d_6): δ 194.3, 194.1, 146.0, 139.8, 139.5, 139.4, 139.3, 129.2, 130.9, 130.8, 129.2, 128.6, 128.5, 128.3, 128.1, 128.0, 126.6, 126.4, 119.2, 118.9, 62.3, 37.1, 19.6. Anal. calc. for $C_{33}H_{27}NO_2$ (469.6): C 84.41; H 5.80; N 2.98. Found: C 84.45; H 5.71; N 3.00.

3,5-Bis(2-thiophenecarbonyl)-1-(1-phenylethyl)-4-phenyl-1,4-dihydropyridine (**4b**). Yellow needles from ethanol, yield 78% (method A), 93% (method B), mp 310–312 °C. MS: *m/z* = 481 (M^+, 100%), 376 (100%), 300 (70%); IR: 3055, 3022, 2938, 1661, 1624, 1566, 1450, 1418, 1360, 1275, 1248, 1192, 1113, 972, 786, 756, 727; ^{1}H NMR (400 MHz, $CDCl_3$): δ 7.85 (dd, 2H, *J* = 3.6, 1.2), 7.54–7.42 (m, 6H), 7.39 (t, 1H, *J* = 7.8), 7.36 (dd, 1H, *J* = 3.6, 1.2), 7.30 (dd, 1H, *J* = 3.6, 1.2), 7.23–7.17 (m, 4H), 7.12–6.98 (m, 3H), 5.32 (q, 1H, *J* = 7.0), 5.27 (s, 1H), 1.75 (d, 3H, *J* = 7.0); ^{13}C NMR (100 MHz, DMSO-d_6): δ 184.3, 184.1, 145.9, 143.4, 143.3, 140.1, 139.0, 138.5, 132.7, 132.6, 131.58, 131.55, 129.0, 128.2, 128.1, 127.9, 127.7, 127.2, 126.3, 116.9, 116.7, 61.0, 37.2, 18.7. Anal. calc. for $C_{29}H_{23}NO_2S_2$ (481.6): C 72.32; H 4.81; N 2.91; S 13.31. Found: C 72.25; H 4.74; N 2.82 ; S 13.30.

Synthesis of pyridine derivatives **5a**,**b**: *General Procedure*

A mixture of each of the appropriate enaminones **1** (4.2 mmol), benzaldehyde (0.22 g, 2 mmol) and the appropriate aliphatic diamine (1 mmol) was heated under reflux in acetic acid (20 mL) for 2 h (method A), or irradiated in a microwave oven for 2 min at 160 °C (method B), and after cooling, ice water was added to the mixture. The yellow solid so formed was collected and crystallized from ethanol to give **5a**,**b** in excellent yield.

1,2-Bis(3,5-dibenzoyl-4-phenyl-1,4-dihydropyridin-1-yl)ethane (**5a**). Yellow needles from ethanol, yield 76% (method A), 92% (method B), mp 279–280 °C. MS: *m*/*z* = 756 (M^+, 30%), 738 (25%), 365 (50%), 105 (100%); IR: 3055, 2938, 1661, 1624, 1566, 1450, 1410, 1366, 1285, 1248, 1192, 1113, 972, 786, 756, 727; ^{1}H NMR (400 MHz, DMSO-d_6): δ 7.47 (tt, 4H, *J* = 7.8, 2.0), 7.40–7.29 (m, 20H), 7.18 (t, 4H, *J* = 7.8), 7.09 (tt, 2H, *J* = 7.8, 1.4), 7.04 (s, 4H), 5.28 (s, 2H), 3.78 (s, 4H); ^{13}C NMR (100 MHz, DMSO-d_6): δ 193.1, 146.3, 142.0, 138.8, 130.9, 128.3, 128.1, 128.0, 127.8, 126.1, 117.3, 52.8, 35.7. Anal. calc. for $C_{52}H_{40}N_2O_4$ (756.9): C 82.52; H 5.33; N 3.70. Found: C 82.45; H 5.31; N 3.72.

1,3-Bis(3,5-dibenzoyl-4-phenyl-1,4-dihydropyridin-1-yl)propane (**5b**). Yellow needles from ethanol, yield 75% (method A), 91% (method B) mp 310–312 °C. MS: *m*/*z* = 770 (M^+, 20%), 752 (35%), 302 (55%), 105 (100%); IR: 3055, 2938, 1661, 1624, 1566, 1450, 1410, 1366, 1285, 1248, 1192, 1113, 972, 786, 756, 727; ^{1}H NMR (400 MHz, DMSO-d_6): δ 7.51 (tt, 4H, *J* = 7.8, 1.2), 7.45 (d, 8H, *J* = 7.8), 7.40 (t, 8H, *J* = 7.6), 7.30 (d, 4H, *J* = 7.6), 7.19 (t, 4H, *J* = 7.4), 7.10 (t, 2H, *J* = 7.6), 7.07 (s, 4H), 5.36 (s, 2H), 3.48 (t, 4H, *J* = 7.0), 1.87 (quin, 2H, *J* = 7.0); ^{13}C NMR (100 MHz, $CDCl_3$): δ 193.3, 146.2, 141.9, 139.0, 131.0, 128.4, 128.3, 128.1, 127.6, 126.2, 117.0, 51.4, 35.6, 30.8. Anal. calc. for $C_{53}H_{42}N_2O_4$ (770.9): C 82.57; H 5.49; N 3.63. Found: C 82.55; H 5.31; N 3.62.

3,5-Dibenzoyl-4-(naphthalen-1-yl)-1,4-dihydropyridine (**6a**). A mixture of each of 3-*N*,*N*-dimethylamino-1-phenylpropenone (0.39 g, 2.2 mmol), 1-naphthaldehyde (0.156 g, 1 mmol) and ammonium acetate (0.07 g, 1 mmol) in glacial acetic acid (10 mL) was heated under reflux for 8 h. The solvent was then removed in vacuo and the remaining

residue was purified by column chromatography to give 0.1 g (24%) of **6a** as yellow crystals mp 299–300 °C. MS: *m/z* = 415 (M^+); IR: 3241, 3156, 3043, 2957, 1628, 1597, 1573, 1494, 1469, 1369, 1220, 1116, 968, 778, 732, 715, 697, 645; ^{1}H NMR (400 MHz, $CDCl_3$): δ 8.91 (d, 1H, *J* = 8.8), 7.71 (d, 1H, *J* = 8.4), 7.65 (d, 1H, *J* = 8.4), 7.61–7.58 (m, 2H), 7.48–7.39 (m, 8H), 7.32 (t, 4H, *J* = 7.6), 6.94–6.93 (m, 2H), 6.53 (br, 1H), 6.37 (s, 1H); ^{13}C NMR (100 MHz, $CDCl_3$): δ 194.8, 139.0, 136.2, 133.5, 131.4, 131.0, 129.1, 128.5, 128.1, 128.0, 127.9, 127.4, 126.2, 125.6, 125.3, 125.1, 120.1, 32.3. HRMS = 415.1567 ($C_{29}H_{21}NO_2$ requires 415.1566).

Synthesis of dihydropyridines **6b**–**f** *and* **7a,b***: General procedure*

A mixture of each of the appropriate enaminones **1** (2.2 mmol), 1-naphthaldehyde or 9-phenanthrenecarboxaldehyde (1 mmol) and the arylamine (1 mmol) in glacial acetic acid (10 mL) was heated under reflux for 24 h. The solvent was then removed in vacuo and the remaining residue was crystallized from ethanol or purified by column chromatography to give the corresponding products **6b**–**f** and **7a,b**.

3,5-Dibenzoyl-4-(naphthalen-1-yl)-1-phenyl-1,4-dihydropyridine (**6b**). Yellow crystals, yield 0.17 g (34%), mp 259–260 °C. MS: *m/z* = 491 (M^+); IR: 3055, 3030, 1663, 1644, 1628, 1593, 1573, 1494, 1347, 1310, 1276, 1225, 1173, 1142, 1108, 760, 700, 654; ^{1}H NMR (400 MHz, $CDCl_3$): δ 8.96 (d, 1H, *J* = 8.8), 7.76 (d, 1H, *J* = 8.0), 7.61–7.65 (m, 3H), 7.54 (d, 4H, *J* = 8.4), 7.50–7.43 (m, 6H), 7.38 (s, 2H), 7.36–7.28 (m, 5H), 7.24 (d, 2H, *J* = 8.0), 6.50 (s, 1H); ^{13}C NMR (100 MHz, $CDCl_3$): δ 194.6, 143.3, 138.8, 138.2, 133.6, 131.4, 131.2, 130.2, 128.6, 128.2, 127.9, 127.6, 127.3, 126.8, 126.4, 125.7, 125.3, 125.0, 122.0, 121.1, 32.5. HRMS = 491.1879 ($C_{35}H_{25}NO_2$ requires 491.1879).

3,5-Dibenzoyl-1-(p-methoxyphenyl)-4-(naphthalen-1-yl)-1,4-dihydropyridine (**6c**). Yellow fibers, yield 0.19 g (36%), mp 134–135 °C. MS: *m/z* = 521 (M^+); IR: 3056, 2971, 2931, 1633, 1561, 1511, 1332, 1278, 1244, 1230, 1147, 1111, 784, 711; ^{1}H NMR (400 MHz, $CDCl_3$): δ 8.94 (d, 1H, *J* = 8.8), 7.74 (d, 1H, *J* = 8.0), 7.68–7.2 (m, 3H), 7.50 (d, 4H, *J* = 7.6), 7.46–7.39 (m, 4H), 7.33 (d, 4H, *J* = 7.6), 7.25 (s, 2H), 7.17 (d, 2H, *J* = 8.4), 6.93 (d, 2H, *J* = 8.4), 6.46 (s, 1H), 3.81 (s, 3H); ^{13}C NMR (100 MHz, $CDCl_3$): δ 194.6,

158.5, 139.0, 138.9, 136.7, 133.6, 131.5, 131.2, 128.7, 128.5, 128.2, 127.9, 127.6, 127.4, 126.4, 125.7, 125.4, 125.1, 123.2, 121.4, 115.2, 55.7, 32.4. HRMS = 521.1985 ($C_{36}H_{27}NO_3$ requires 521.1985).

3,5-Bis(p-methoxybenzoyl)-1-(p-methoxyphenyl)-4-(naphthalen-1-yl)-1,4-dihydropyridine (**6d**). Yellow crystals, yield 0.09 g (16%), mp 201–202 °C. MS: *m*/*z* = 581 (M^+); IR: 2930, 2905, 2835, 1661, 1630, 1598, 1572, 1509, 1460, 1339, 1311, 1260, 1227, 1166, 1145, 1110, 1066, 1023, 776, 603; ^1H NMR (400 MHz, $CDCl_3$): δ 8.88 (d, 1H, *J* = 8.8), 7.71 (d, 1H, *J* = 8.4), 7.66–7.62 (m, 3H), 7.50 (d, 4H, *J* = 8.8), 7.46–7.41 (m, 2H), 7.22 (s, 2H), 7.17 (d, 2H, *J* = 9.2), 6.93 (d, 2H, *J* = 9.2), 6.80 (d, 4H, *J* = 8.8), 6.47 (s, 1H), 3.80 (s, 3H), 3.78 (s, 6H); ^{13}C NMR (100 MHz, $CDCl_3$): δ 193.7, 162.1, 158.3, 137.9, 136.9, 133.6, 131.43, 131.4, 130.8, 127.9, 127.4 (two overlapped CH), 126.3, 125.6, 125.3, 125.0, 123.0, 121.1, 115.2, 113.4, 55.6, 55.3, 33.1. HRMS = 581.2196 ($C_{38}H_{31}NO_5$ requires 581.2196). Anal. Calcd for $C_{38}H_{31}NO_5$ (581.7): C 78.47; H 5.37; N 2.41. Found: C 78.22; H 4.70; N 3.07.

3,5-Bis(p-chlorobenzoyl)-1-(p-methoxyphenyl)-4-(naphthalen-1-yl)-1,4-dihydropyridine (**6e**). Yellow crystals, yield 0.06 g (11%), mp 192–193 °C. MS: *m*/*z* = 590 (M^+); IR: 3053, 2955, 2930, 1633, 1590, 1511, 1282, 1229, 1110, 1090, 908, 836, 788, 733; ^1H NMR (400 MHz, $CDCl_3$): δ 8.90 (d, 1H, *J* = 8.8), 7.77 (d, 1H, *J* = 8.0), 7.71–7.62 (m, 3H), 7.49 (d, 2H, *J* = 8.0), 7.45 (d, 4H, *J* = 8.4), 7.31 (d, 4H, *J* = 8.4), 7.21 (s, 2H), 7.18 (d, 2H, *J* = 8.8), 6.97 (d, 2H, *J* = 8.8), 6.42 (s, 1H), 3.84 (s, 3H); ^{13}C NMR (100 MHz, $CDCl_3$): δ 193.3, 158.7, 138.9, 137.4, 137.1, 136.4, 133.5, 131.3, 129.9, 128.7, 128.6, 128.5, 128.0, 127.7, 126.4, 125.8, 125.3, 124.8, 123.2, 121.2, 115.3, 55.7, 30.9. HRMS = 589.1206 ($C_{36}H_{25}Cl_2NO_3$ requires 589.1206).

3,5-Dibenzoyl-1-(p-chlorophenyl)-4-(naphthalen-1-yl)-1,4-dihydropyridine (**6f**). Yellow crystals, yield 0.11 g (21%), mp 278–280 °C. MS: *m*/*z* = 525 (M^+); IR: 3055, 2970, 1664, 1648, 1627, 1592, 1570, 1492, 1334, 1275, 1246, 1224, 1144, 1109, 779, 765, 702, 654; ^1H NMR (400 MHz, $CDCl_3$): δ 8.92 (d, 1H, *J* = 8.4), 7.75 (d, 1H, *J* = 8.0), 7.69 (d, 1H, *J* = 8.0), 7.66–7.63 (m, 2H), 7.52 (d, 4H, *J* = 8.0), 7.49–7.34 (m, 10H), 7.30 (s, 2H), 7.17 (d, 2H, *J* = 8.8), 6.48 (s, 1H); ^{13}C NMR (100 MHz, $CDCl_3$): δ 194.5, 141.8, 138.6, 137.6,

133.6, 132.4, 131.42, 130.3, 128.7, 128.5, 128.4, 128.3, 128.0, 127.7, 127.3, 126.5, 125.5, 125.3, 125.0, 122.4, 122.3, 32.5. HRMS = 525.1490 ($C_{35}H_{24}ClNO_2$ requires 525.1490).

3,5-Dibenzoyl-1-(p-methoxyphenyl)-4-(phenanthren-9-yl)-1,4-dihydropyridine (**7a**). Yellow crystals, yield 0.09 g (15%), mp 245–247 °C. MS: *m*/*z* = 571 (M^+); IR: 2972, 2932, 2884, 1660, 1637, 1597, 1574, 1513, 1465, 1442, 1379, 1312, 1280, 1247, 1228, 1163, 1143, 1108, 953, 723; ^{1}H NMR (400 MHz, $CDCl_3$): δ 9.06 (d, 1H, *J* = 8.0), 8.65 (t, 2H, *J* = 9.2), 7.85 (s, 1H), 7.84 (d, 1H, *J* = 7.2), 7.78 (t, 1H, *J* = 7.2), 7.65 (t, 1H, *J* = 8.4), 7.59–7.54 (m, 2H), 7.49 (d, 4H, *J* = 6.8), 7.40 (t, 2H, *J* = 7.6), 7.31 (s, 2H), 7.30 (t, 4H, *J* = 6.8), 7.20 (d, 2H, *J* = 9.2), 6.95 (d, 2H, *J* = 8.8), 6.47 (s, 1H), 3.82 (s, 3H); ^{13}C NMR (100 MHz, $CDCl_3$): δ 194.6, 158.6, 139.2, 138.8, 136.7, 132.0, 131.2, 130.7, 130.2, 128.7, 128.2, 128.1, 126.9, 126.4, 126.3, 126.2, 125.8, 123.2, 122.6, 122.5, 121.5, 115.3, 55.7, 32.2. HRMS = 571.2141 ($C_{40}H_{29}NO_3$ requires 571.2141).

3,5-Bis(p-methoxybenzoyl)-1-(p-methoxyphenyl)-4-(phenanthren-9-yl)-1,4-dihydropyridine (**7b**). Yellow crystals, yield 0.08 g (13%), mp 138–140 °C. MS: *m*/*z* = 631 (M^+); IR: ^{1}H NMR (400 MHz, $CDCl_3$): δ 9.02 (d, 1H, *J* = 8.8), 8.65 (t, 2H, *J* = 8.8), 7.88 (s, 1H), 7.86 (d, 1H, *J* = 7.2), 7.77 (t, 1H, *J* = 8.8), 7.64 (t, 1H, *J* = 7.6), 7.62–7.57 (m, 2H), 7.53 (d, 4H, *J* = 8.8), 7.30 (s, 2H), 7.24 (d, 2H, *J* = 8.8), 6.99 (d, 2H, *J* = 8.8), 6.82 (d, 4H, *J* = 8.8), 6.50 (s, 1H), 3.85 (s, 3H), 3.79 (s, 6H); ^{13}C NMR (100 MHz, $CDCl_3$): δ 193.7, 162.2, 158.4, 138.2, 136.9, 132.0, 131.4, 130.9, 130.7, 130.1, 128.1, 126.9, 126.3, 126.2, 126.1, 125.7, 123.0, 122.6, 122.5, 121.2, 115.2, 113.5, 55.7, 55.3, 31.9. HRMS = 631.2352 ($C_{42}H_{33}NO_5$ requires 631.2353).

4-Phenyl-1,4-dihydropyridine-3,5-dicarboxaldehyde (**10a**). Yellow needles from ethanol, yield 73% (method A), mp 244–246 °C. MS: *m*/*z* = 213 (M^+, 50%), 136 (100%) ; IR: 3088, 3029, 2965, 1657, 1597, 1463, 1387, 1265, 1151, 1078, 941, 702 ; ^{1}H NMR (400 MHz, DMSO-d_6): δ 10.08 (s, 1H), 9.25 (s, 2H), 7.45 (s, 2H), 7.21 (t, 2H, *J* = 7.8), 7.14 (d, 2H, *J* = 7.8), 7.10 (t, 1H, *J* = 7.6), 4.75 (s, 1H); ^{13}C NMR (100 MHz, DMSO-d_6): δ 189.3, 145.7, 144.3, 127.9, 127.6, 126.1, 119.9, 32.9. Anal. calc. for $C_{13}H_{11}NO_2$ (213.2): C 73.23; H 5.20; N 6.57. Found: C 73.15; H 5.21; N 6.50.

1,4-Diphenyl-1,4-dihydropyridine-3,5-dicarboxaldehyde (**10b**). Yellow needles from ethanol, yield 72% (method A), 86% (method B), mp 176–178 °C. MS: *m*/*z* = 289 (M^+, 30%), 212 (100%), 154 (10%); IR: 3067, 3027, 2954, 1662, 1577, 1494, 1349, 1201, 1143, 1067, 761, 704; ^{1}H NMR (400 MHz, DMSO-d_6): δ 9.41 (s, 2H), 8.06 (s, 2H), 7.65 (d, 2H, *J* = 7.6), 7.57 (t, 2H, *J* = 8.0), 7.41 (t, 1H, *J* = 7.8), 7.24 (m, 4H), 7.14 (m, 1H), 4.80 (s, 1H); ^{13}C NMR (100 MHz, DMSO-d_6): δ 189.9, 145.2, 144.5, 142.0, 129.8, 128.1, 127.8, 126.8, 126.4, 122.4, 120.8, 32.7. Anal. calc. for $C_{19}H_{15}NO_2$ (289.3): C 78.87; H 5.23; N 4.84. Found: C 78.75; H 5.21; N 4.80.

Diethyl 4-phenyl-1,4-dihydropyridine-3,5-dicarboxylate (**12**) [4]. Colorless oil, yield 65% (method A). MS: *m*/*z* = 301 (M^+, 30%), 224 (80%), 168 (50%); IR: 3329, 3103, 2981, 1699, 1603, 1492, 1372, 1292, 1189, 1076, 754, 708; ^{1}H NMR (400 MHz, $CDCl_3$): δ 7.35 (dd, 2H *J* = 7.8, 1.4), 7.31 (d, 2H, *J* = 5.2), 7.23 (t, 2H, *J* = 7.8), 7.15 (tt, 1H, *J* = 7.8, 1.4), 6.52 (t, 1H, *J* = 5.4), 4.89 (s, 1H), 4.12–4.02 (m, 4H), 1.19 (t, 6H, *J* = 6.8); ^{13}C NMR (100 MHz, $CDCl_3$): δ 167.0, 146.8, 133.4, 128.4, 128.0, 126.4, 108.8, 60.1, 37.6, 14.2. Anal. calc. for $C_{17}H_{19}NO_4$ (301.35): C 67.76; H 6.36; N 4.65. Found: C 67.75; H 6.31; N 4.60.

4-Phenyl-1,4-dihydropyridine-3,5-dicarbonitrile (**14**). Yellow needles from ethanol, yield 76% (method A), mp 176–178 °C. MS: *m*/*z* = 207 (M^+, 50%), 130 (100%); IR: 3228, 3106, 3001, 2206, 1670, 1609, 1510, 1452, 1246, 1074, 756, 700; ^{1}H NMR (400 MHz, $CDCl_3$): δ 7.43 (t, 2H, *J* = 7.8), 7.35 (t, 1H, *J* = 7.6), 7.31 (d, 2H, *J* = 7.6), 4.49 (s, 1H); ^{13}C NMR (150 MHz, DMSO-d_6): δ 143.6, 137.6, 128.9, 127.8, 127.7, 119.3, 85.4, 40.0. Anal. calc. for $C_{13}H_9N_3$ (207.2): C 75.35; H 4.38; N 20.28. Found: C 75.25; H 4.31; N 20.30.

Oxidation of 1,4-dihydropyridines **2a–c** *and* **6a***: General procedure*

To a stirred cold (0–5 °C) solution of nitric acid (6 mL, 70%), the appropriate dihydropyridine derivatives **2a–c** and **6a** (1 mmol) was added portionwise within 5 min as solids. After stirring for an additional 15 min under cooling, the reaction mixture was kept at room temperature for 30 min, then poured onto crushed ice (ca. 10 g) and treated

with saturated solution of Na_2CO_3 to achieve a pH 8. The product was then extracted with $CHCl_3$ (3 × 50 mL) and dried over anhydrous sodium sulfate. The solvent was then removed in vacuo and the crude product was purified by crystallization from ethanol to give **15a**–**d**.

3,5-Dibenzoyl-4-phenylpyridine (**15a**). Colorless needles from ethanol, mp 190–192 °C, yield 0.28 g (76%). MS: *m*/*z* = 363 (M^+, 100%), 258 (25%), 105 (100%); IR: 3057, 3028, 1663, 1594, 1573, 1447, 1318, 1276, 1228, 1180, 1013, 916, 816, 706; ^{1}H NMR (400 MHz, $CDCl_3$): δ 8.86 (s, 2H), 7.59 (dd, 4H, *J* = 8.0, 1.6), 7.44 (tt, 2H, *J* = 7.6, 1.6), 7.29 (t, 4H, *J* = 7.8), 7.05 (m, 2H), 6.99 (m, 3H); ^{13}C NMR (100 MHz, $CDCl_3$): δ 195.8, 149.8, 146.8, 136.6, 135.4, 134.8, 133.7, 129.7, 129.3, 128.8, 128.4, 128.3; Anal. calc.for $C_{25}H_{17}NO_2$ (363.4): C 82.63; H 4.72; N 3.85. Found: C 82.54; H 4.69; N 3.90.

4-(p-Chlorophenyl)-3,5-dibenzoylpyridine (**15b**). Colorless needles from ethanol, mp 180–182 °C, yield 0.28 g (70%). MS: *m*/*z* = 399 (M + 2, 25%), 397 (M^+, 100%), 292 (20%), 105 (100%); IR: 3059, 2969, 1658, 1595, 1493, 1447, 1289, 1092, 1006, 840; ^{1}H NMR (400 MHz, DMSO-d_6): δ 8.89 (s, 2H), 7.68 (dd, 4H, *J* = 7.8, 1.2), 7.58 (t, 2H, *J* = 7.6), 7.42 (t, 4H, *J* = 7.6), 7.15 (d, 2H, *J* = 8.4), 7.02 (d, 2H, *J* = 8.4); ^{13}C NMR (100 MHz, DMSO-d_6): δ 195.1, 149.6, 144.6, 136.2, 134.9, 134.0, 133.8, 133.4, 130.7, 129.7, 128.7, 128.1. Anal. calc.for $C_{25}H_{16}ClNO_2$ (397.9): C 75.47; H 4.05; N 3.52. Found: C 75.38; H 4.09; N 3.47.

3,5-Dibenzoyl-4-p-tolylpyridine (**15c**). Colorless needles from ethyl acetate, mp 150–152 °C, yield 0.27 g (72%); IR: 3156, 2995, 1655, 1626, 1568, 1485, 1370, 1203, 1176, 1137, 1000, 836; LCMS: *m*/*z* = 378 (M + 1); MS: *m*/*z* = 377 (M^+, 100%), 275 (40%), 105 (85%); ^{1}H NMR (400 MHz, $CDCl_3$): δ 8.84 (s, 2H), 7.64 (d, 4H, *J* = 7.2), 7.48 (t, 2H, *J* = 7.4), 7.32 (t, 4H, *J* = 7.8), 6.96 (d, 2H, *J* = 8.4), 6.81 (d, 2H, *J* = 8.4), 2.10 (s, 3H); ^{13}C NMR (100 MHz, $CDCl_3$): δ 196.0, 149.9, 146.7, 138.8, 136.6, 135.4, 133.7, 131.9, 129.8, 129.1, 129.0, 128.4, 21.0. Anal. calc.for $C_{26}H_{19}NO_2$ (377.5): C 82.74; H 5.07; N 3.71. Found: C 82.68; H 5.00; N 3.67.

3,5-Dibenzoyl-4-(naphthalen-1-yl)pyridine (**15d**). Yellow needles, yield 0.33 g (80%), mp 181–182 °C. MS: *m*/*z* = 413 (M^+). IR: 3059, 3004, 1658, 1593, 1569, 1539, 1449, 1316, 1298, 1278, 1205, 1174, 1010, 903, 806, 782, 712; ^1H NMR (400 MHz, $CDCl_3$): δ 9.02 (s, 2H), 7.55 (tt, 2H, *J* = 7.2, 2.4), 7.44 (d, 1H, *J* = 8.0), 7.42–7.40 (m, 4H), 7.38–7.34 (m, 2H), 7.30–7.25 (m, 2H), 7.16–7.13 (m, 2H), 7.11–7.07 (m, 4H); ^{13}C NMR (100 MHz, $CDCl_3$): δ 195.6, 150.4, 145.9, 136.5, 136.3, 133.1, 132.8, 132.2, 130.6, 129.4, 129.1, 128.7, 128.1, 127.9, 126.6, 126.0, 125.4, 124.4. HRMS = 413.1409 ($C_{29}H_{19}NO_2$ requires 413.1410).

References

1. Elassar, A.-Z. A.; El-Khair, A. A. *Tetrahedron* **2003,** *59,* 8463–8480. doi:10.1016/S0040-4020(03)01201-8
2. Riyadh, S. M.; Abdelhamid, I. A.; Al-Matar, H. M.; Hilmy, N. M.; Elnagdi, M. H. *Heterocycles* **2008,** *75,* 1849–1905. doi:10.3987/REV-07-625
3. Böhme, H., Willinger, G. *Arch. Pharm.* 1969, 302, 974–985. doi:10.1002/ardp.19693021215
4. Mai, A.; Valente, S.; Meade, S.; Carafa, V.; Tardugno, M.; Nebbioso, A.; Galmozzi, A.; Mitro, N.; De Fabiani, E.; Altucci, L.; Kazantsev, A. *J. Med. Chem.* **2009,** *52,* 5496–5504. doi:10.1021/jm9008289

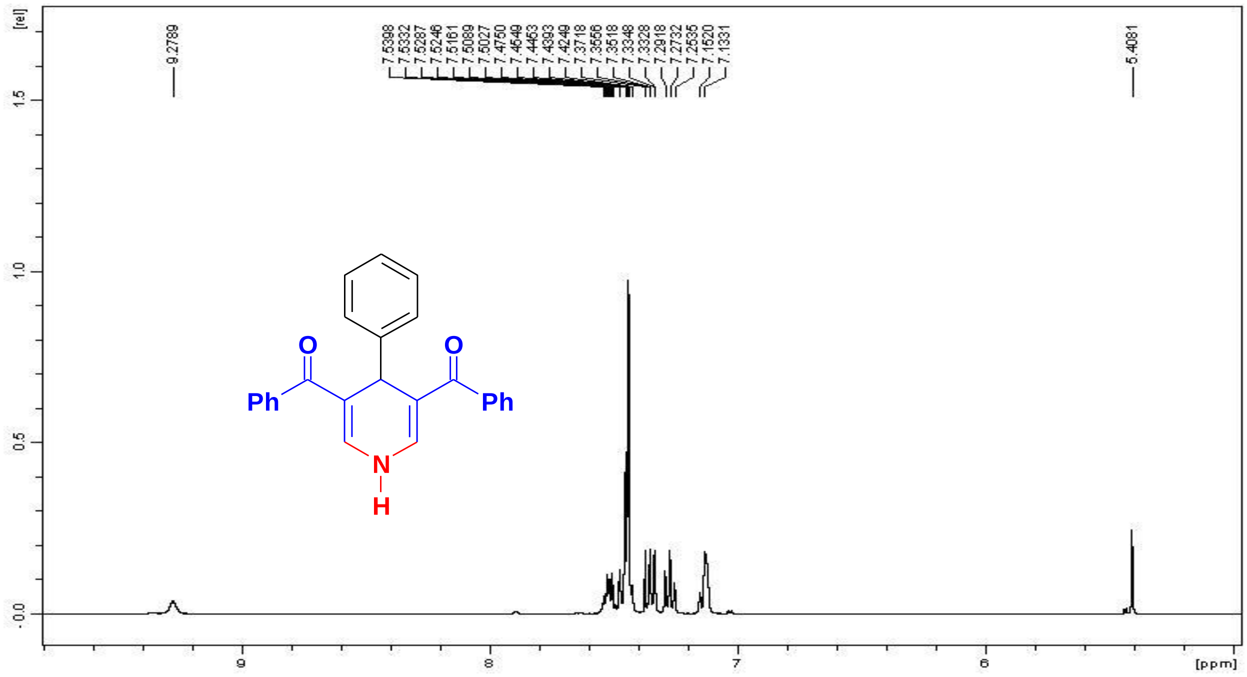

Compound **2a**

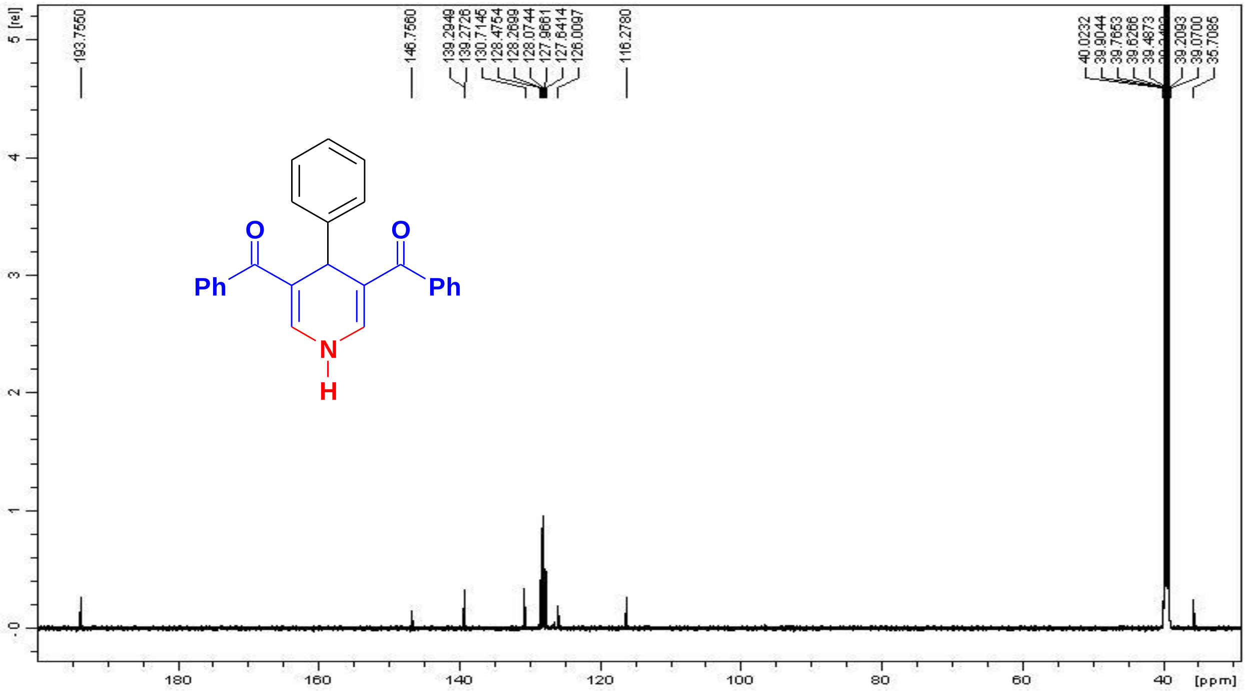

Compound **2a**

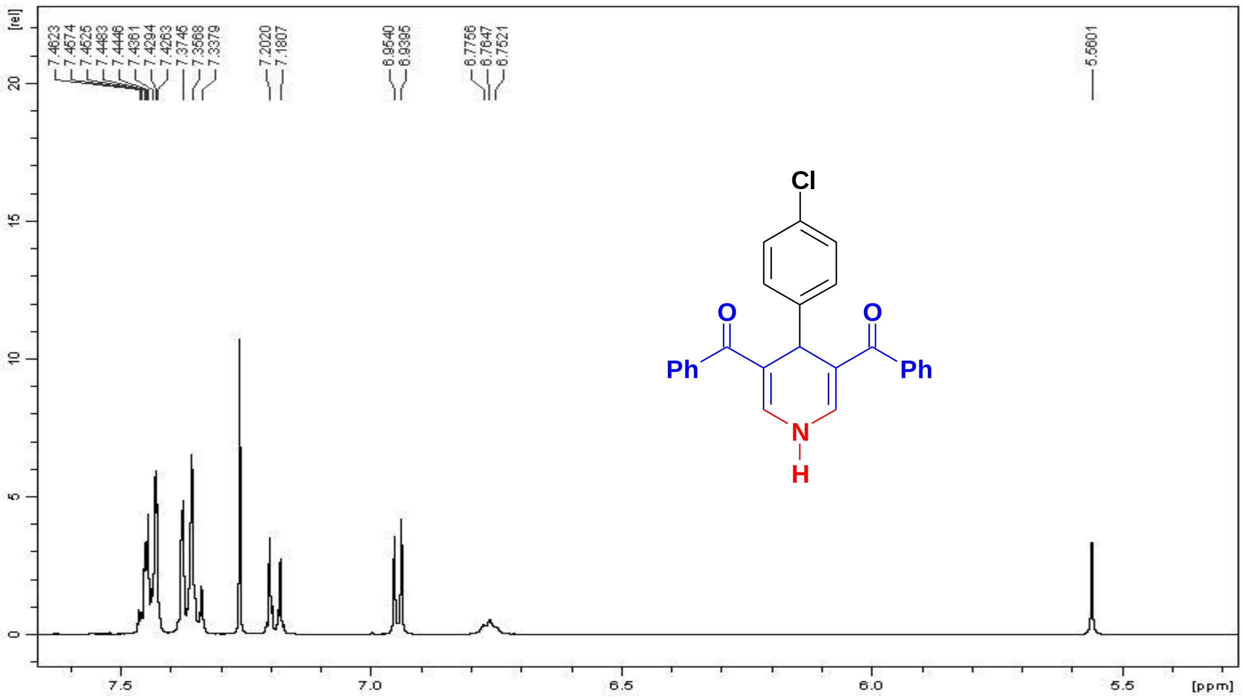

Compound **2b**

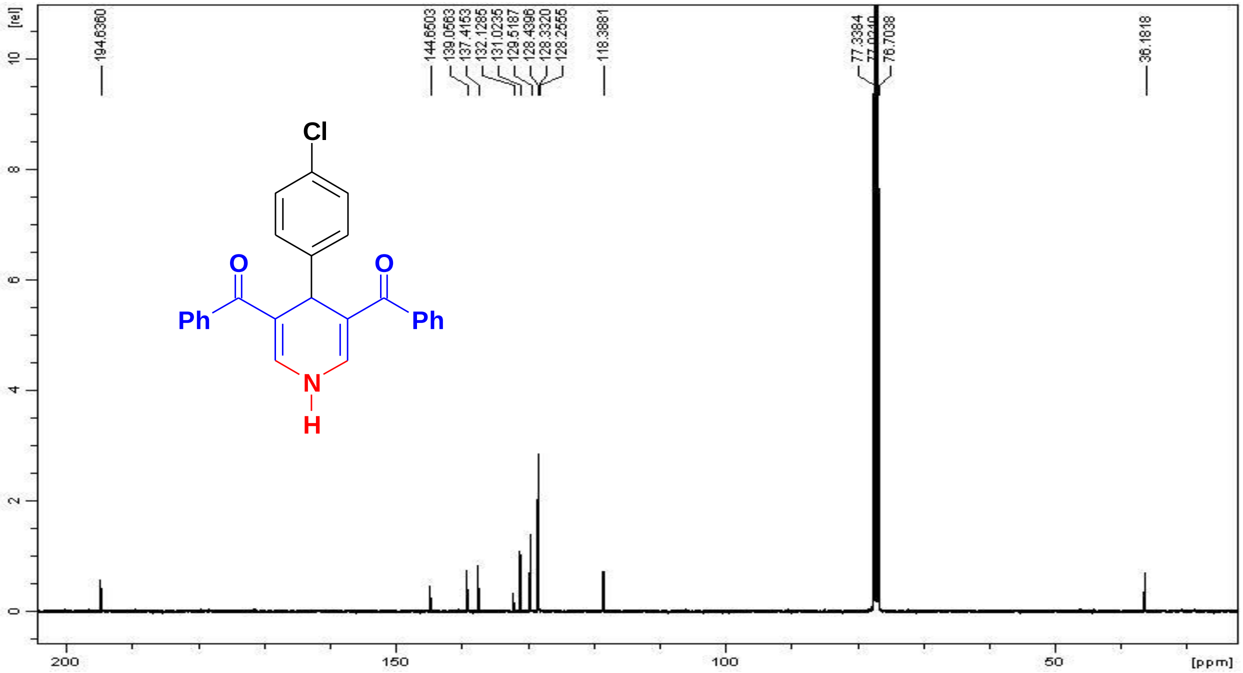

Compound **2b**

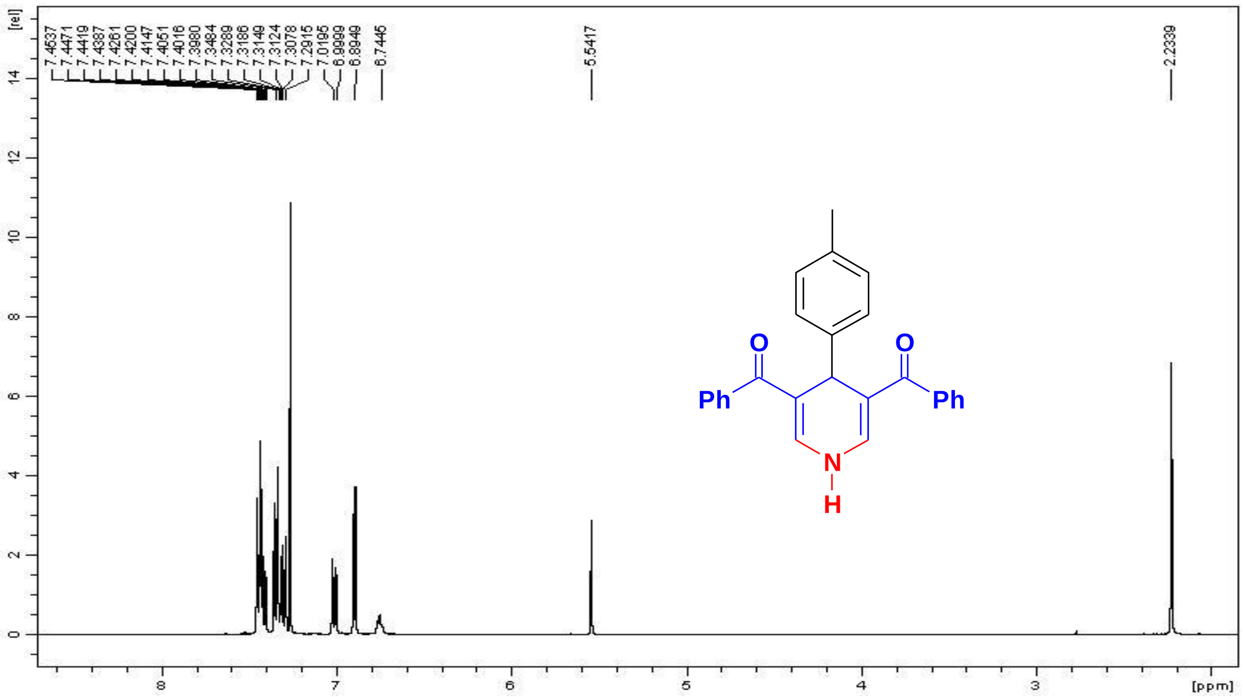

Compound **2c**

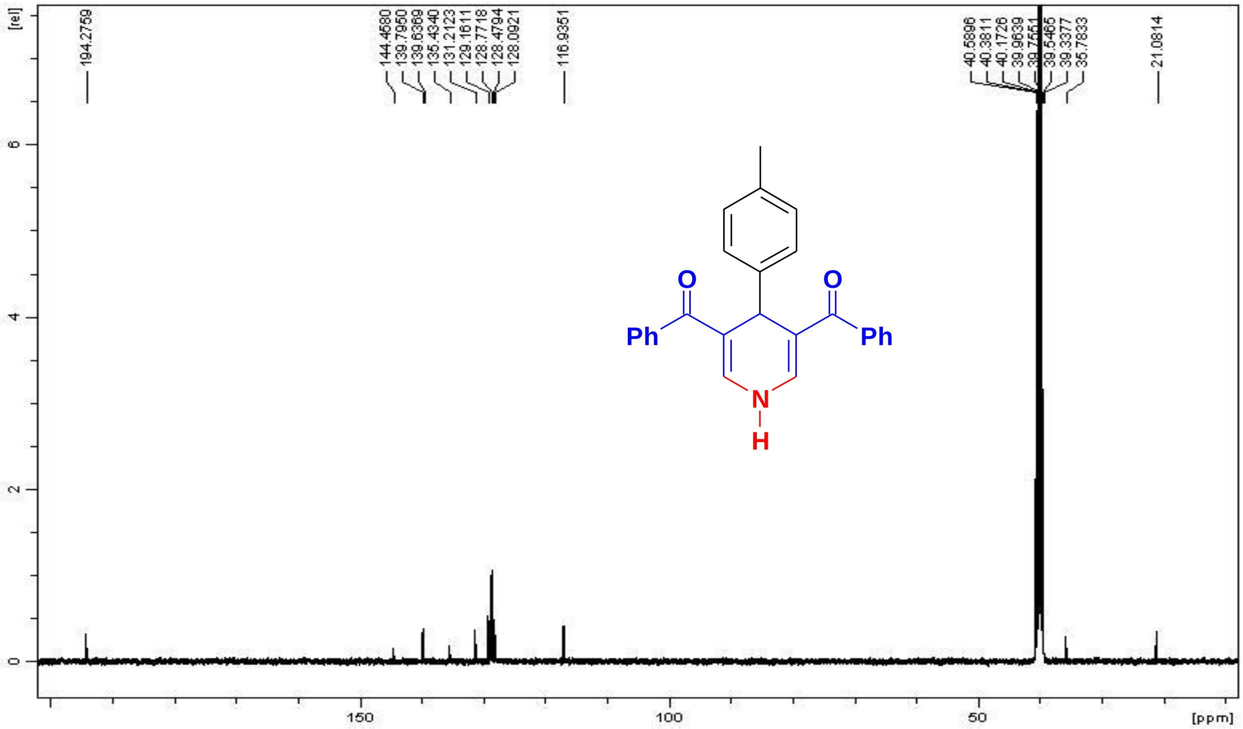

Compound **2c**

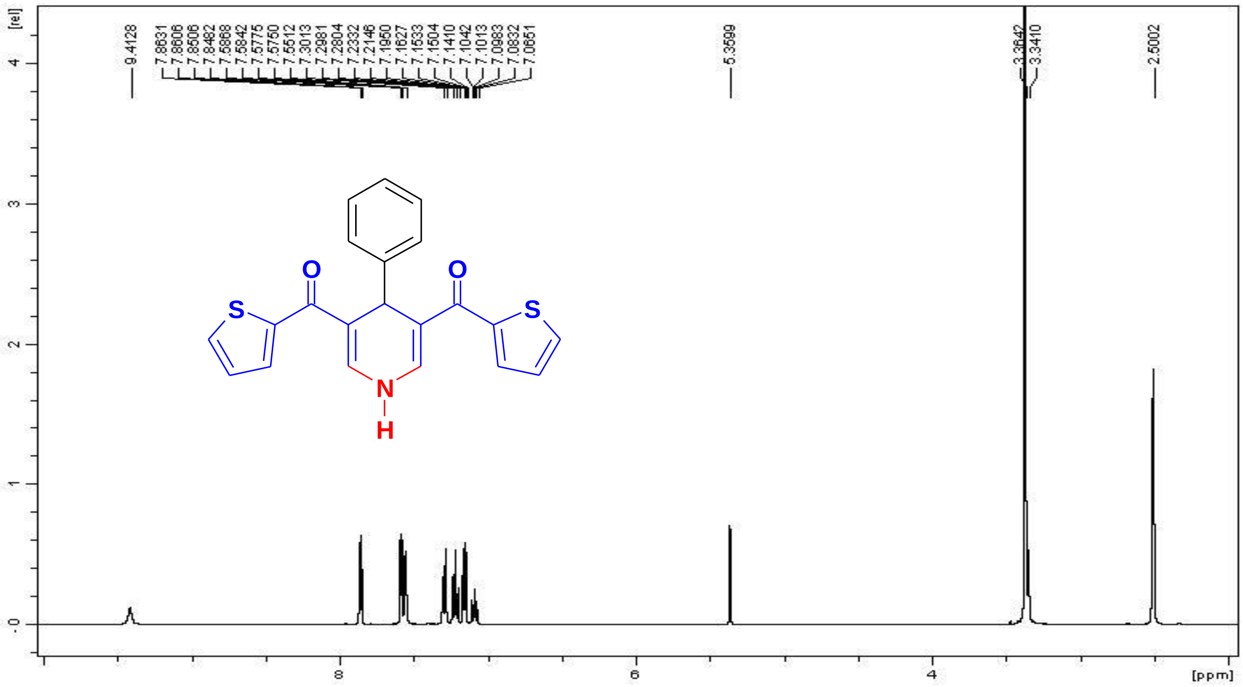

Compound **2d**

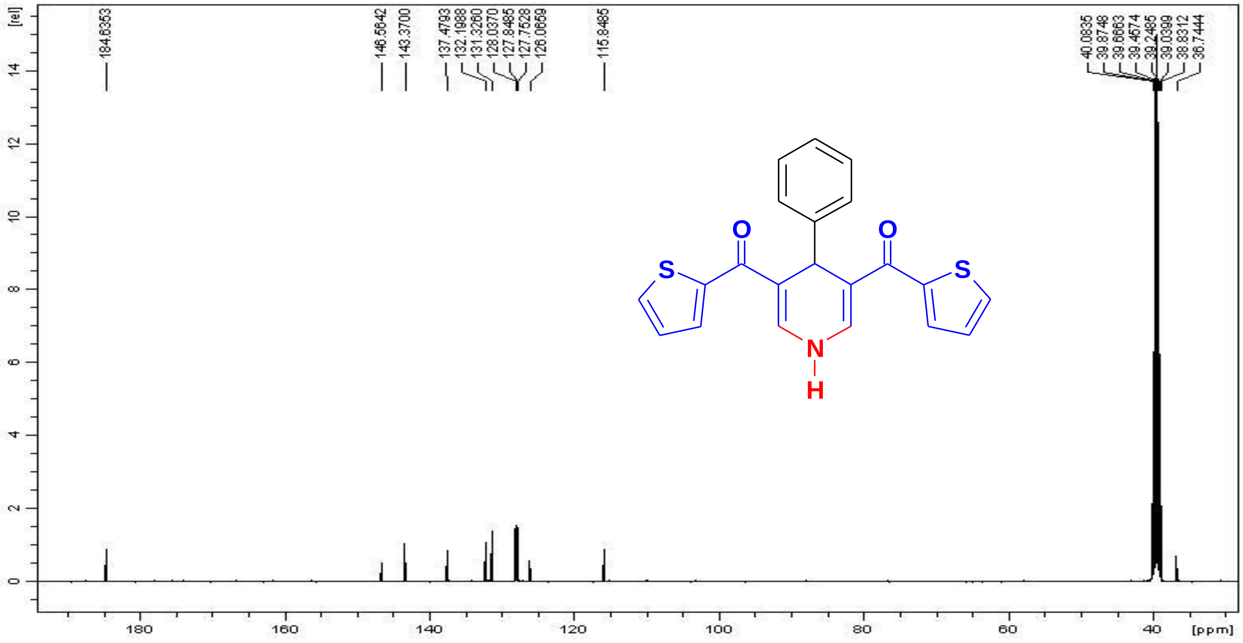

Compound **2d**

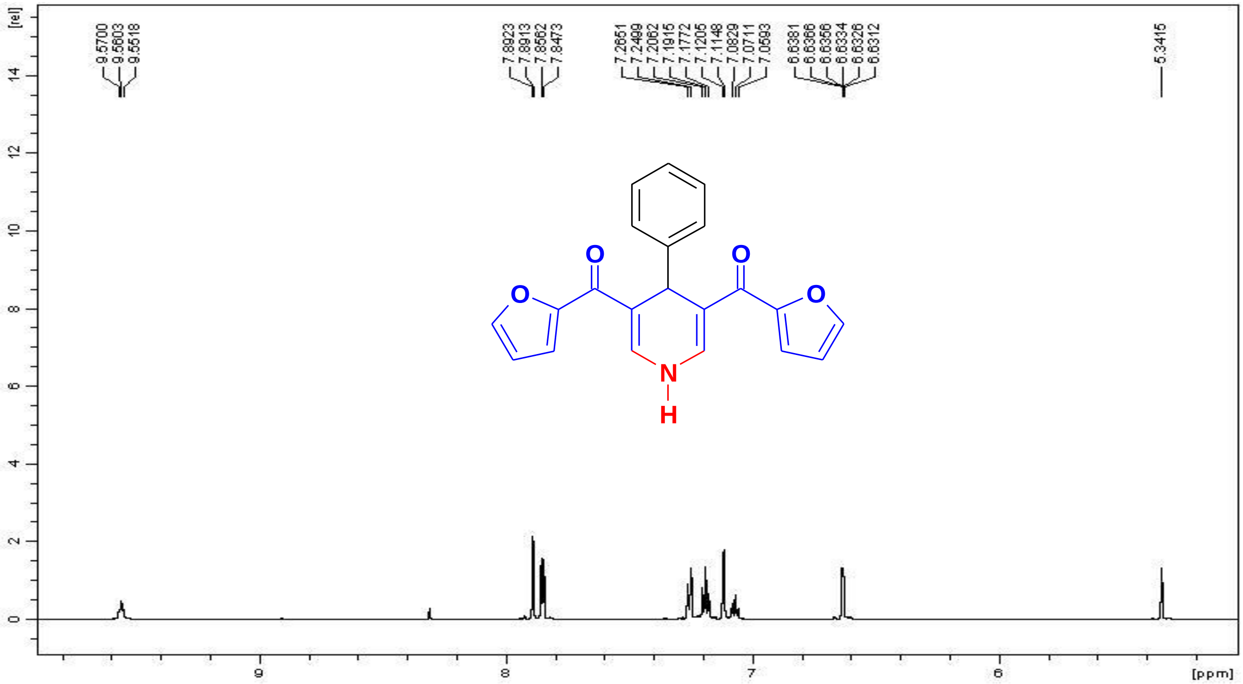

Compound **2e**

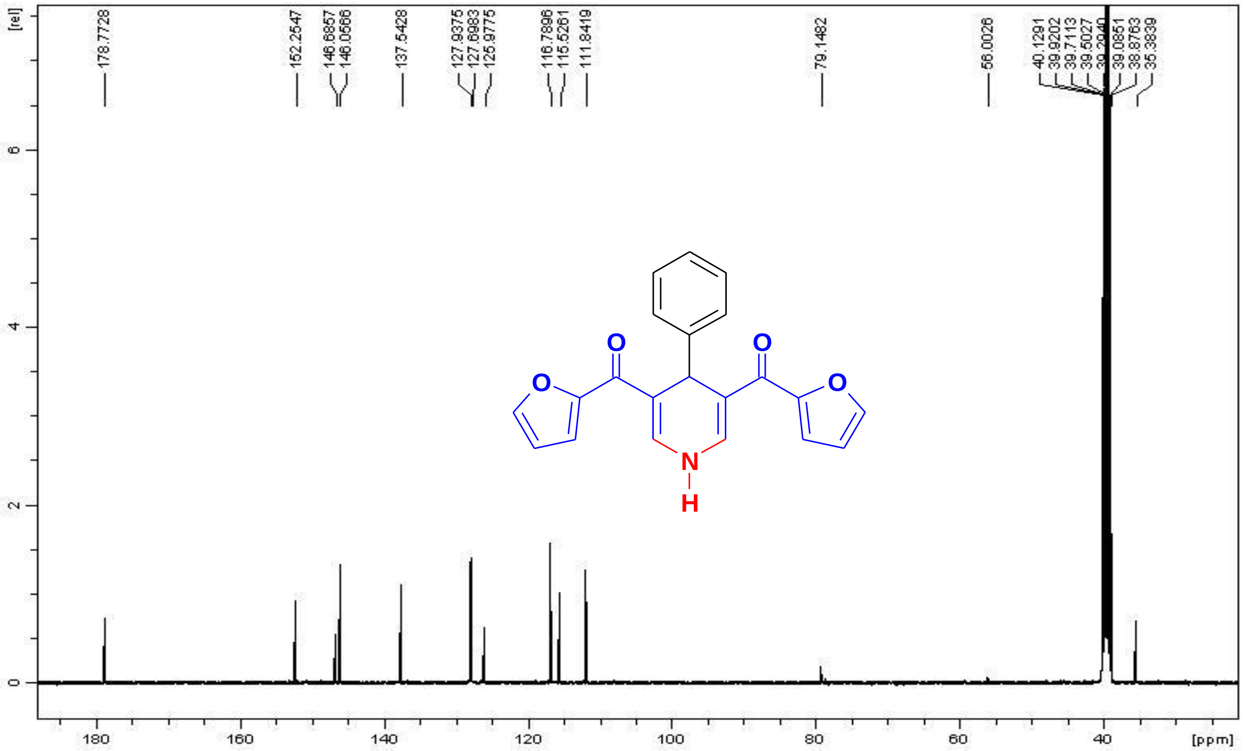

Compound **2e**

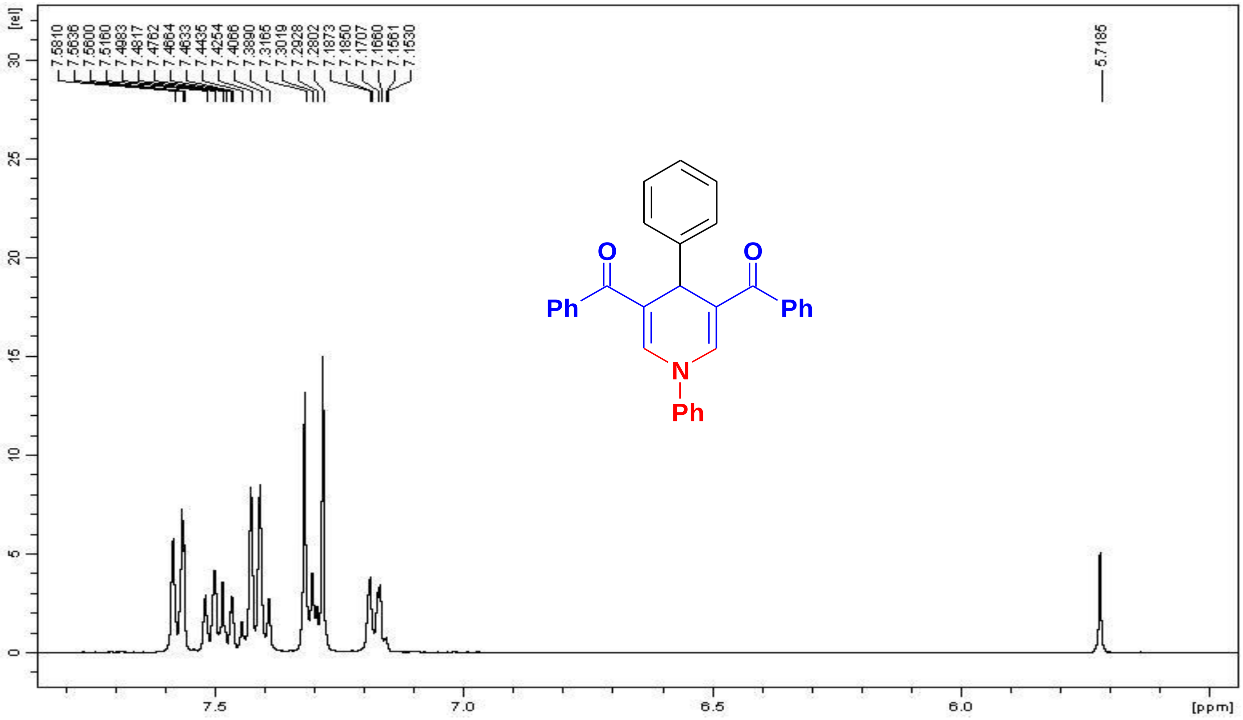

Compound **2f**

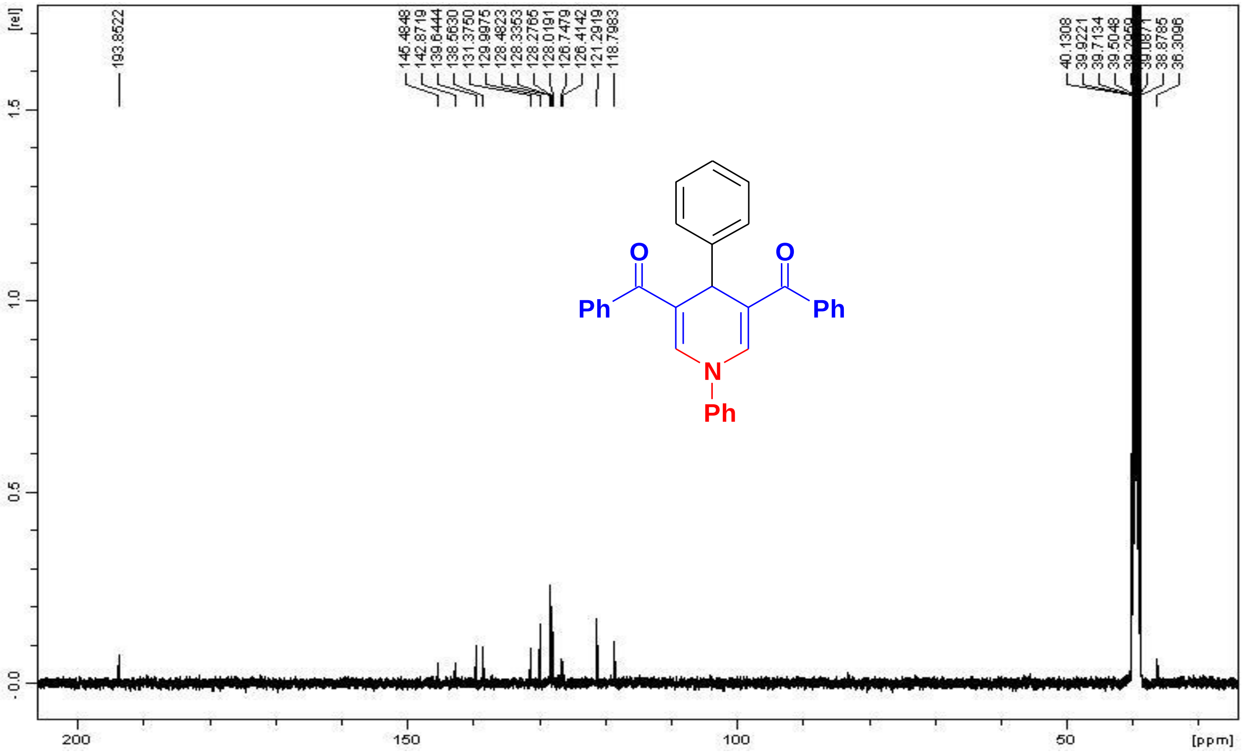

Compound **2f**

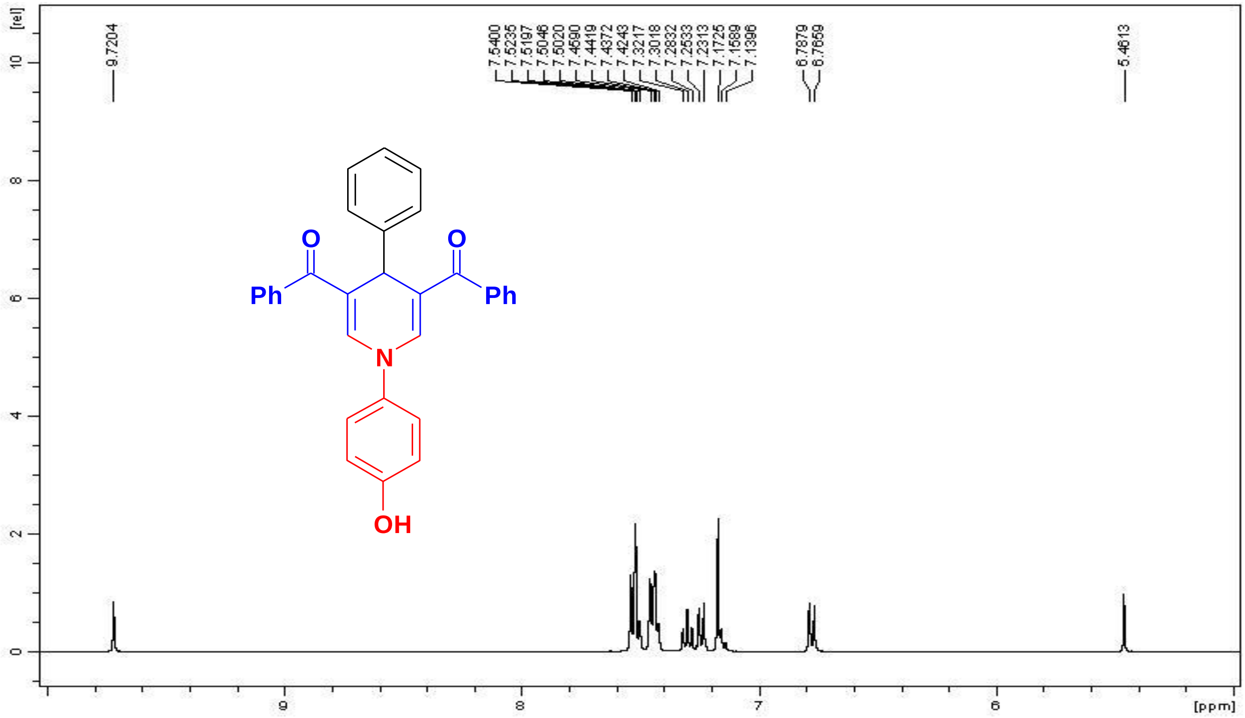

Compound **2g**

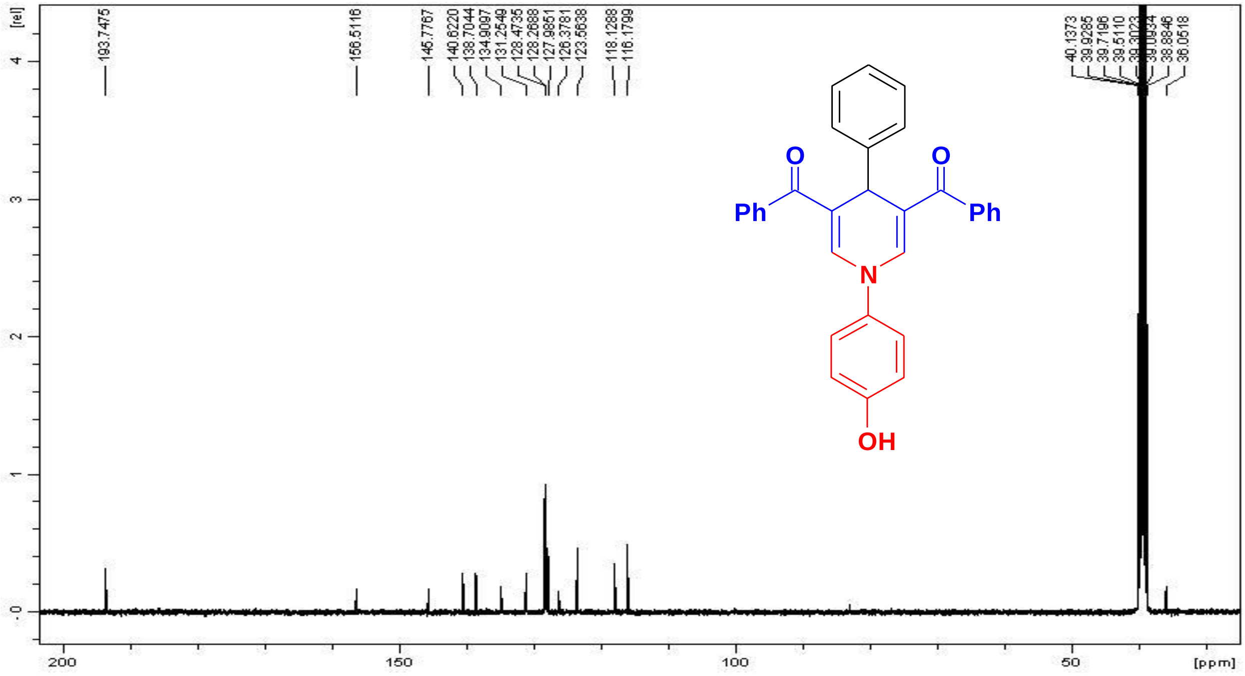

Compound **2g**

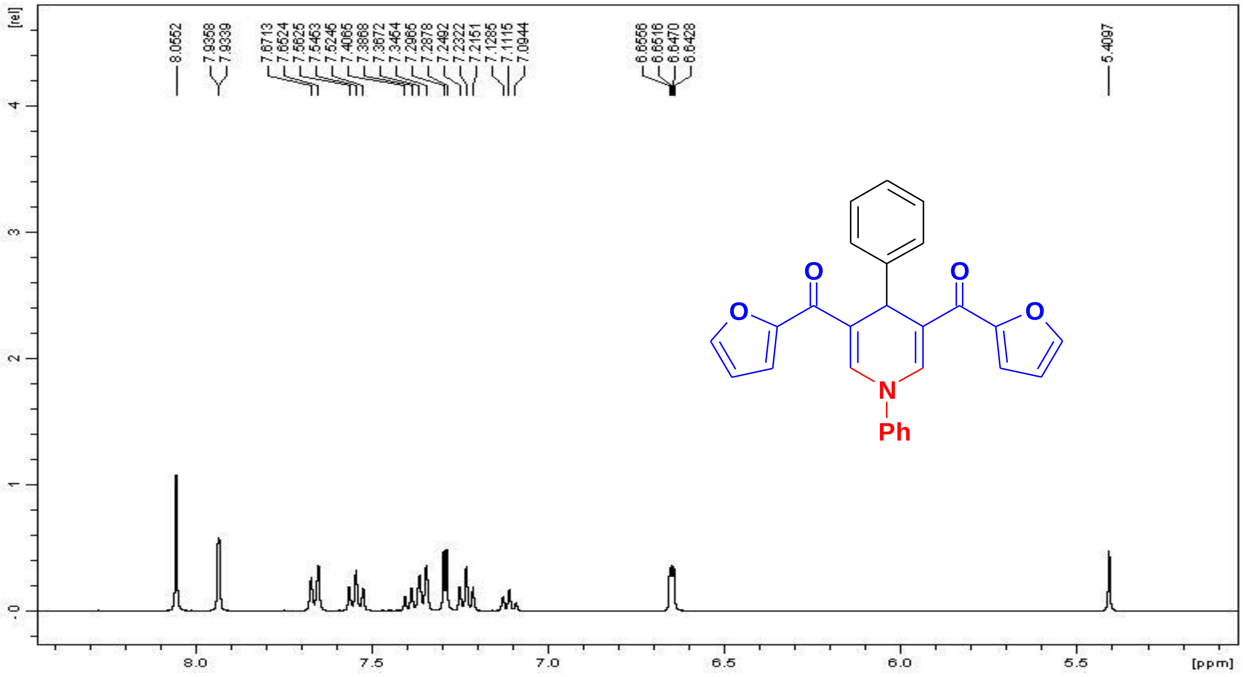

Compound **2h**

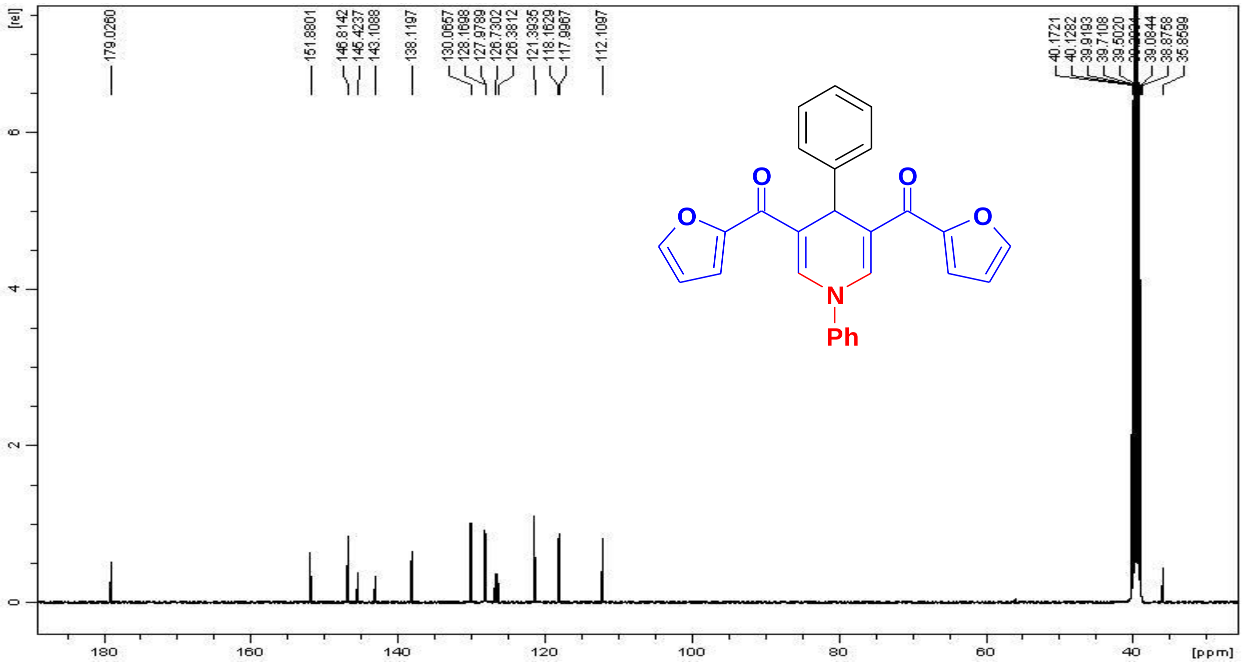

Compound **2h**

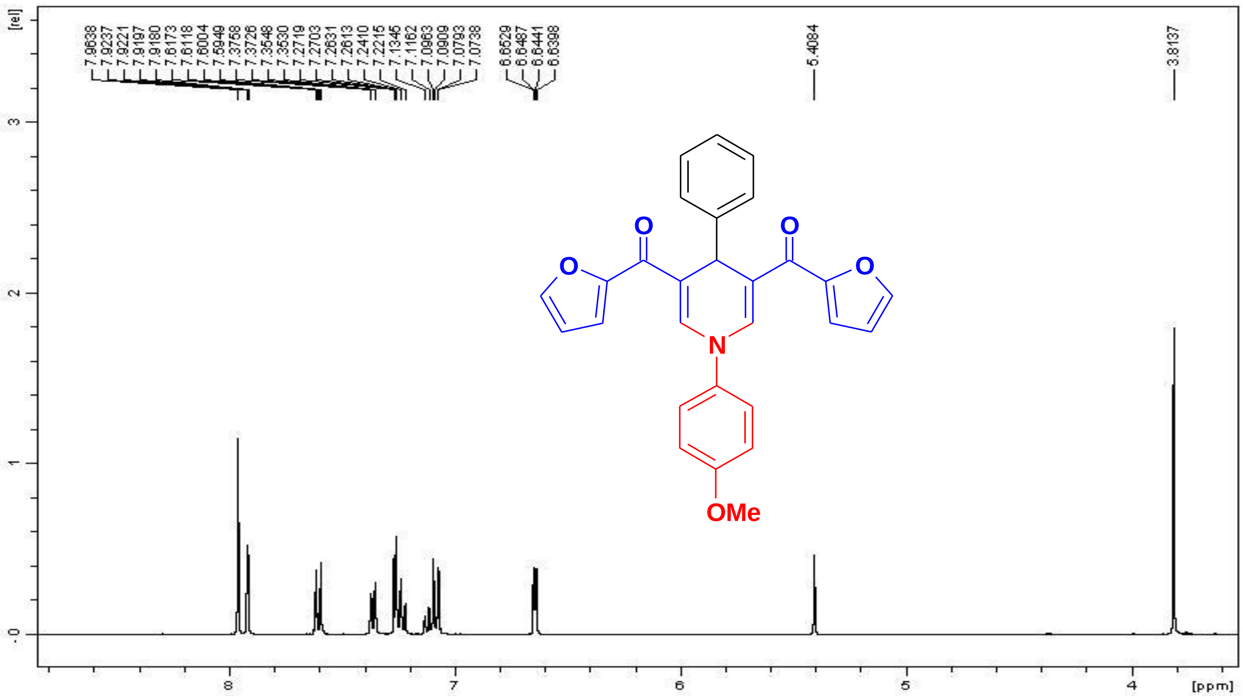

Compound **2i**

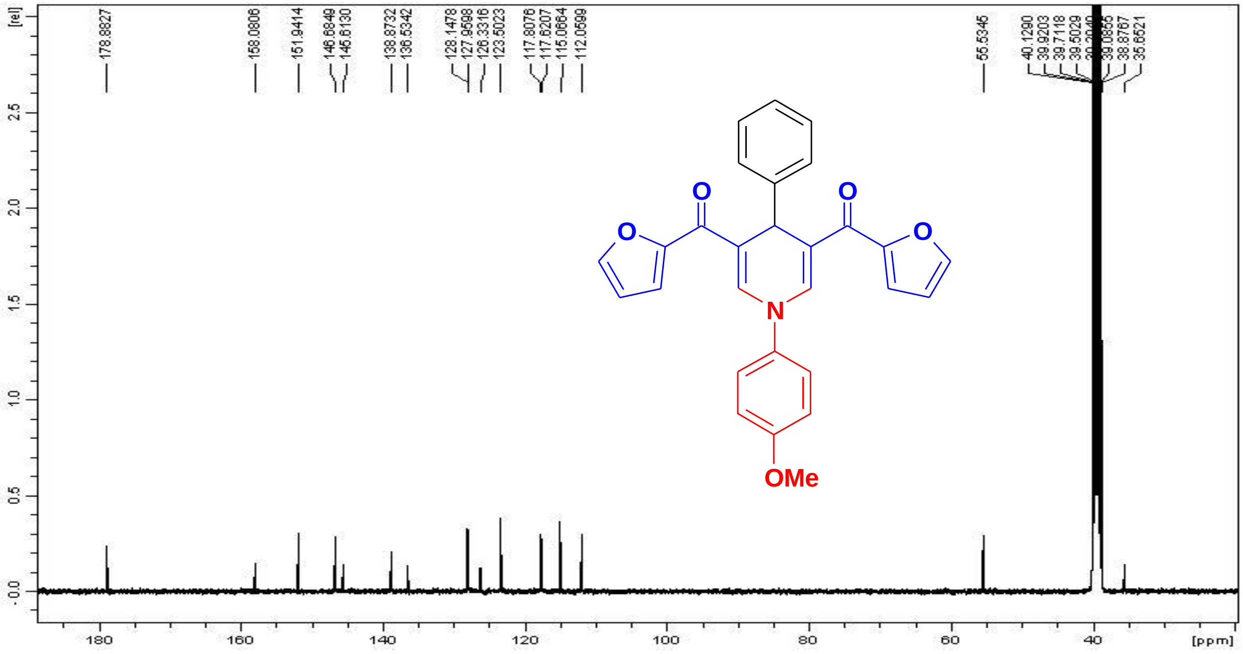

Compound **2i**

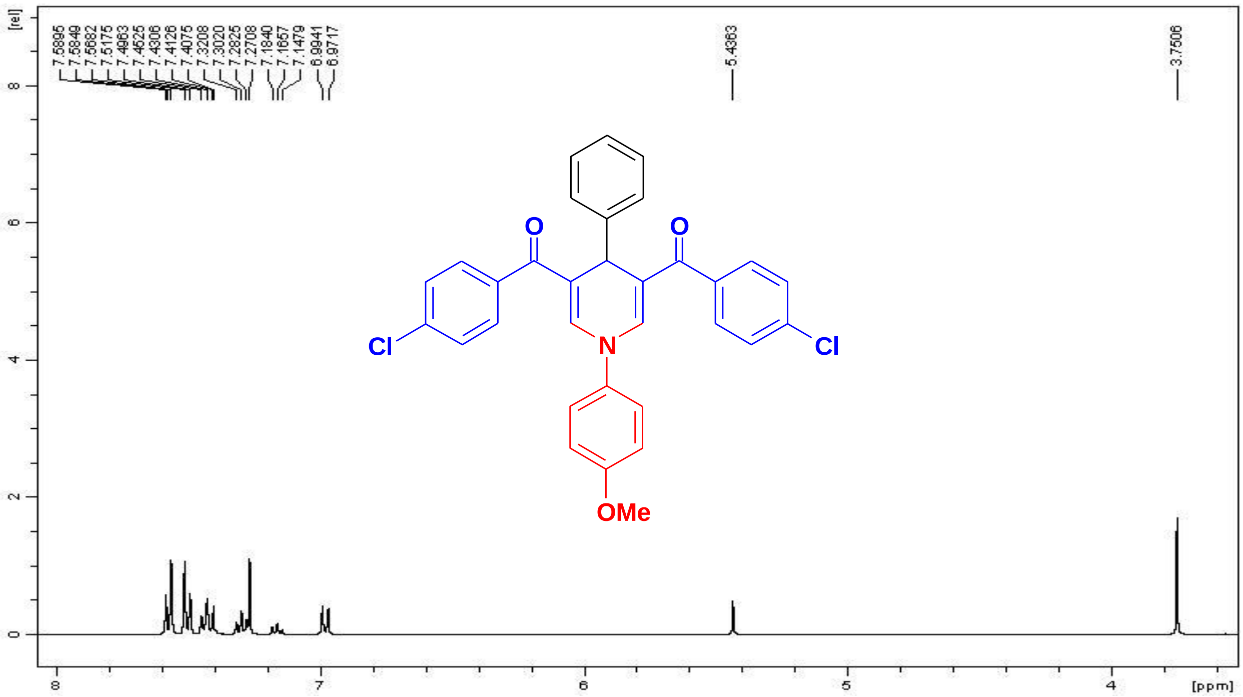

Compound **2j**

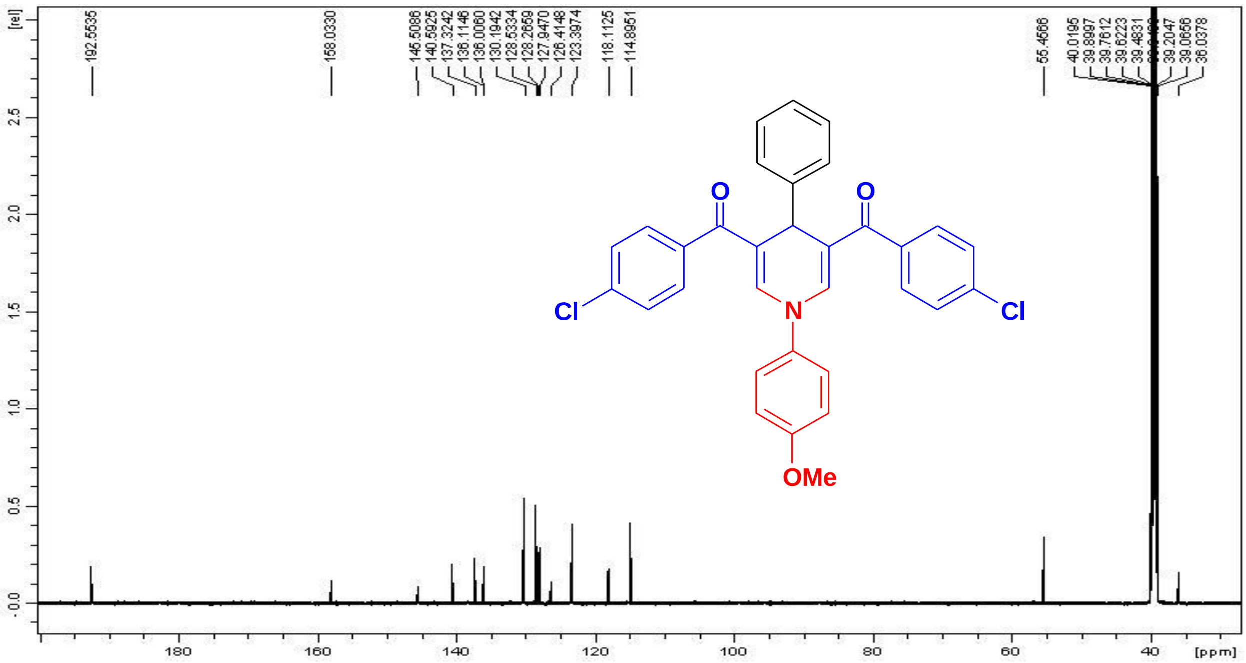

Compound **2j**

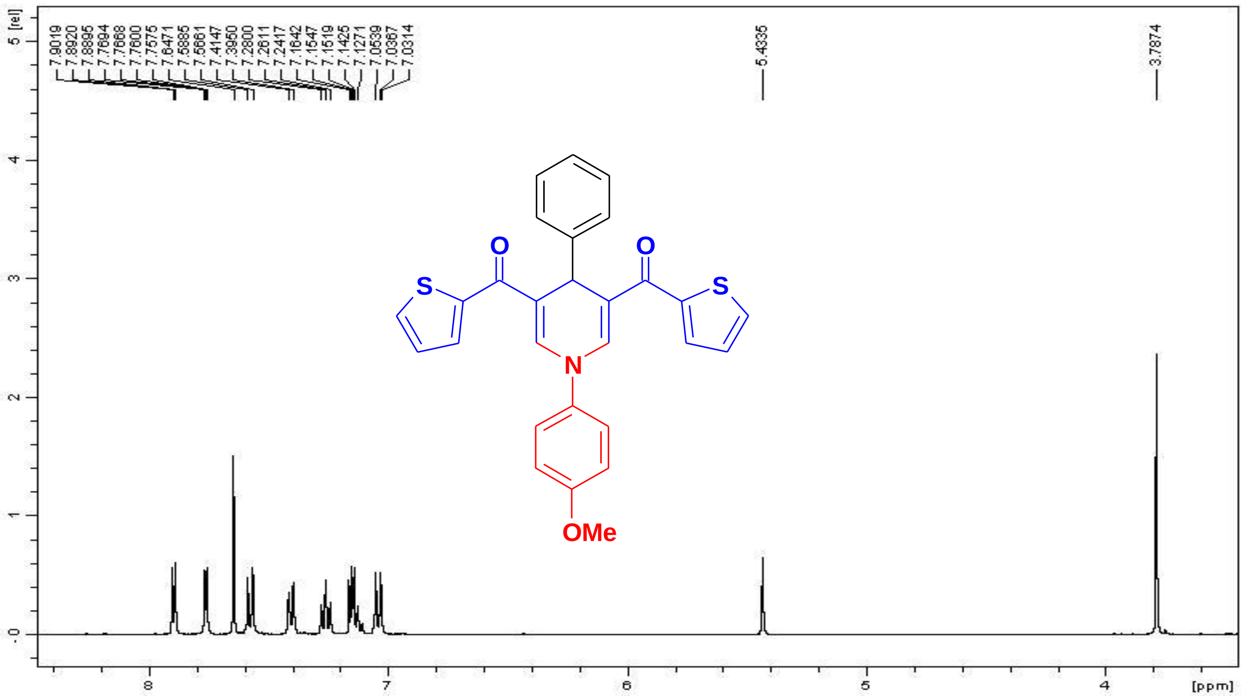

Compound **2k**

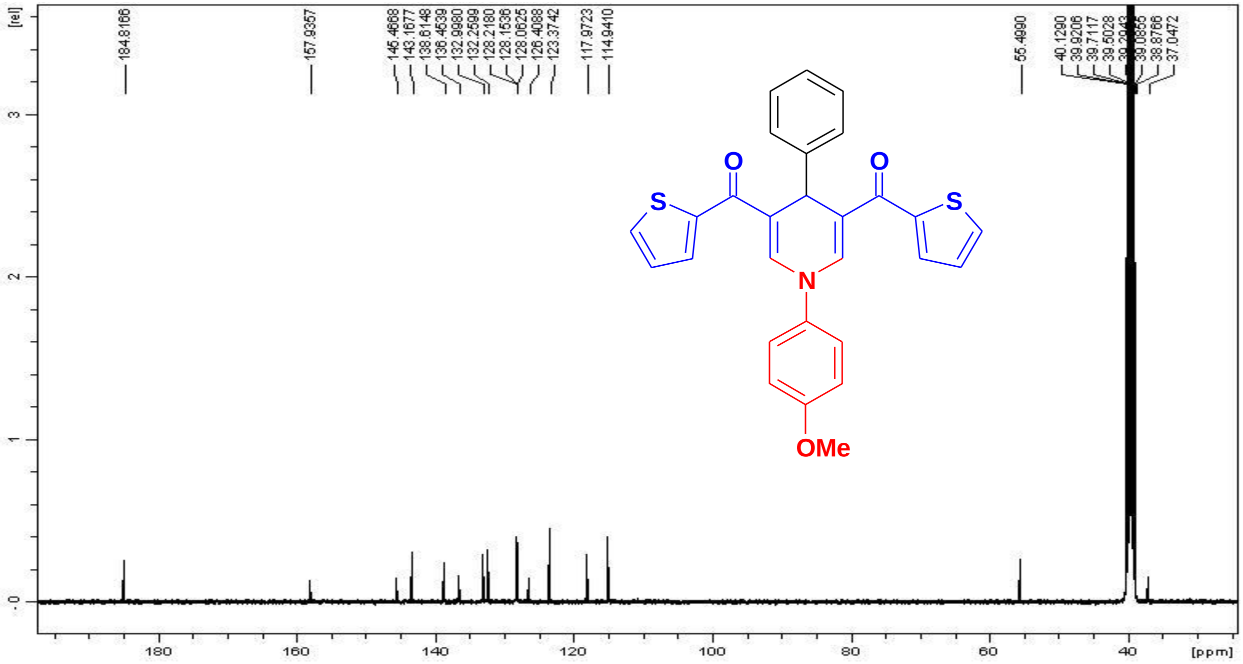

Compound **2k**

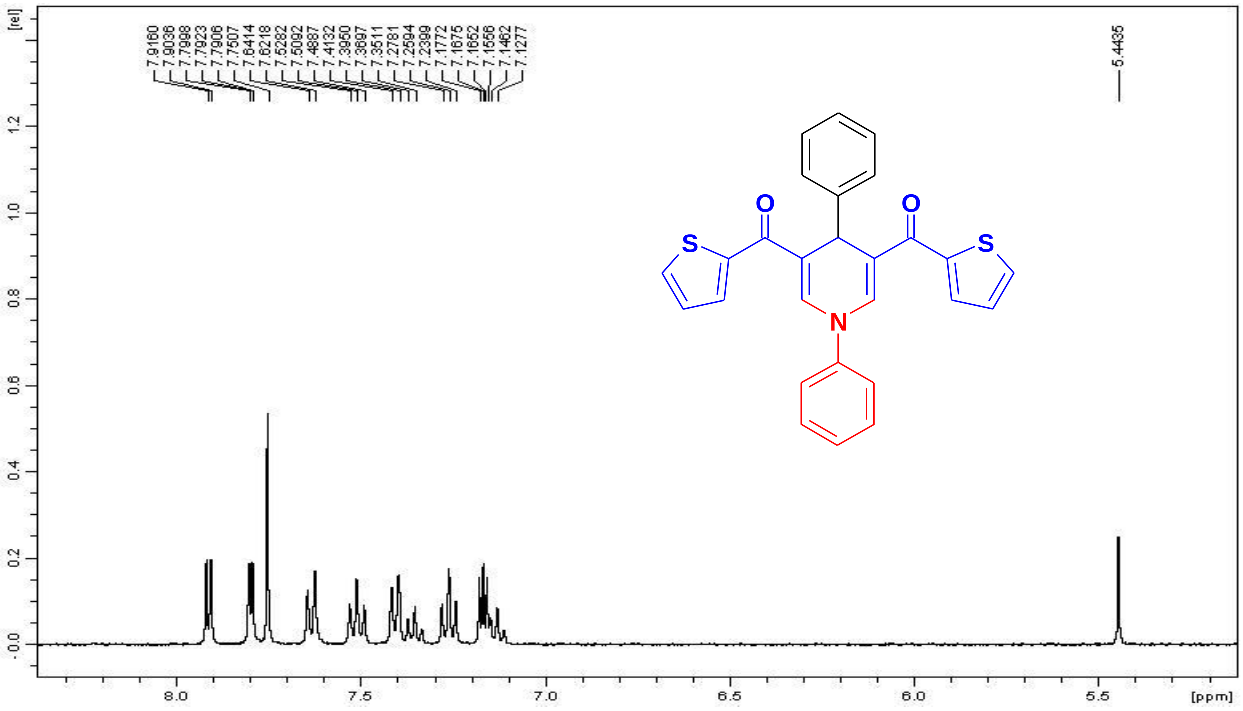

Compound **2l**

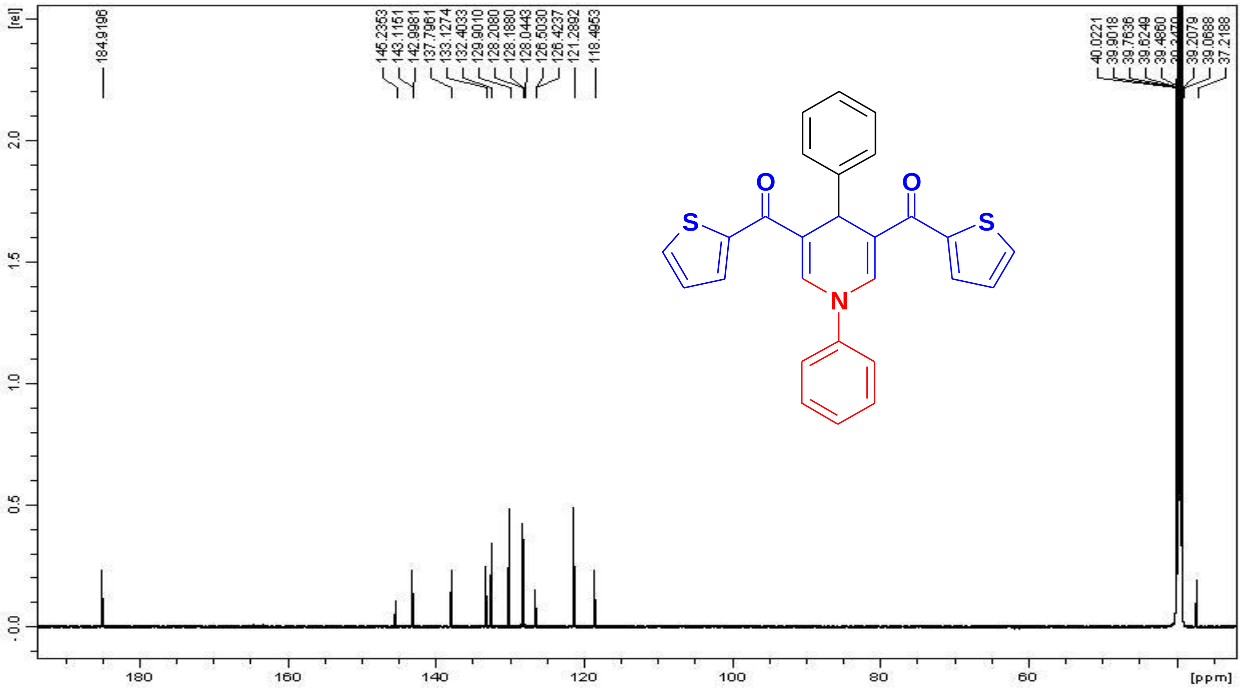

Compound **2l**

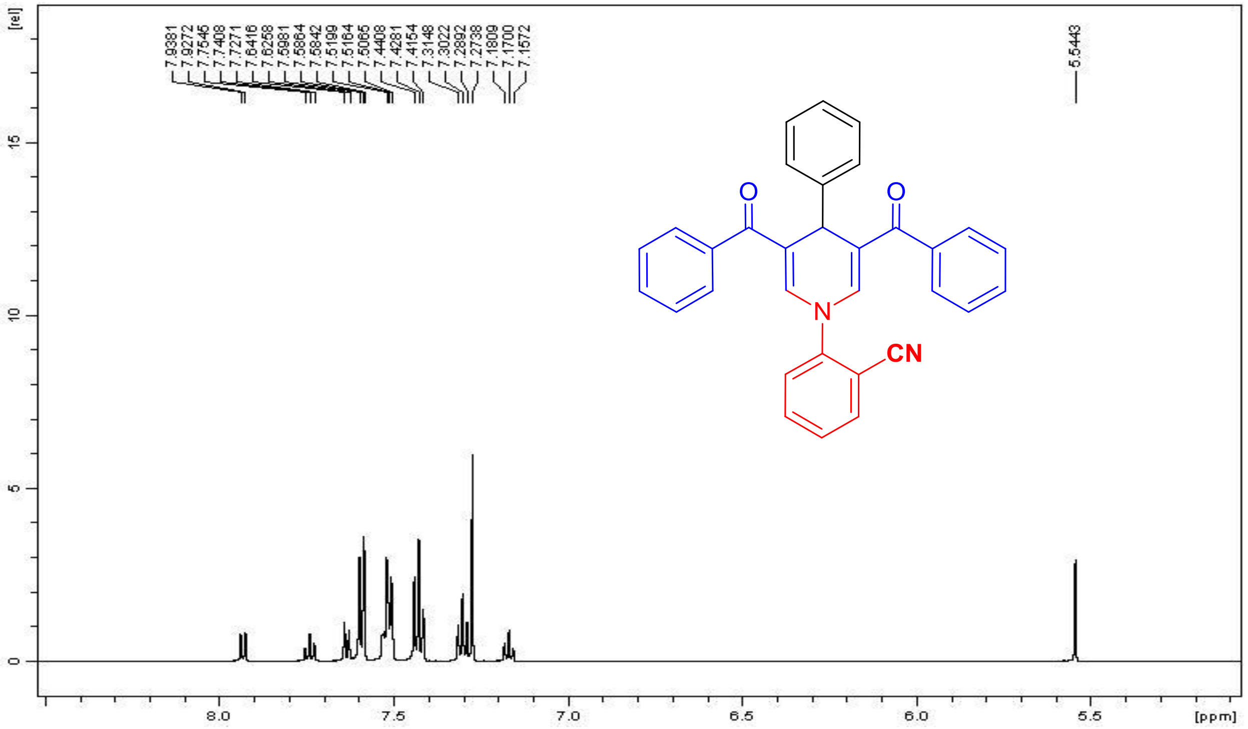

Compound **2m**

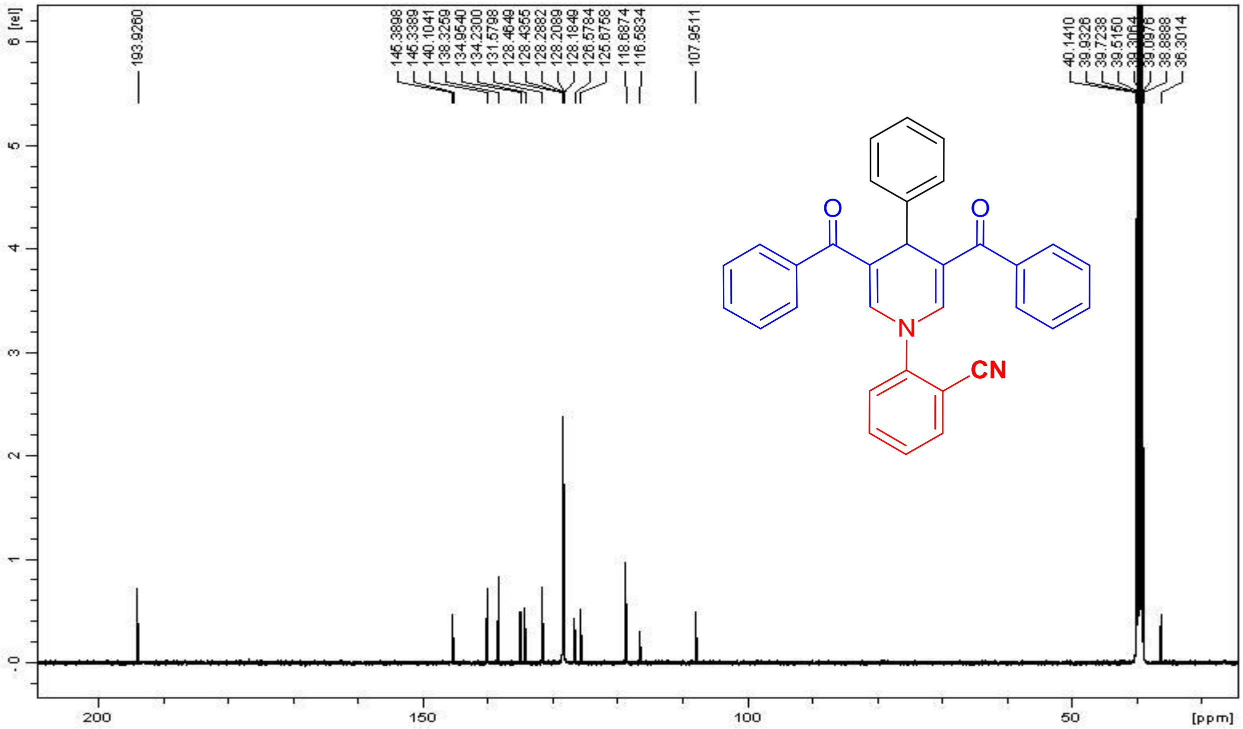

Compound **2m**

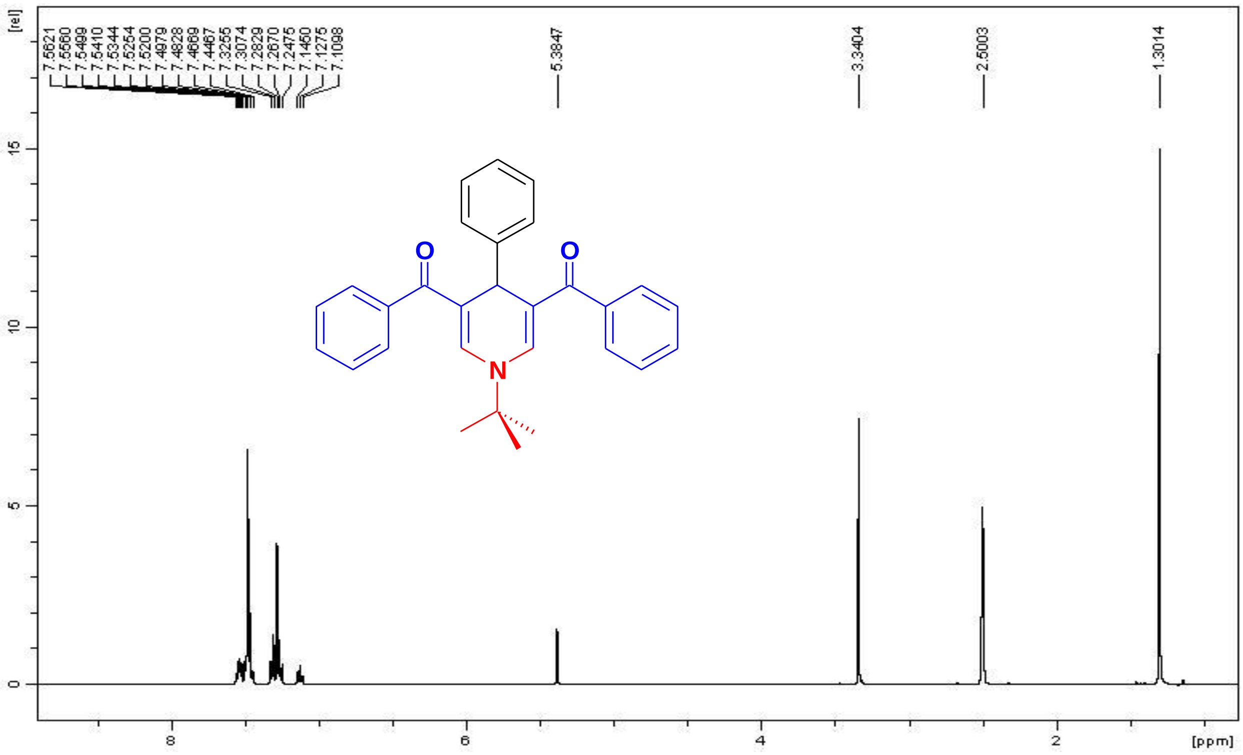

Compound **2n**

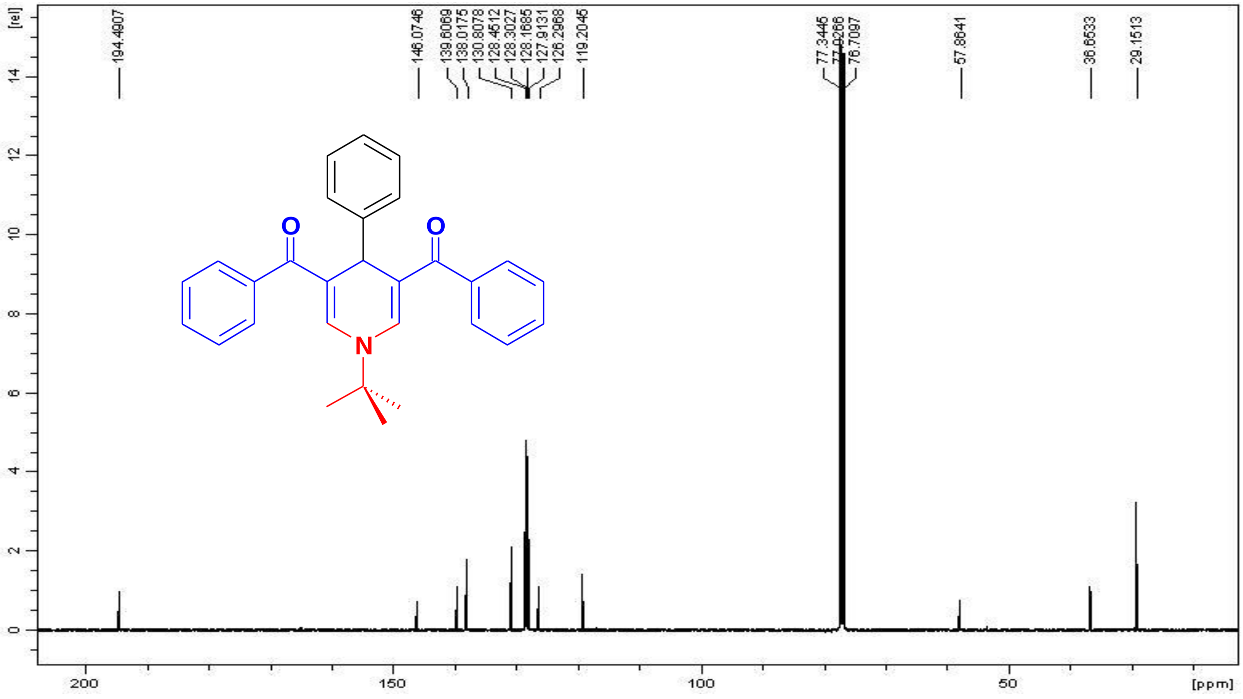

Compound **2n**

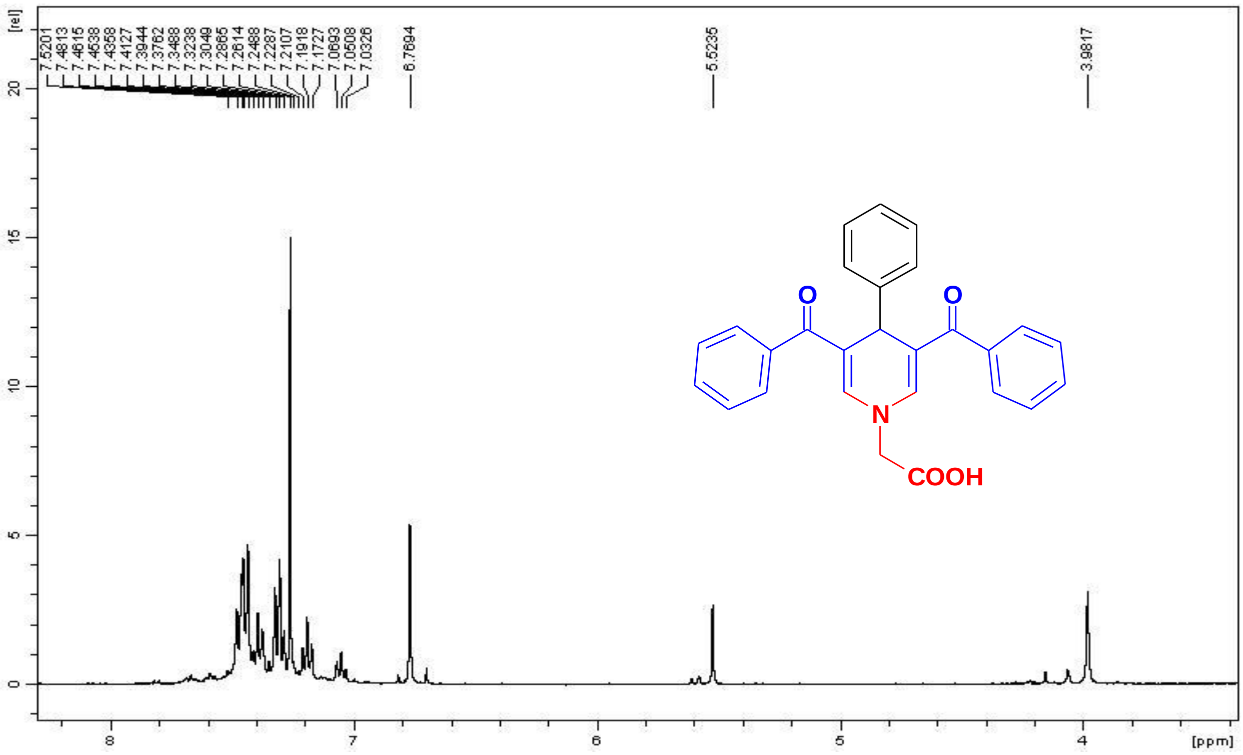

Compound **2o**

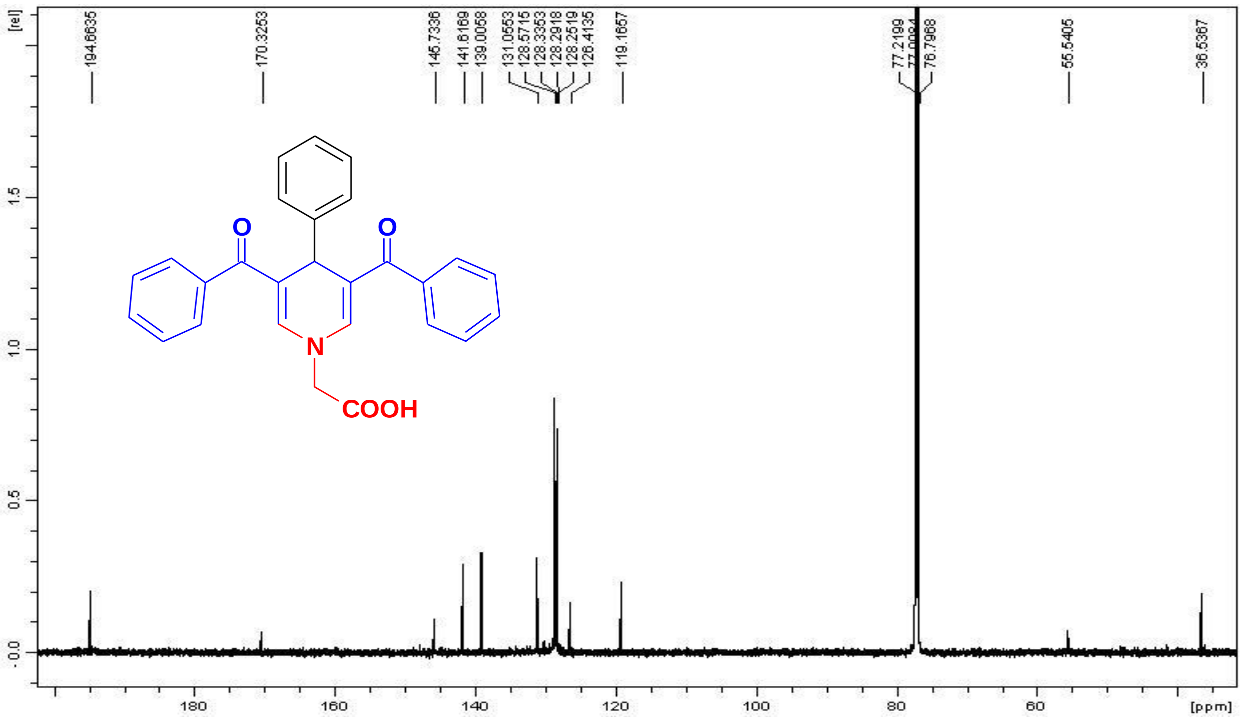

Compound **2o**

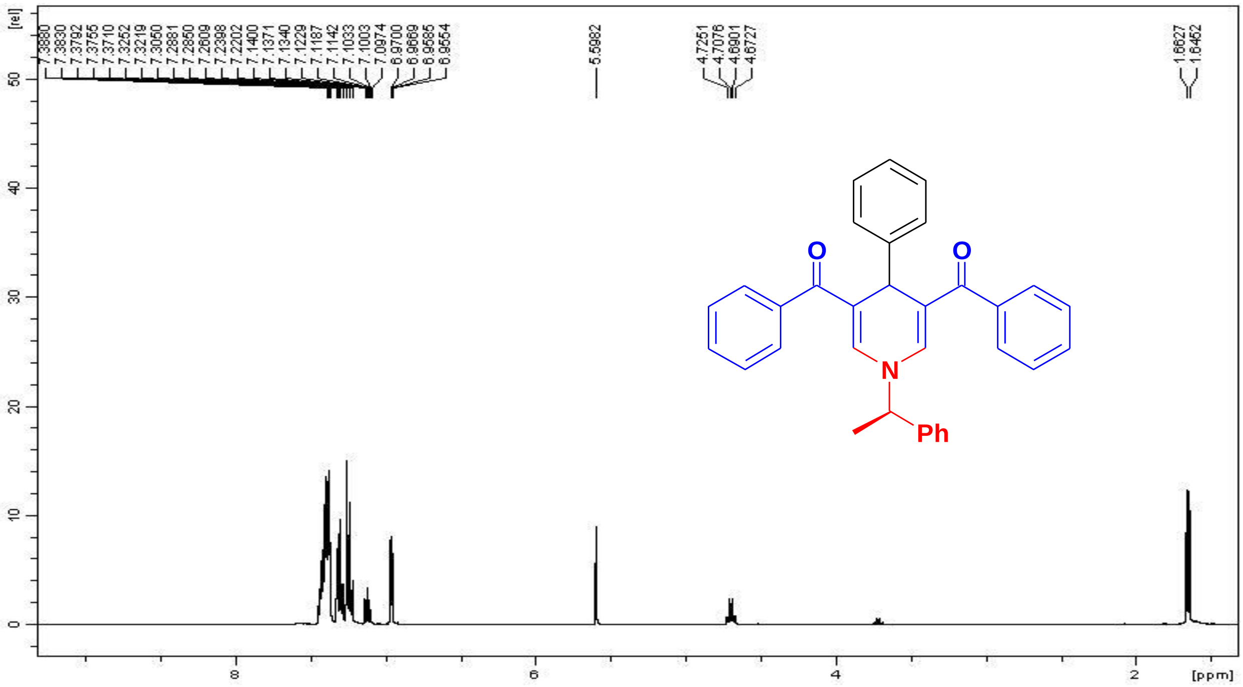

Compound **4a**

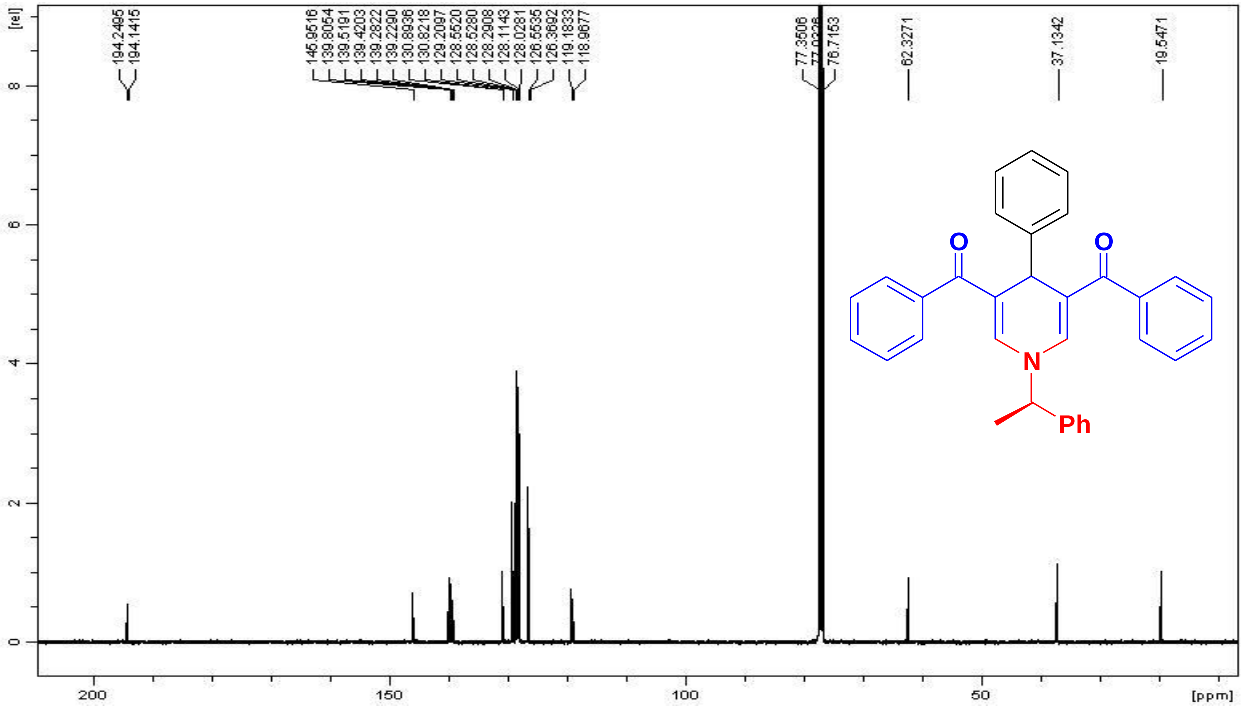

Compound **4a**

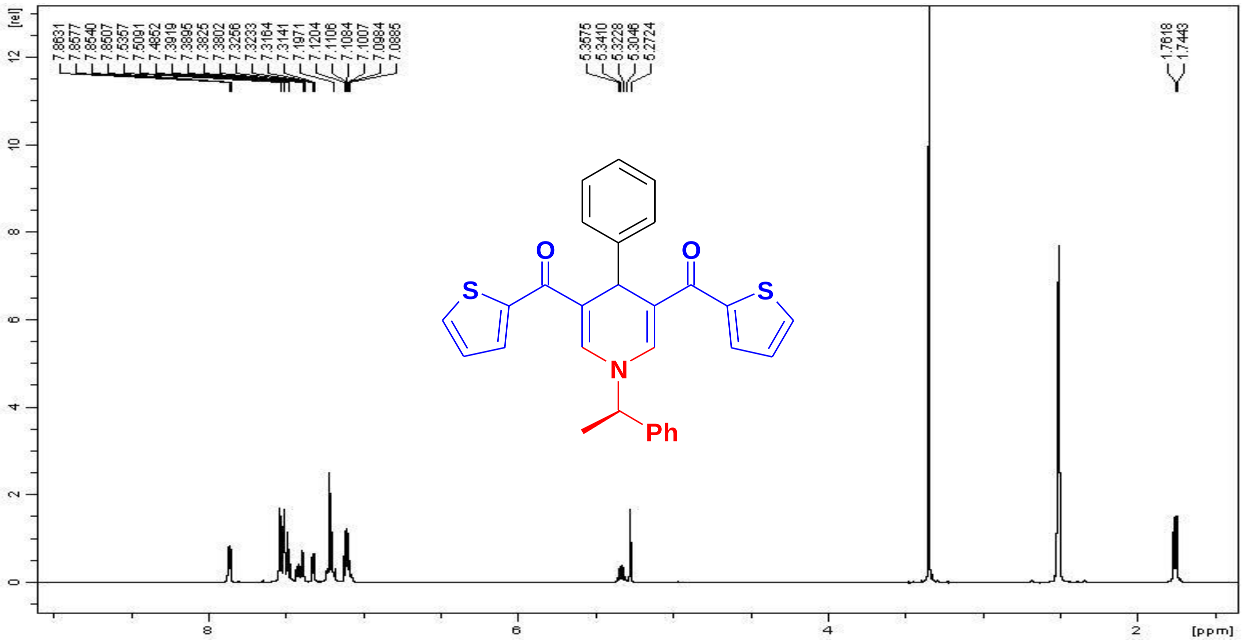

Compound **4b**

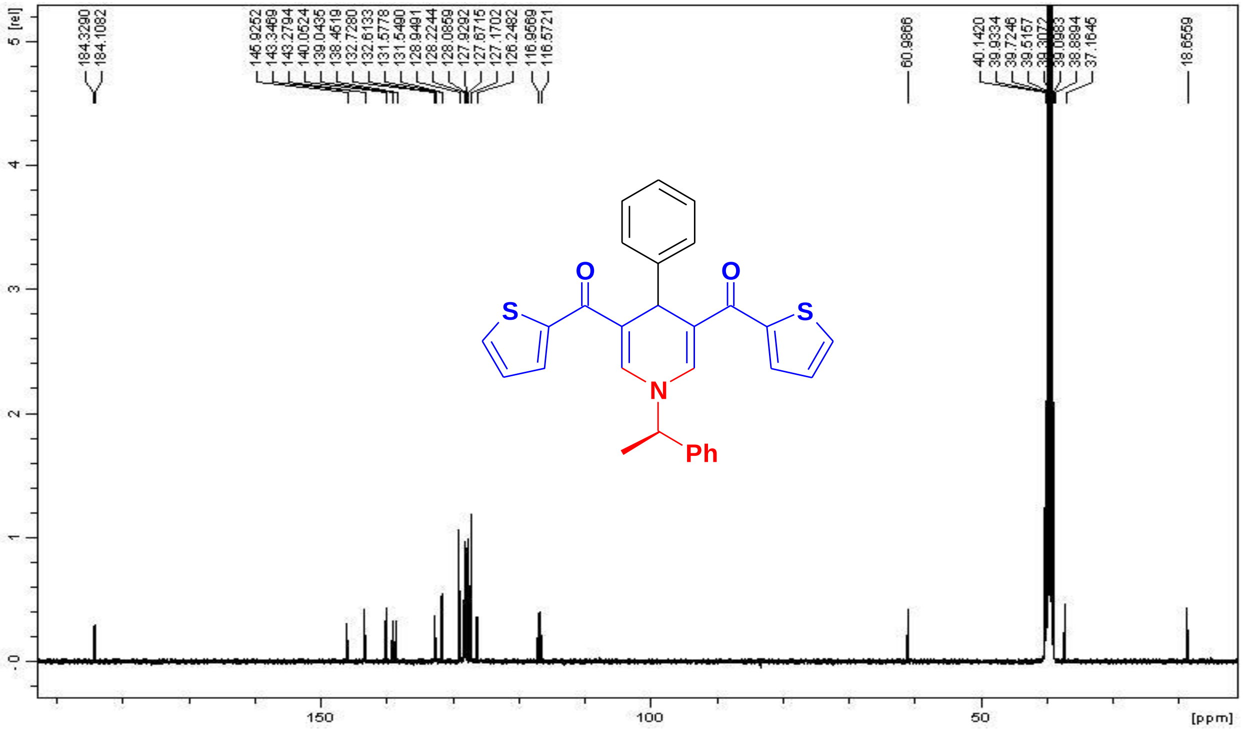

Compound **4b**

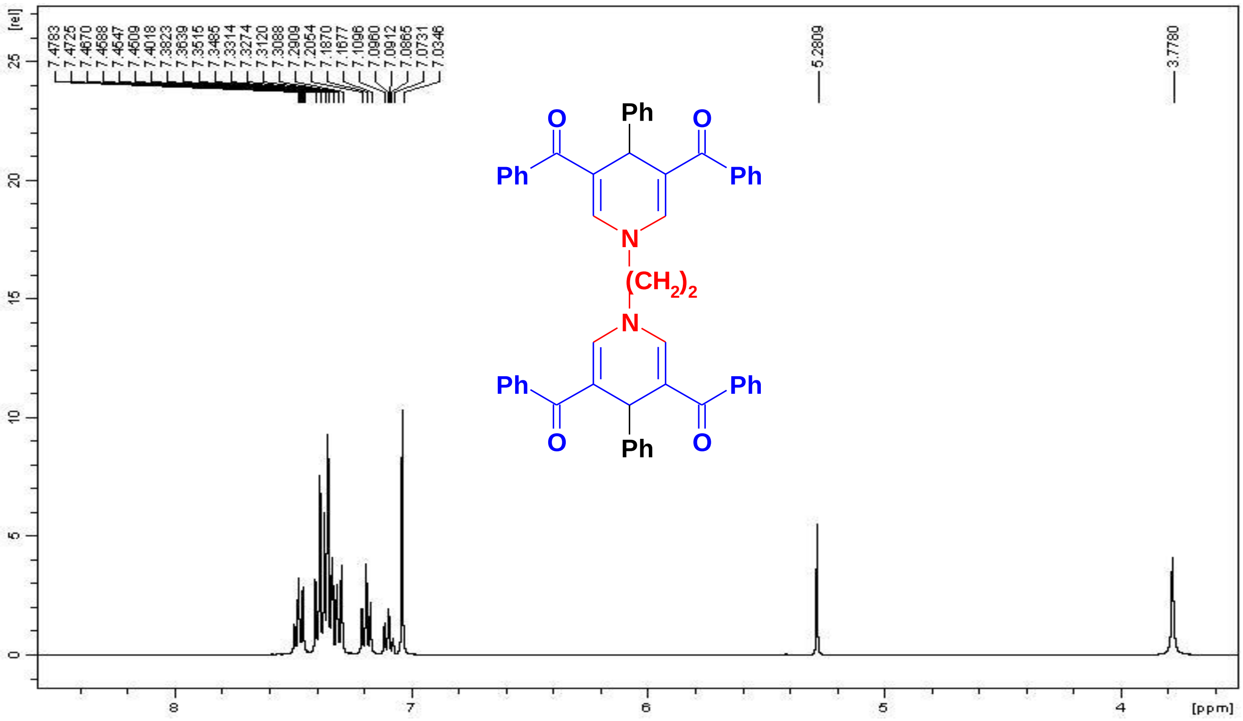

Compound **5a**

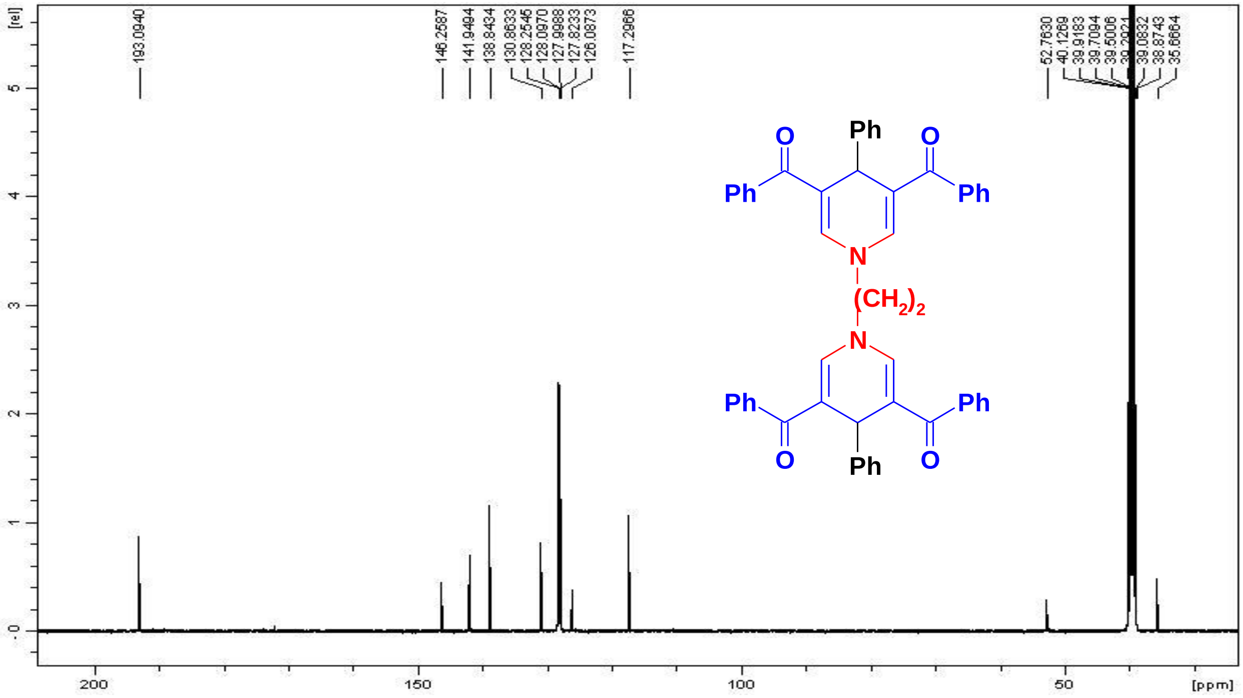

Compound **5a**

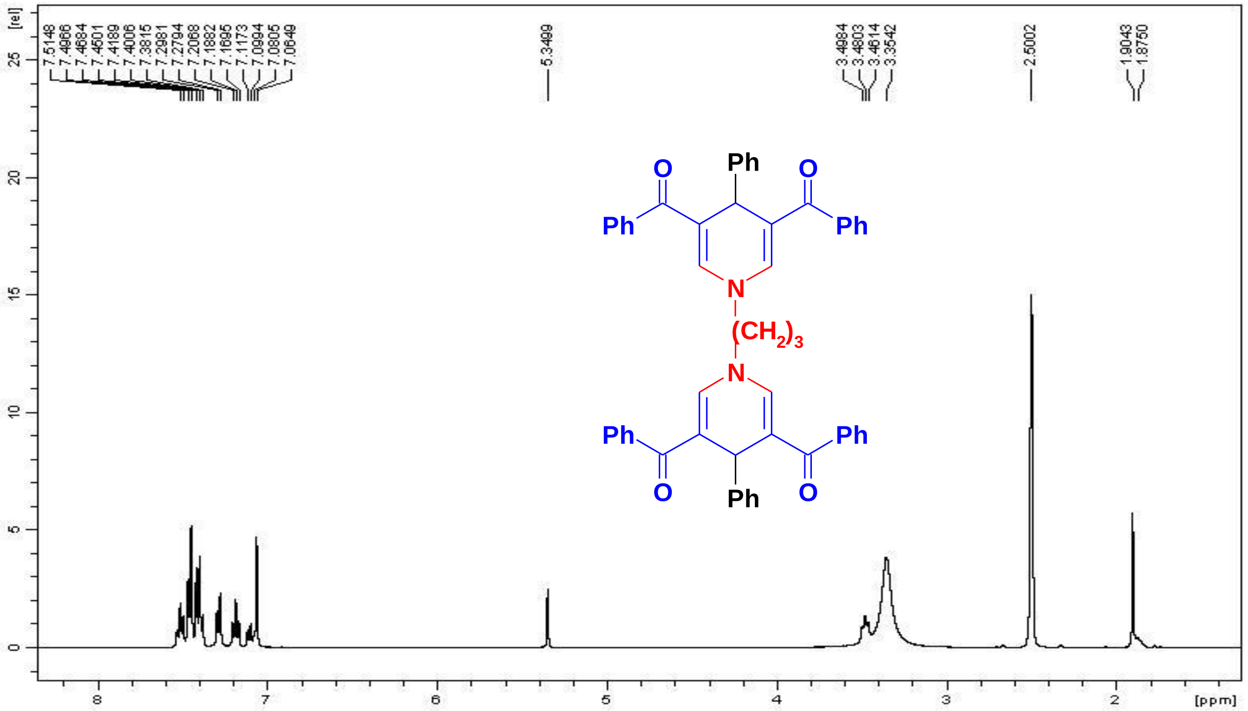

Compound **5b**

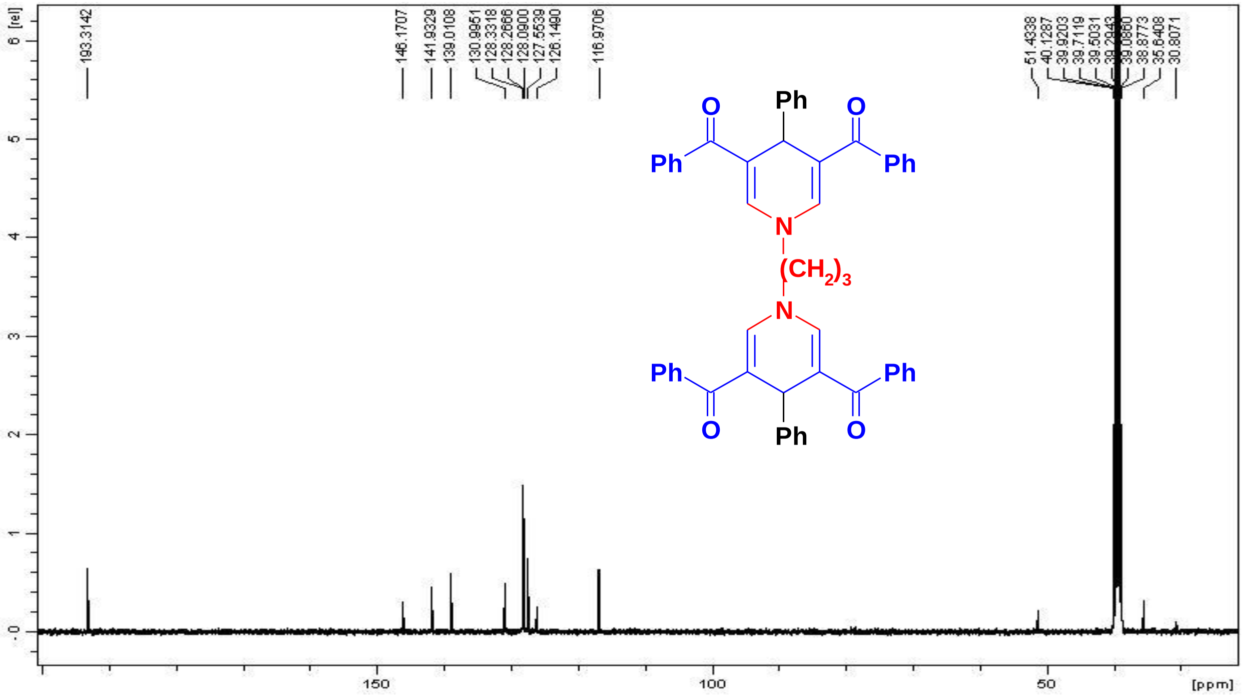

Compound **5b**

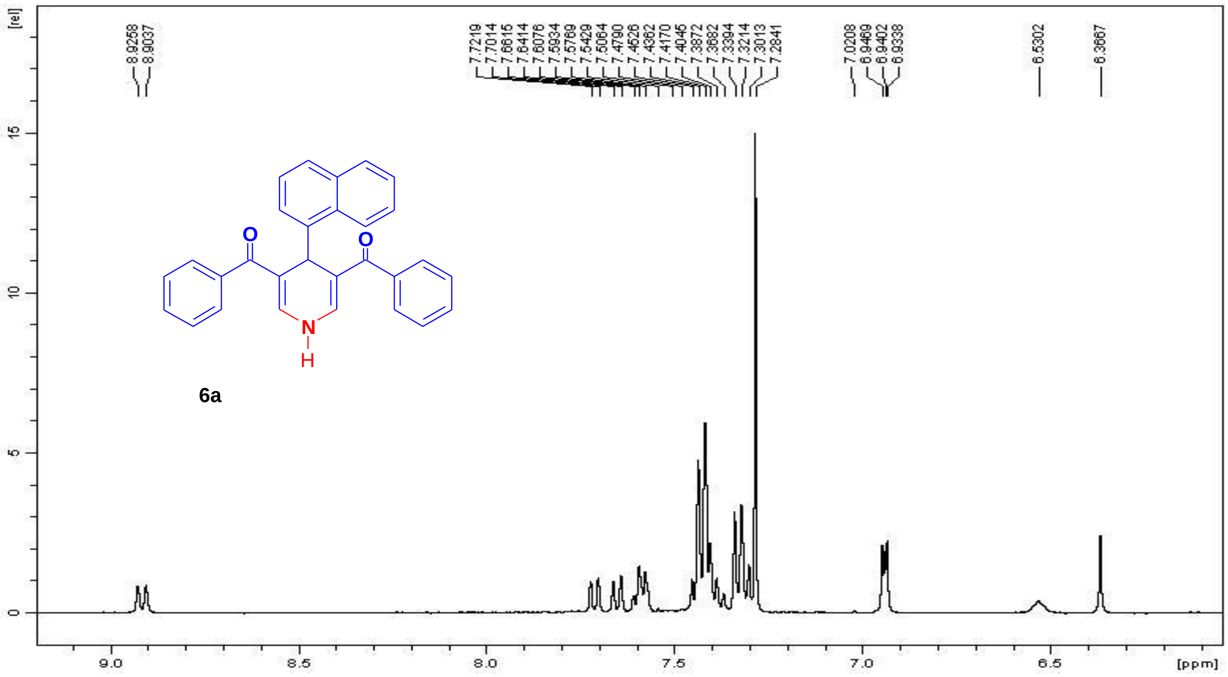

Compound **6a**.

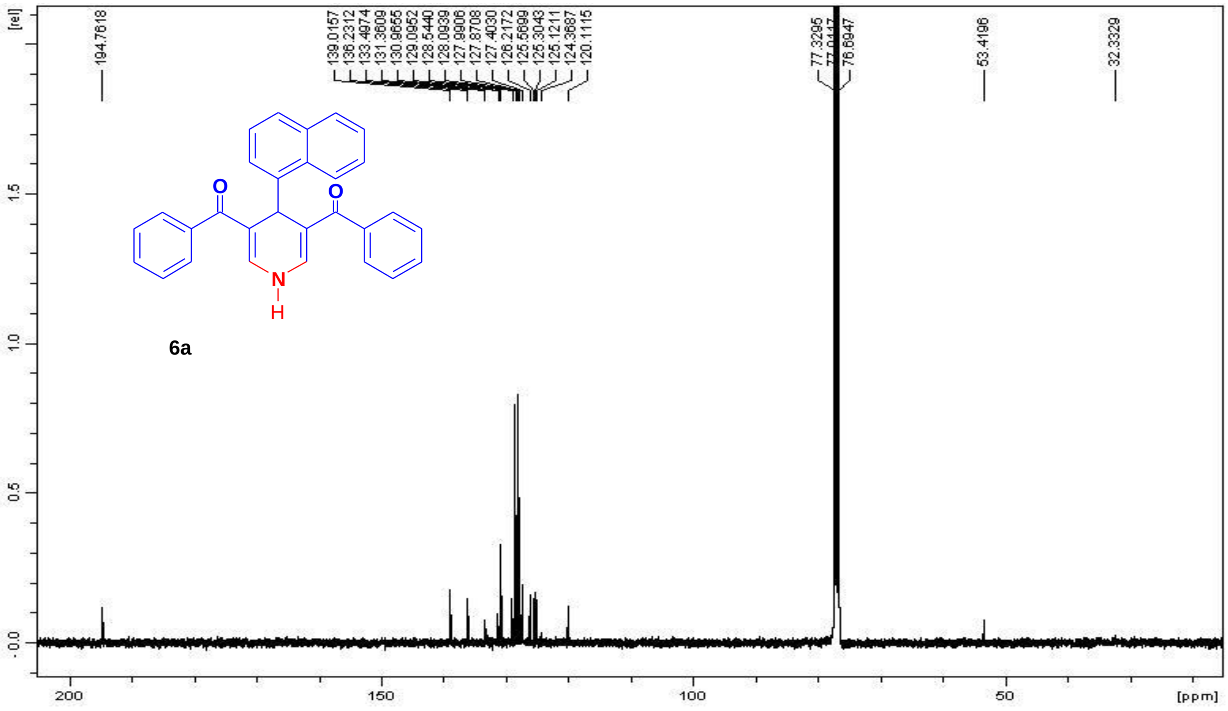

Compound **6a**.

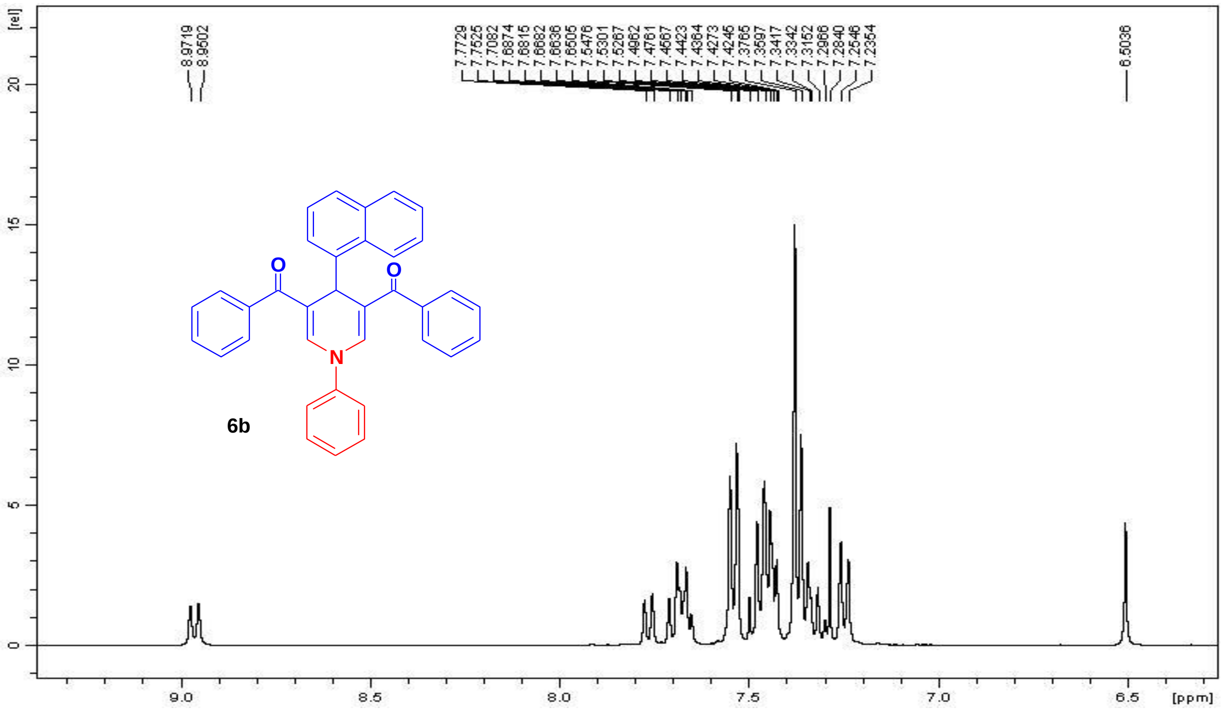

Compound **6b**.

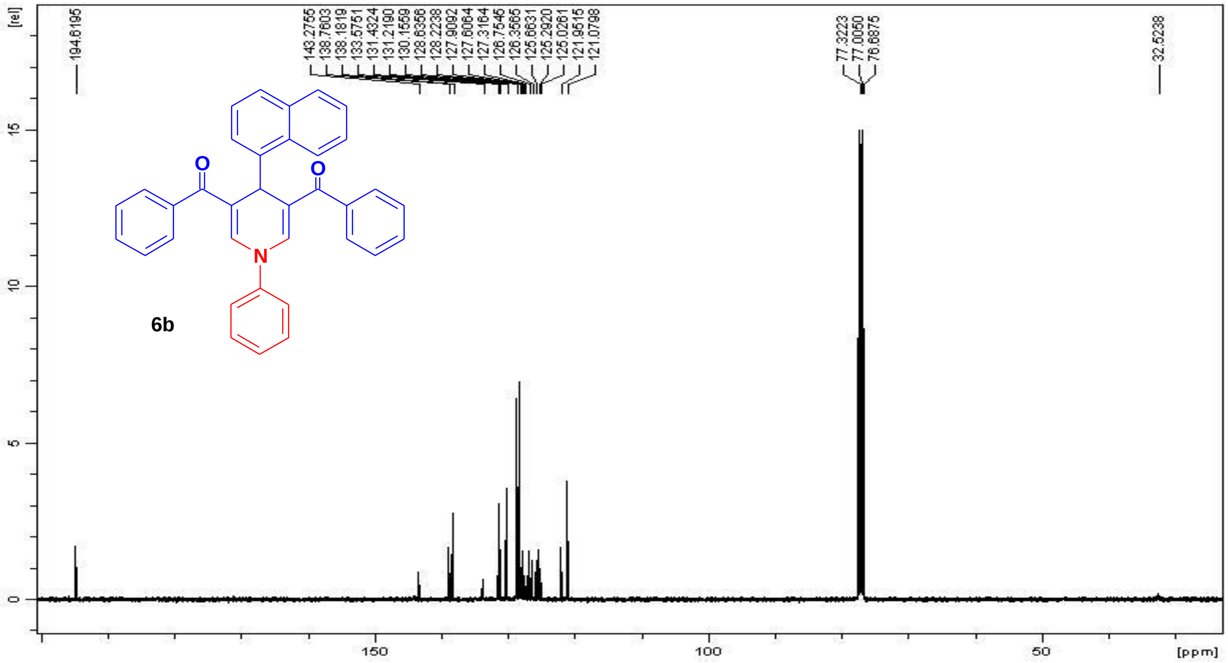

Compound **6b**.

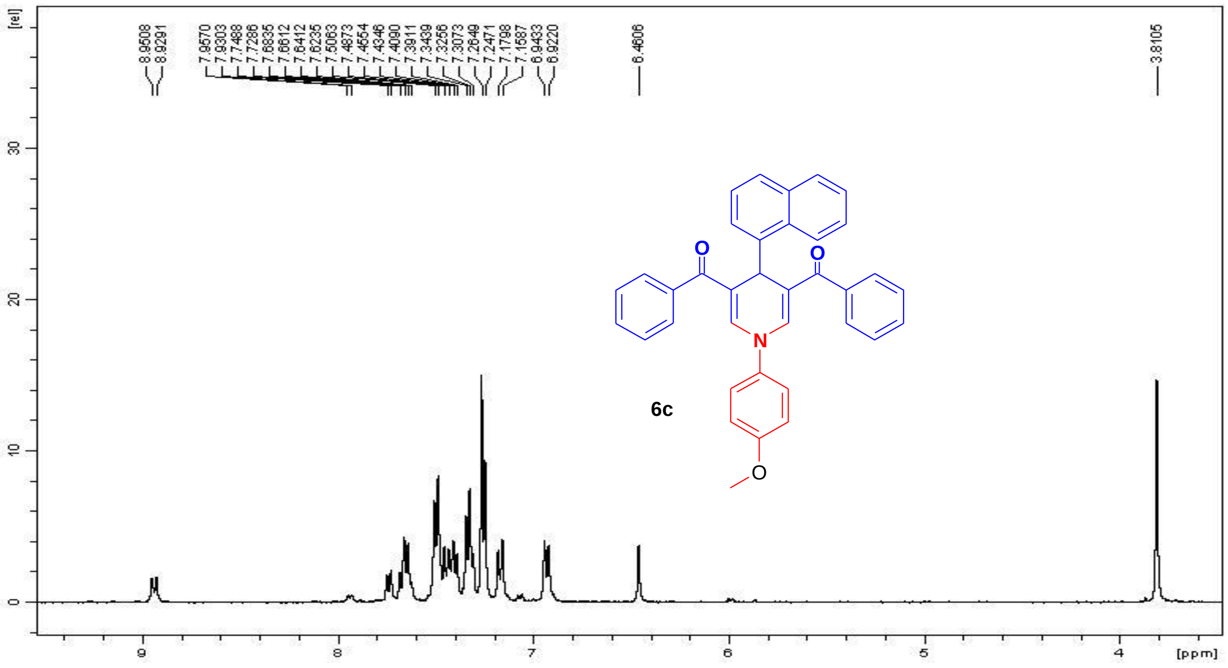

Compound **6c**.

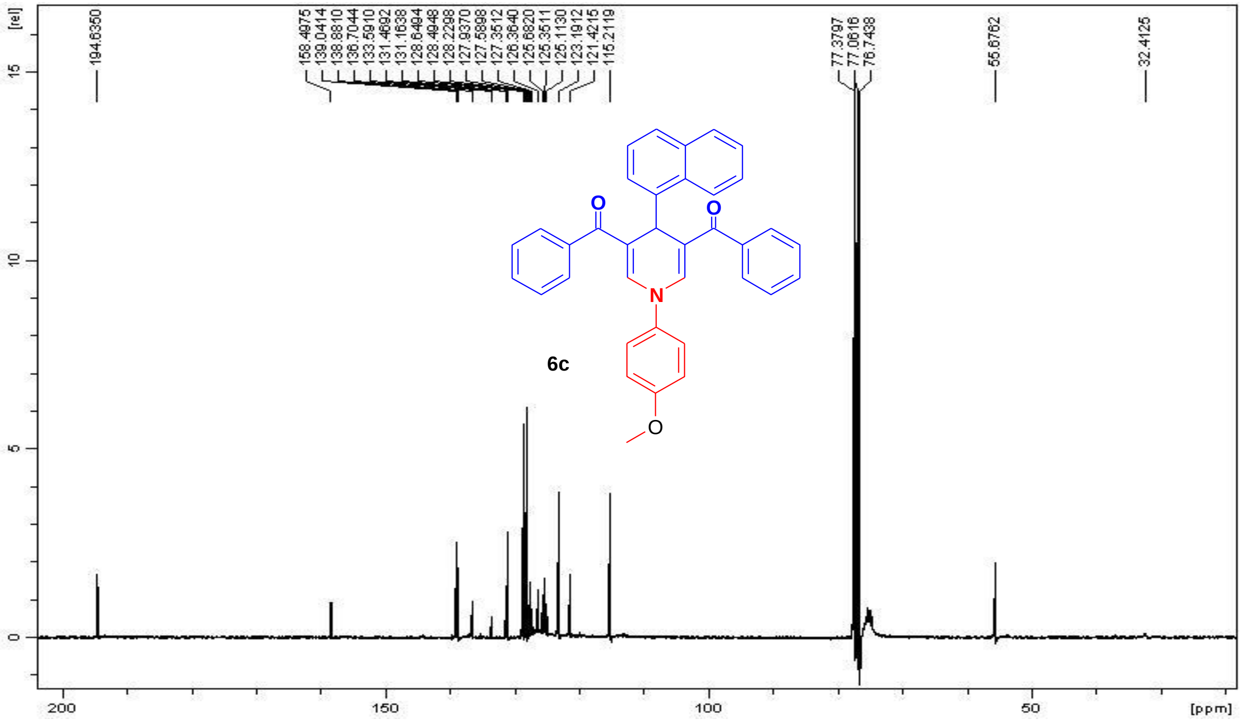

Compound **6c**.

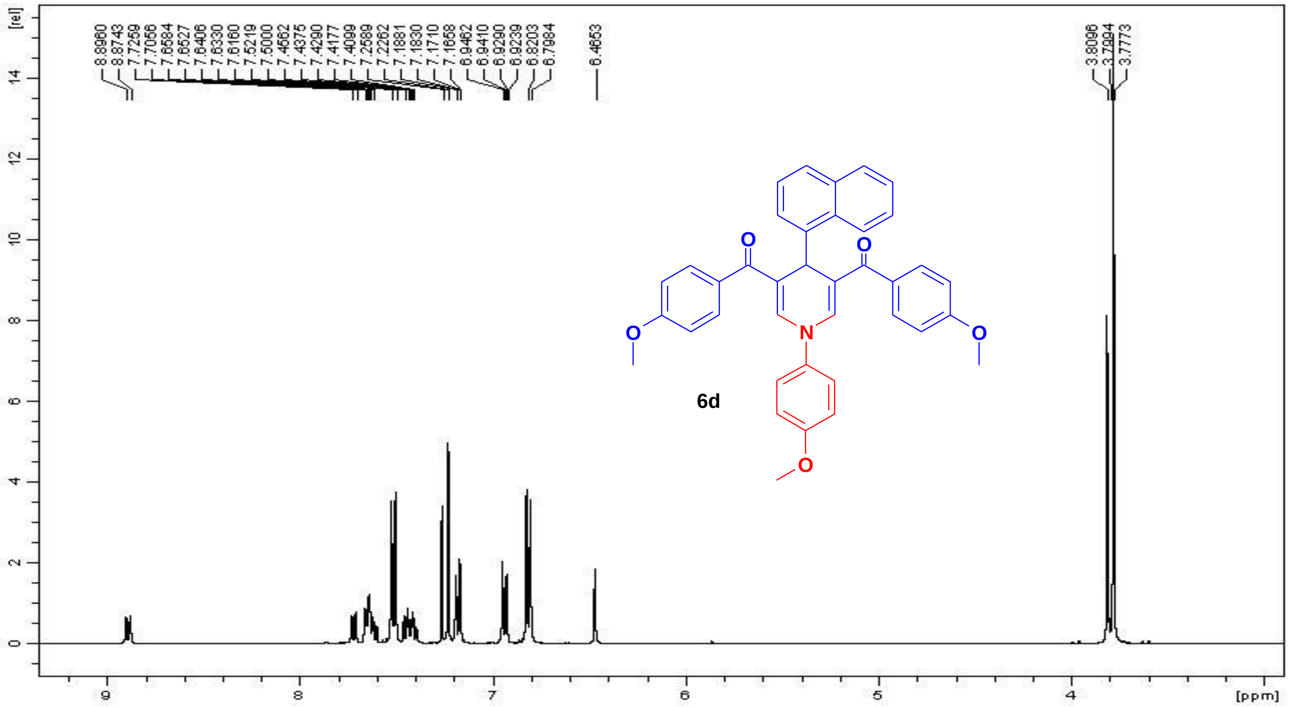

Compound **6d**.

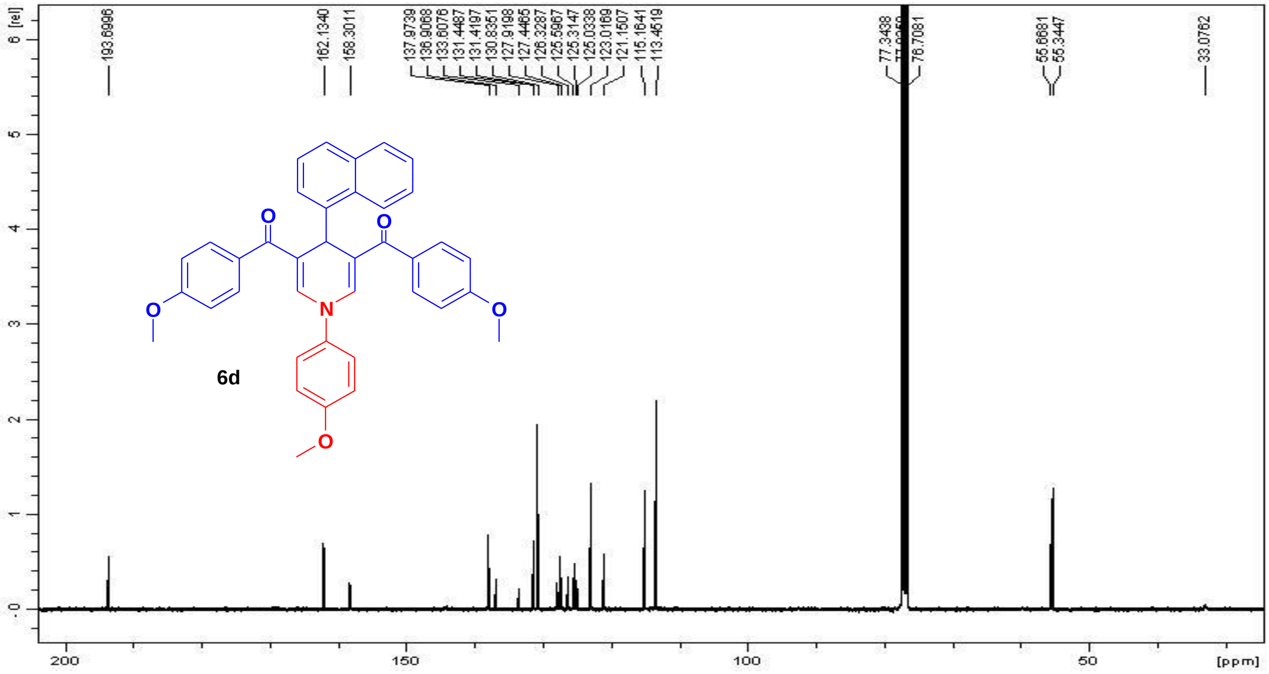

Compound **6d**.

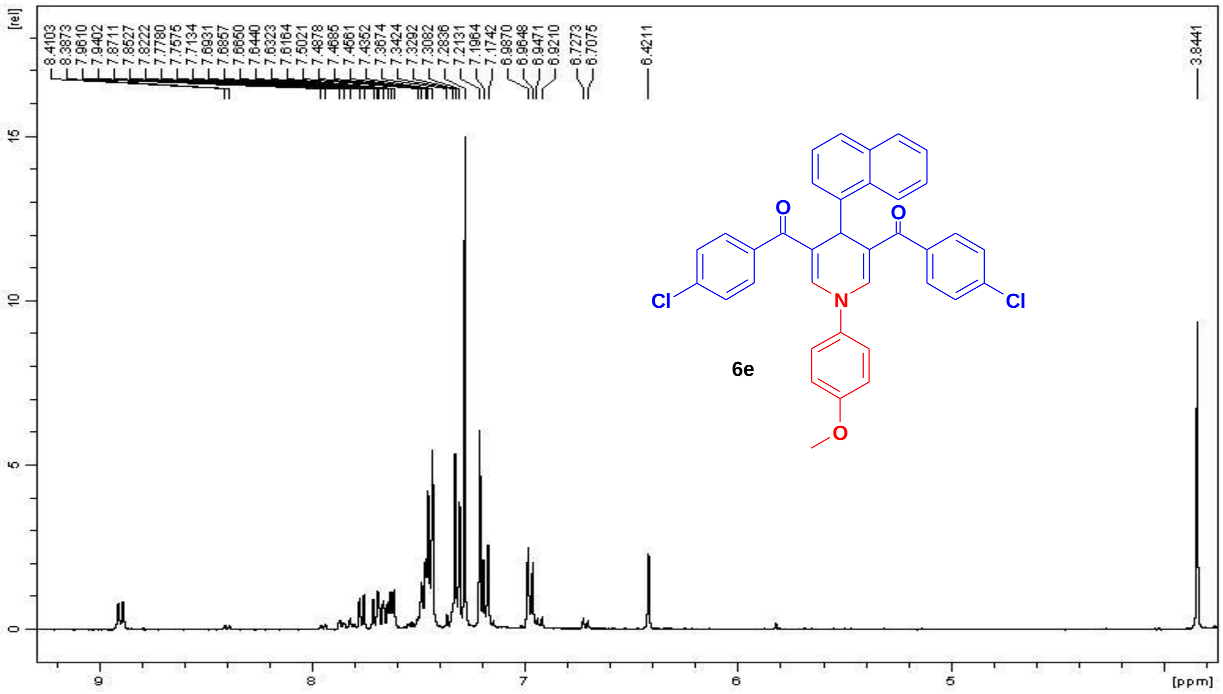

Compound **6e**.

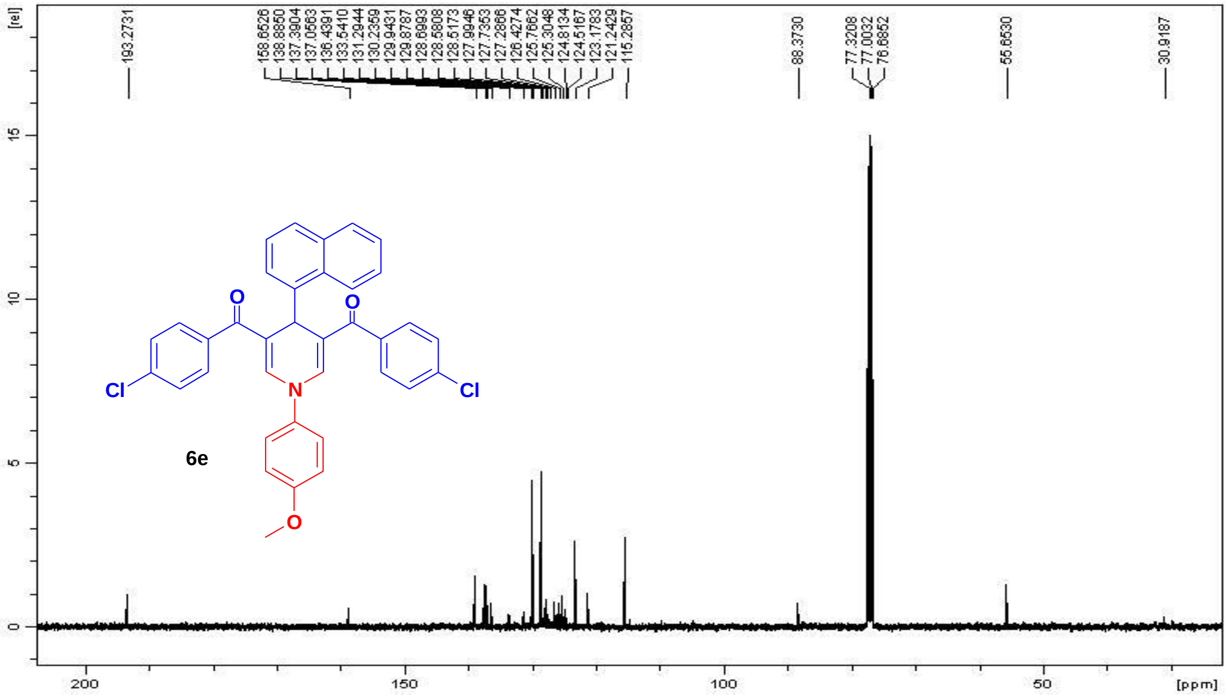

Compound **6e**.

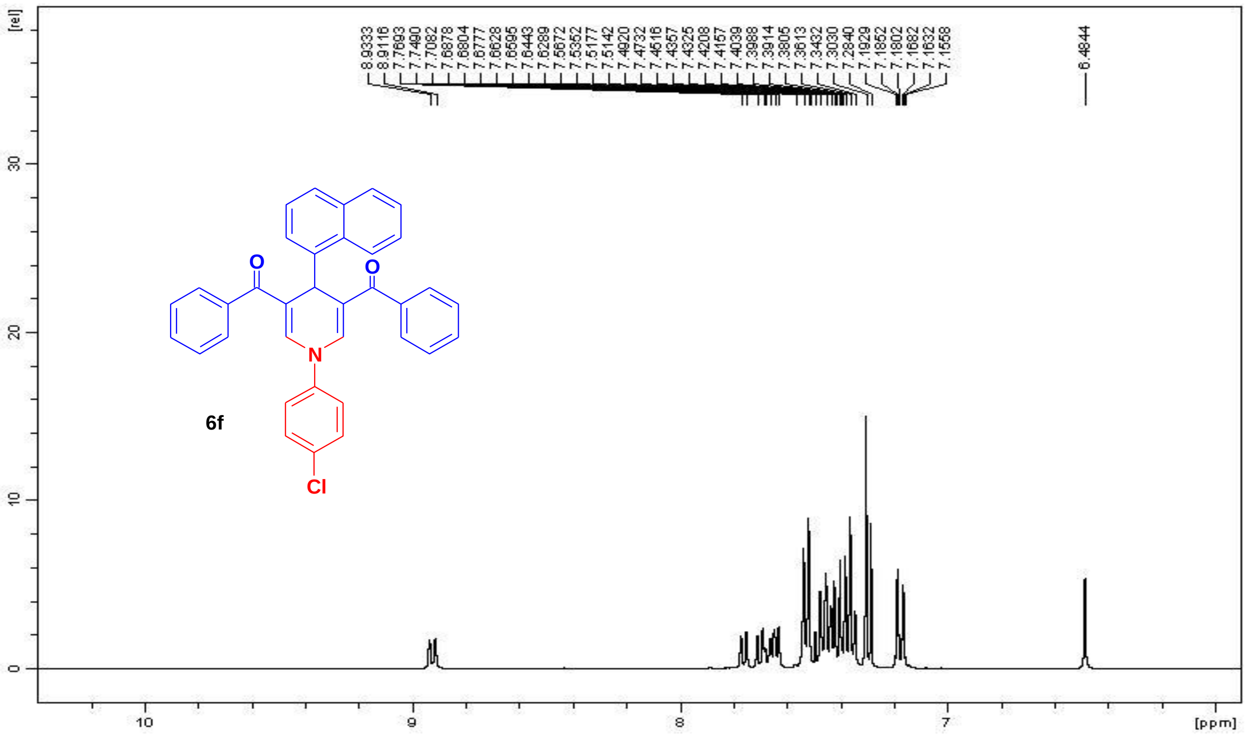

Compound **6f**

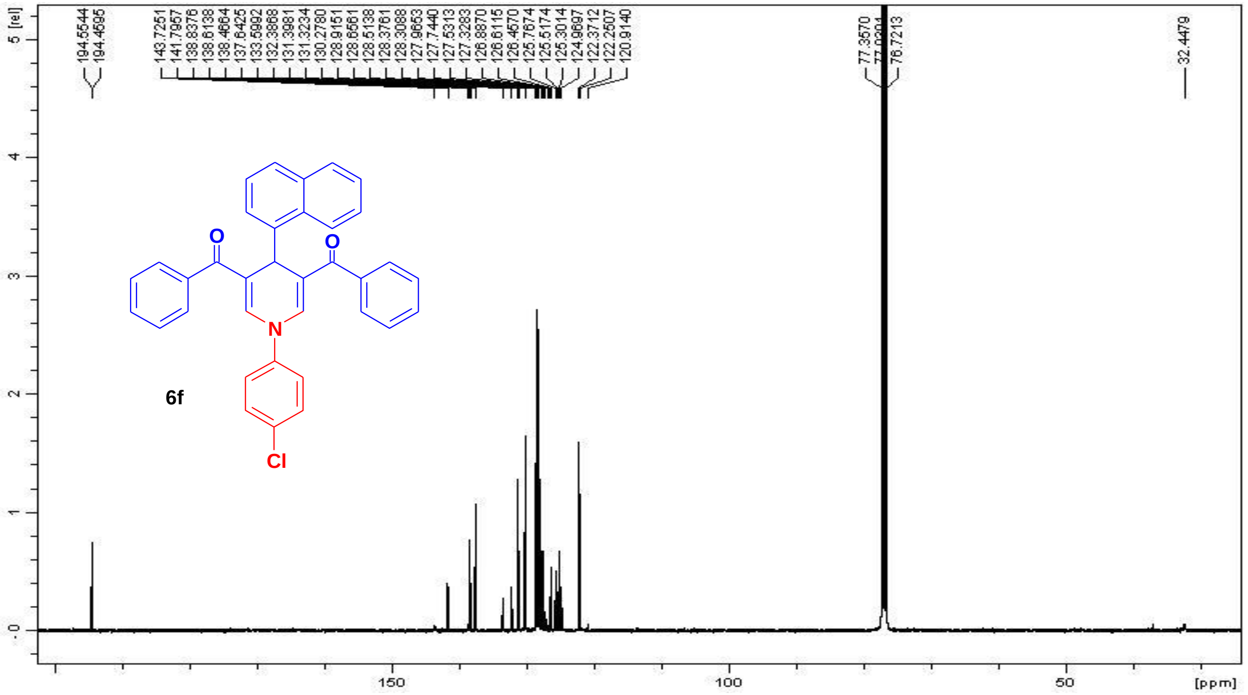

Compound **6f**

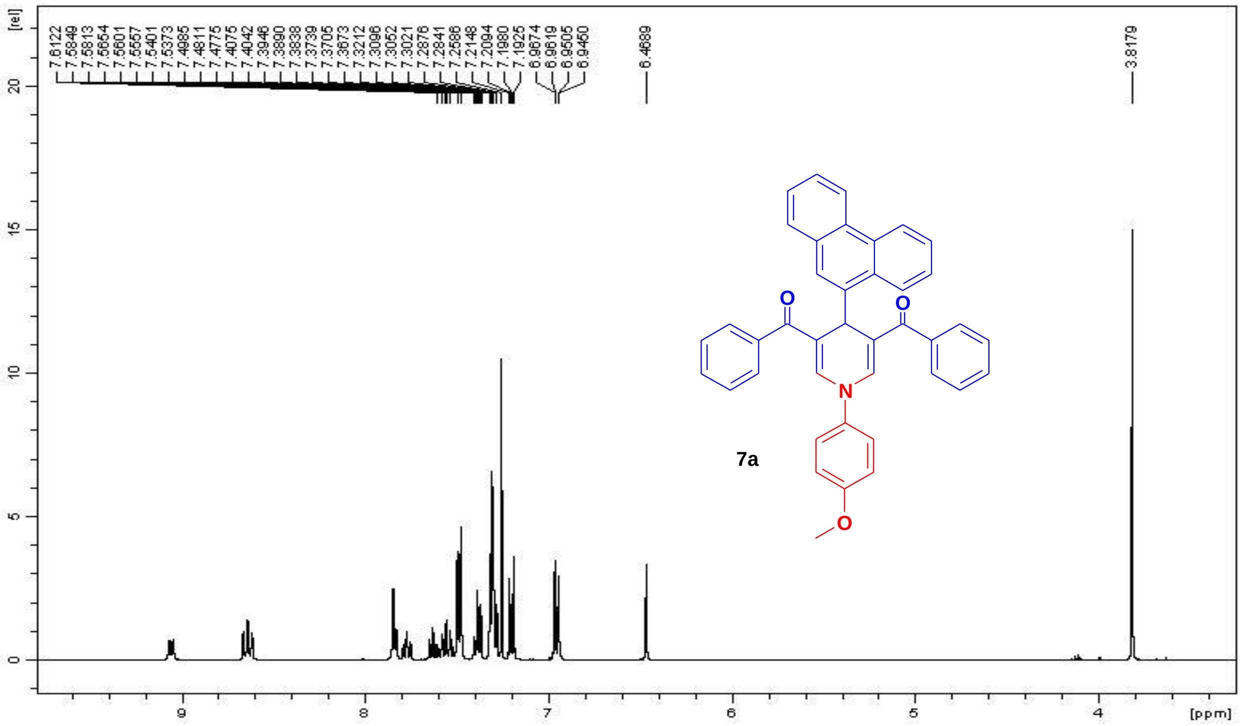

Compound **7a**.

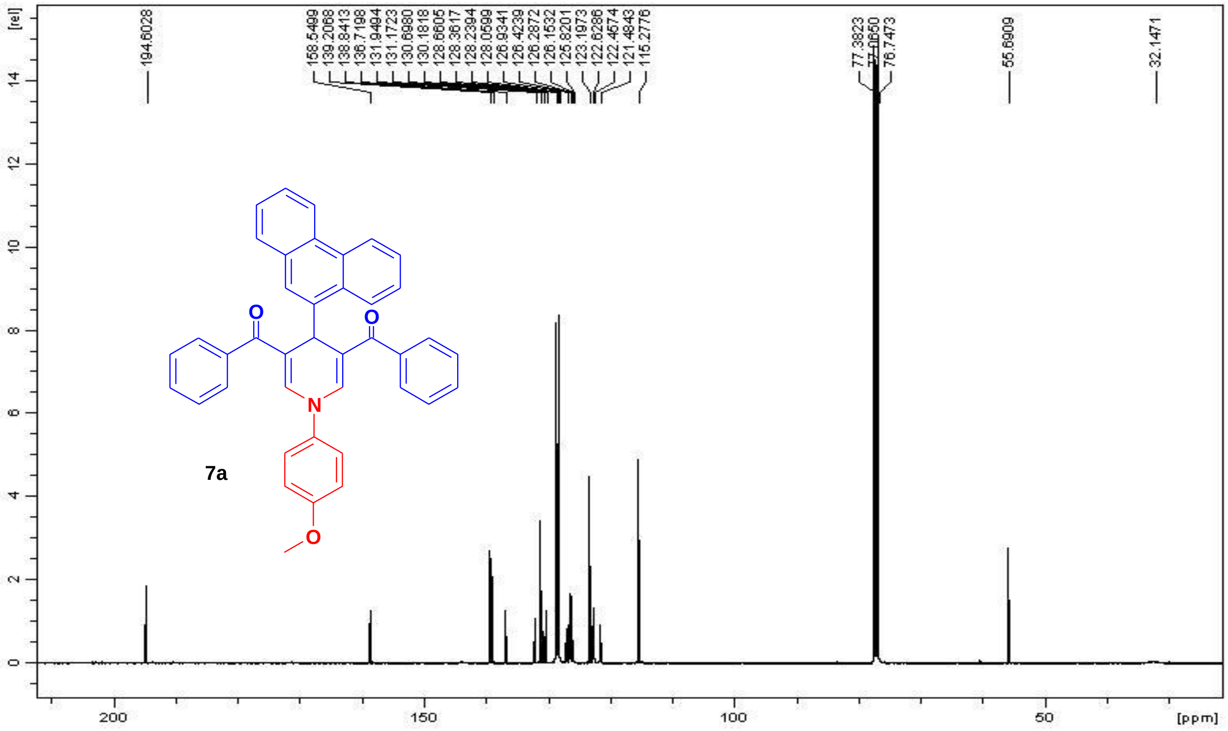

Compound **7a**.

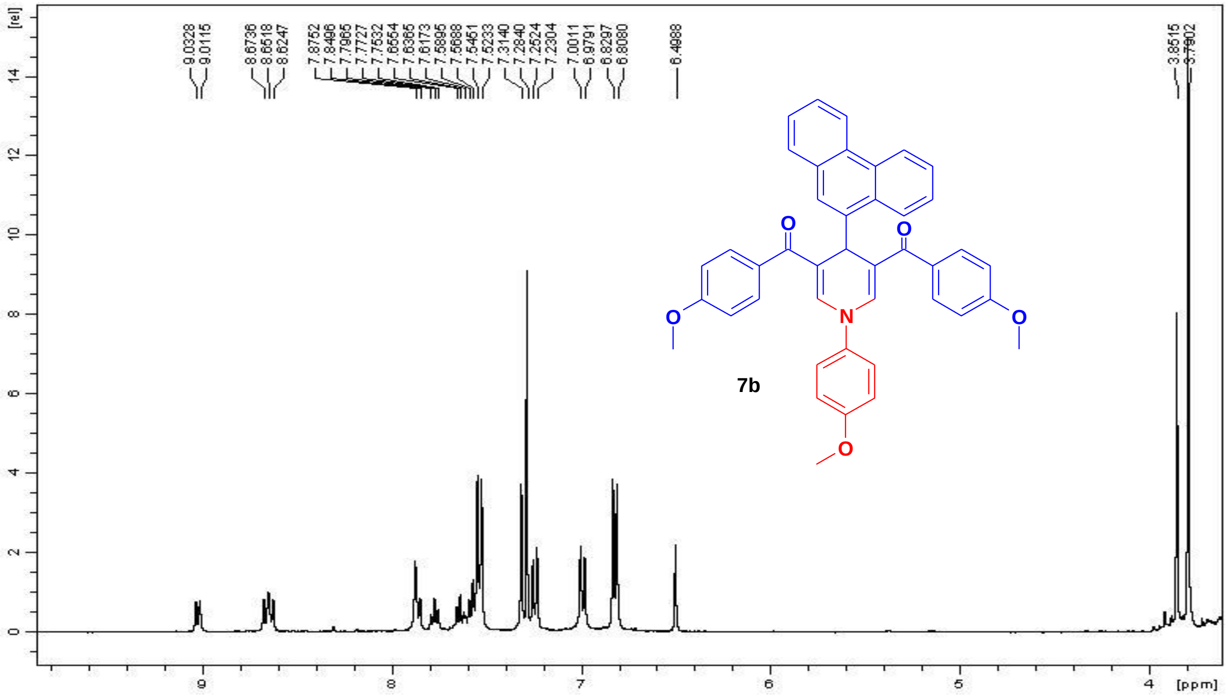

Compound **7b**

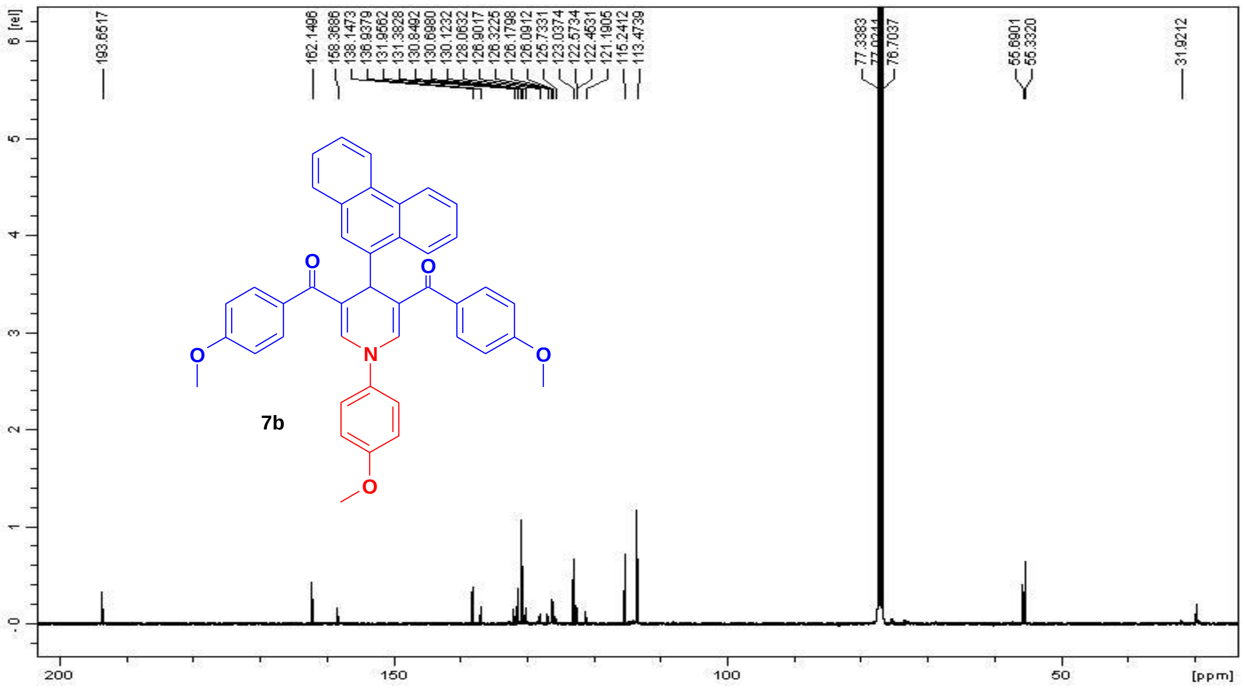

Compound **7b**

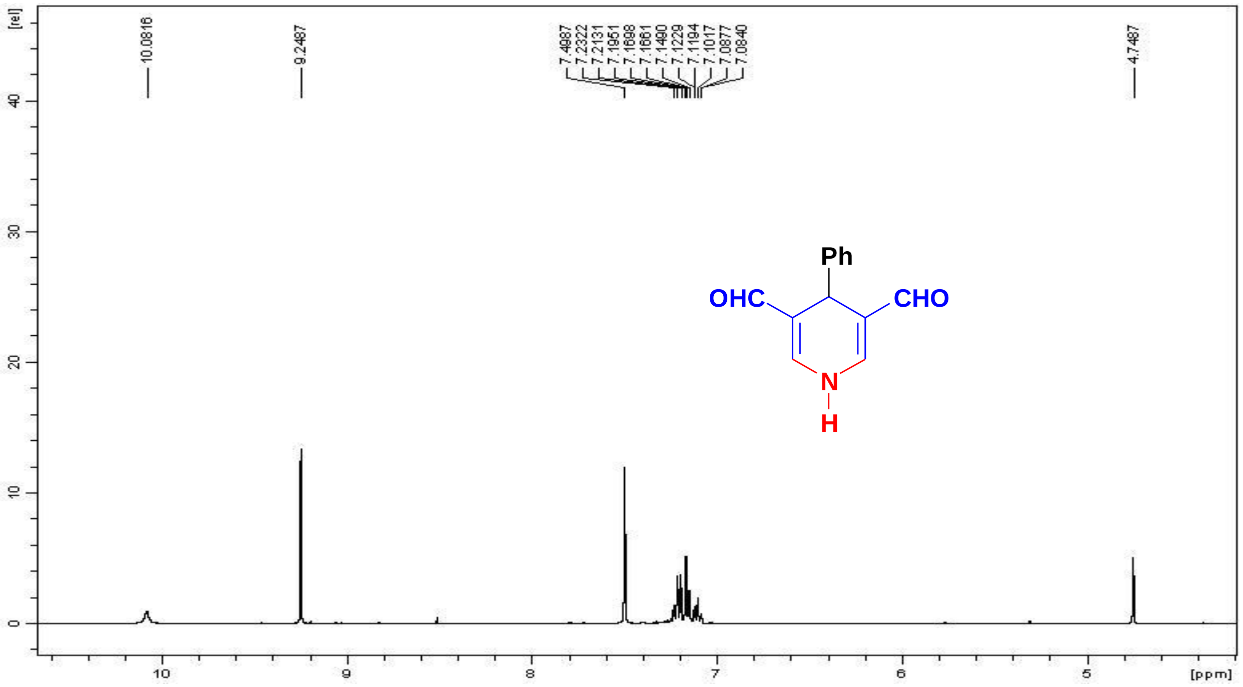

Compound **10a**

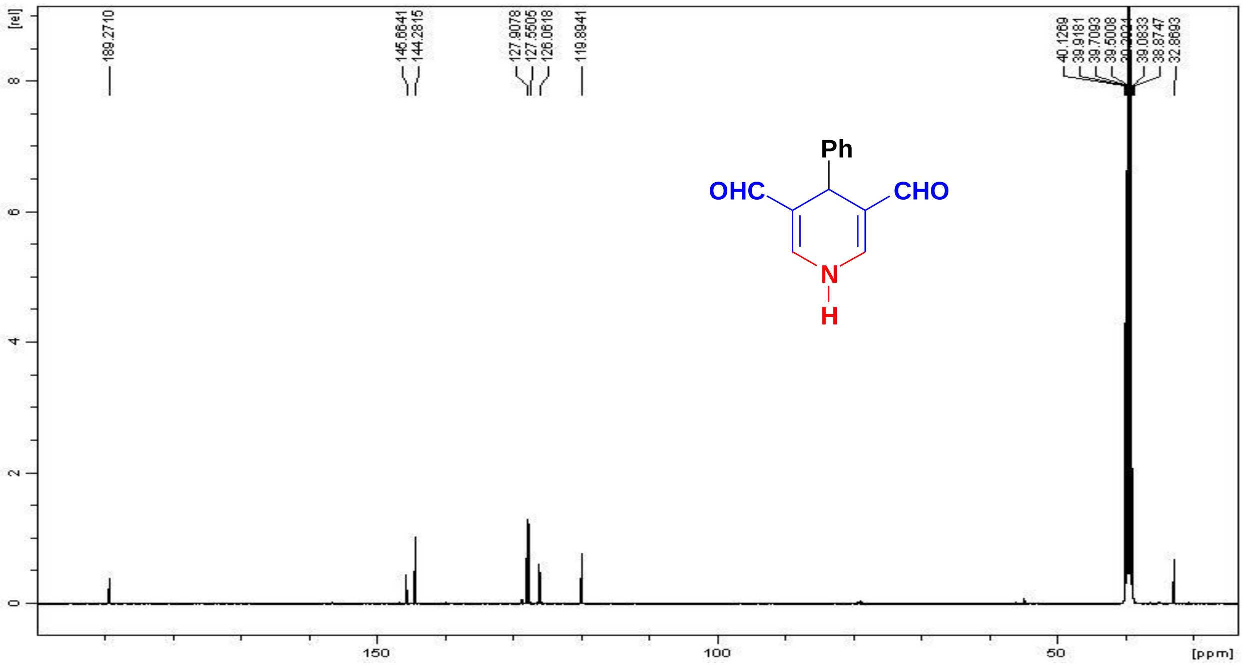

Compound **10a**

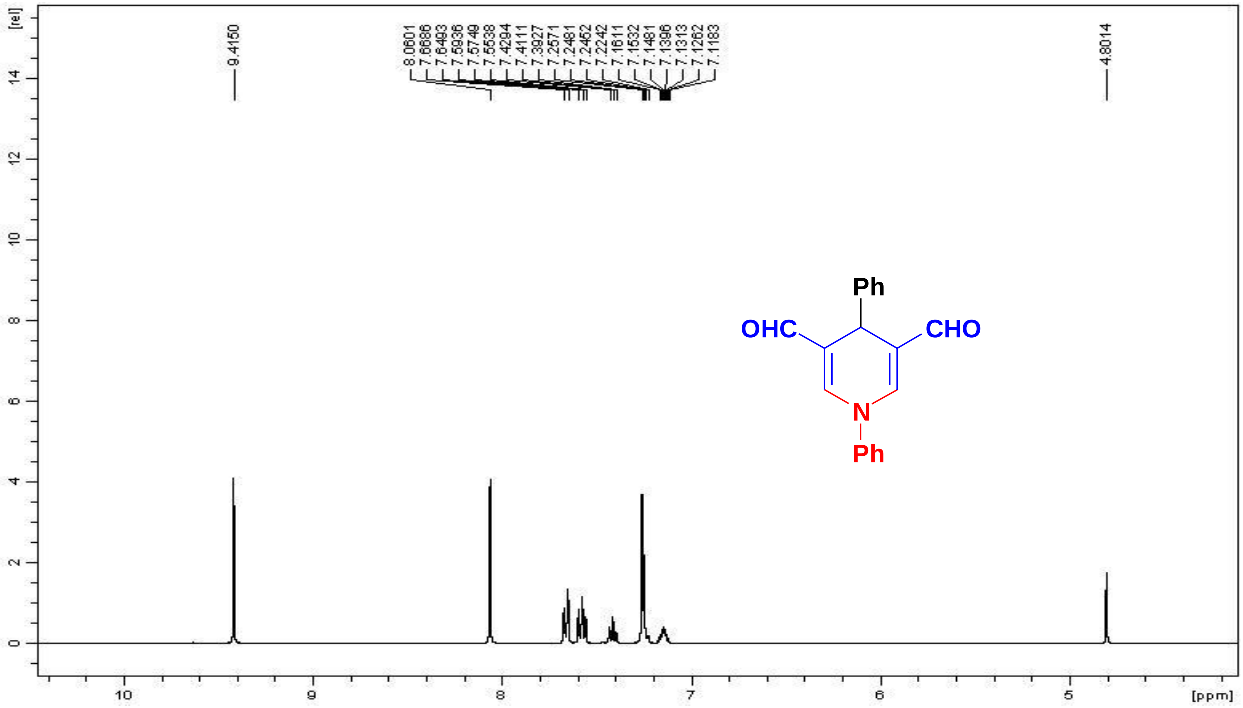

Compound **10b**

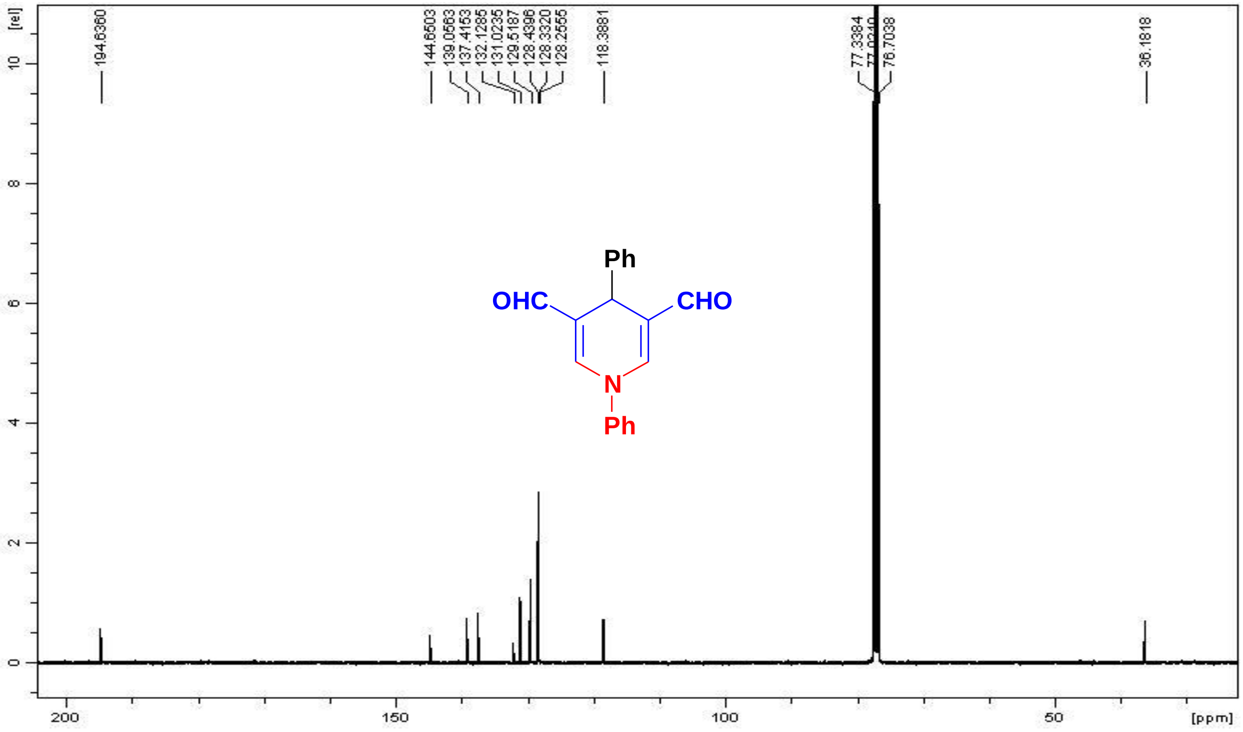

Compound **10b**

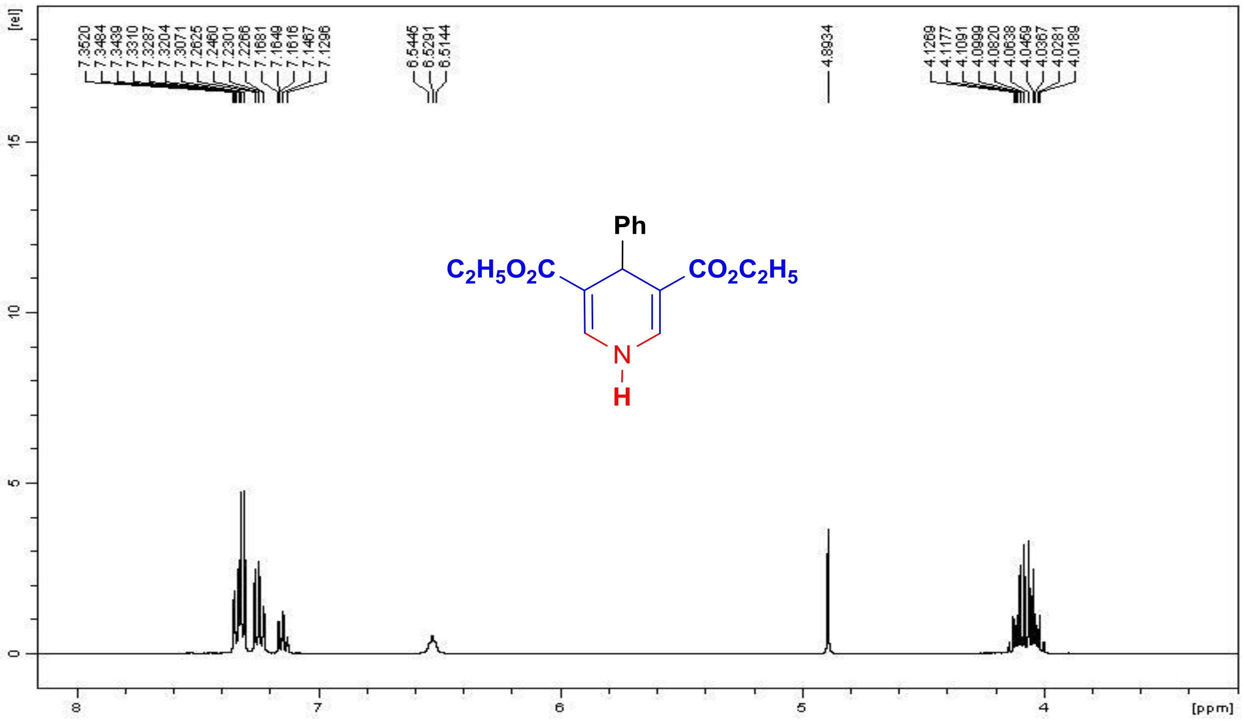

Compound **12**

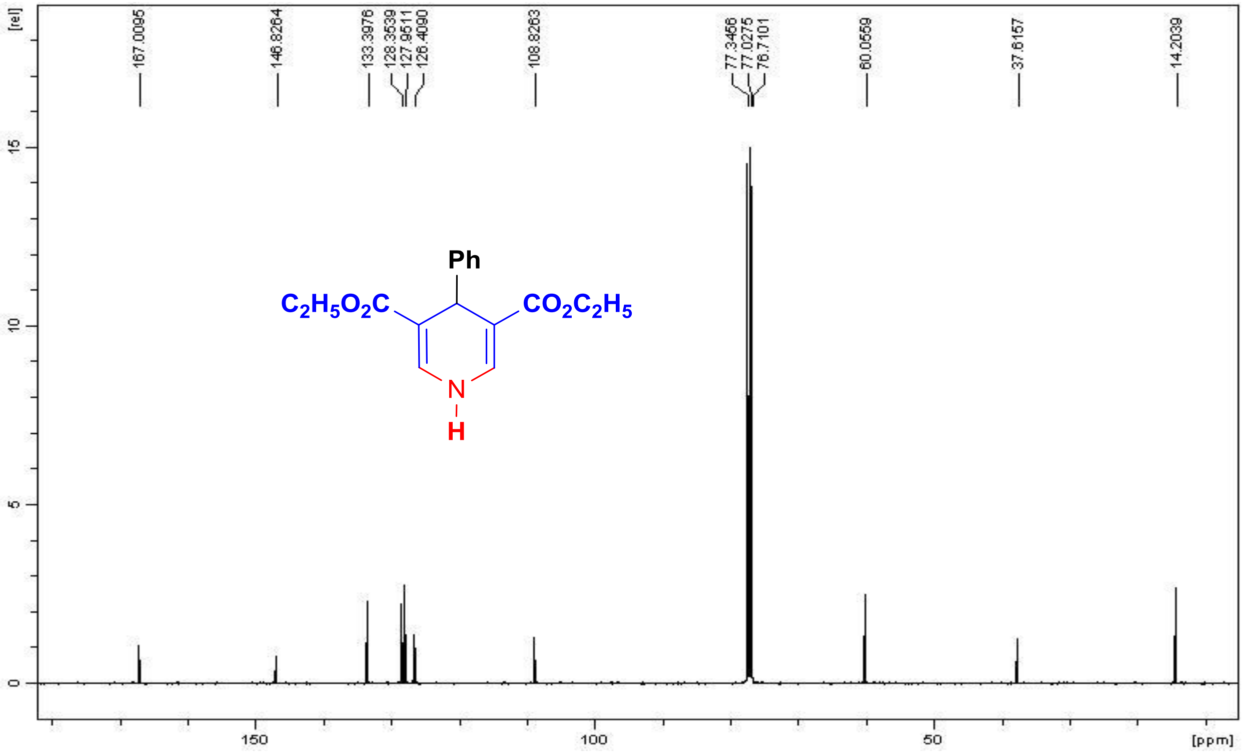

Compound **12**

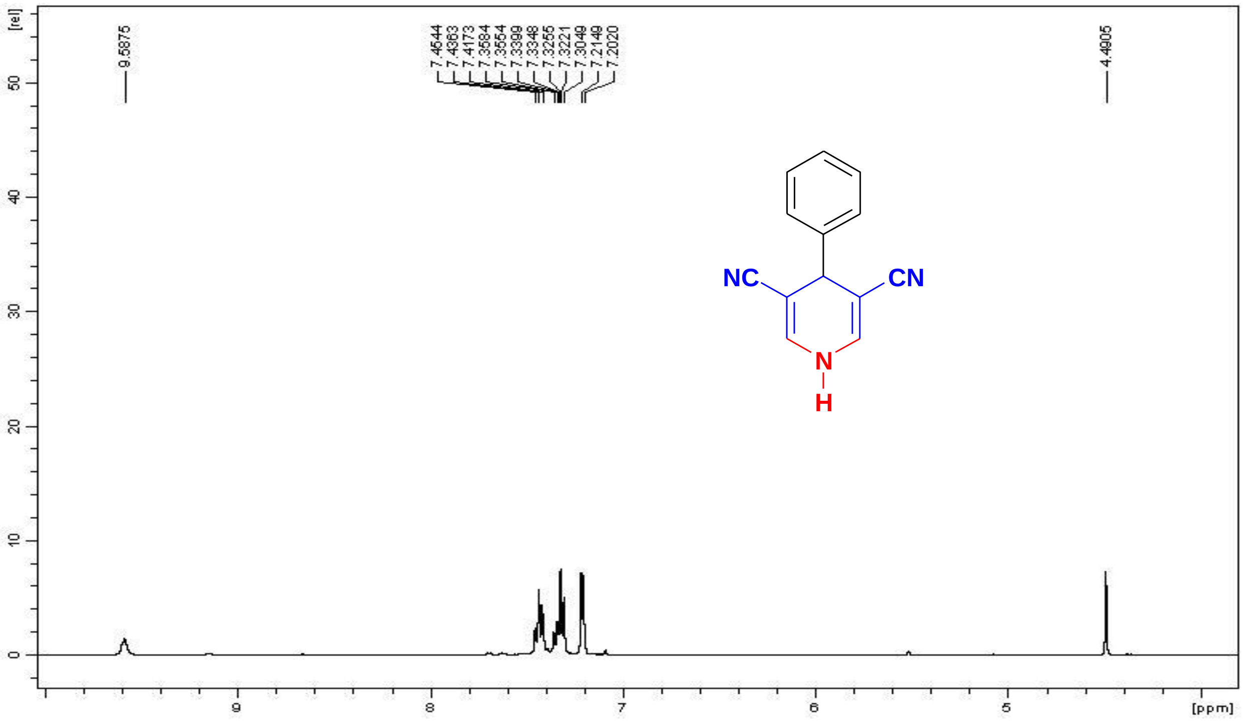

Compound **14**

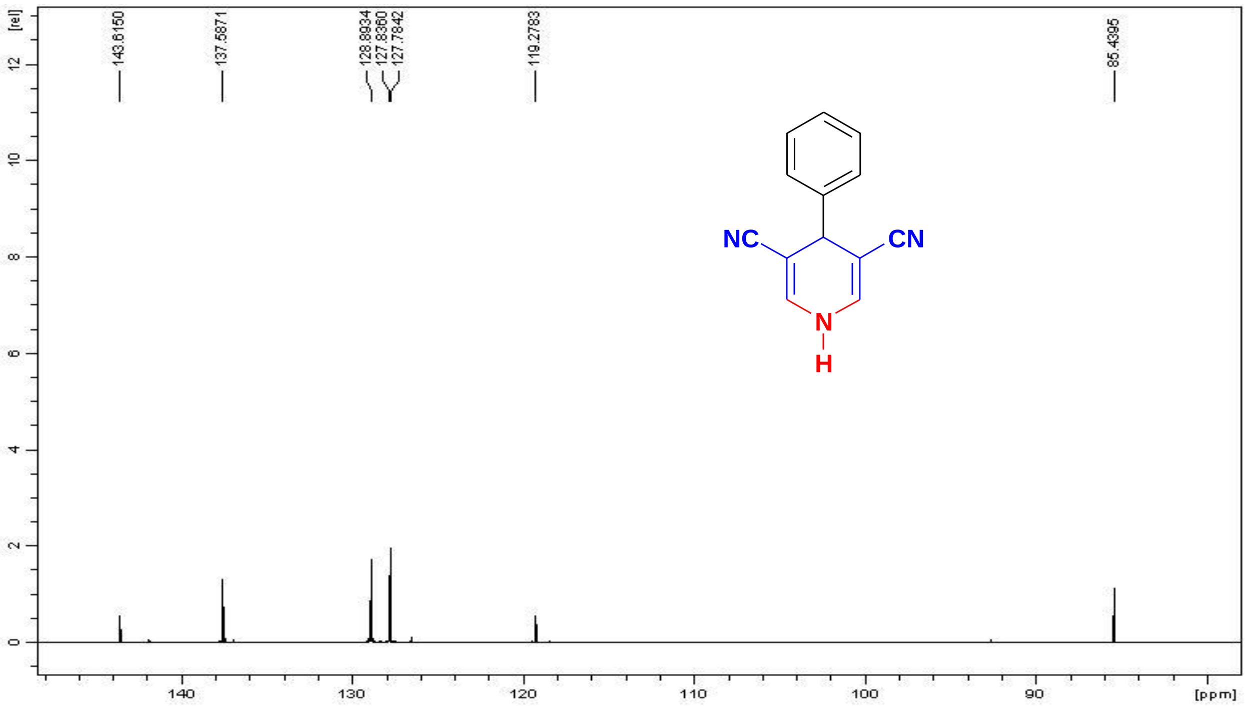

Compound **14**

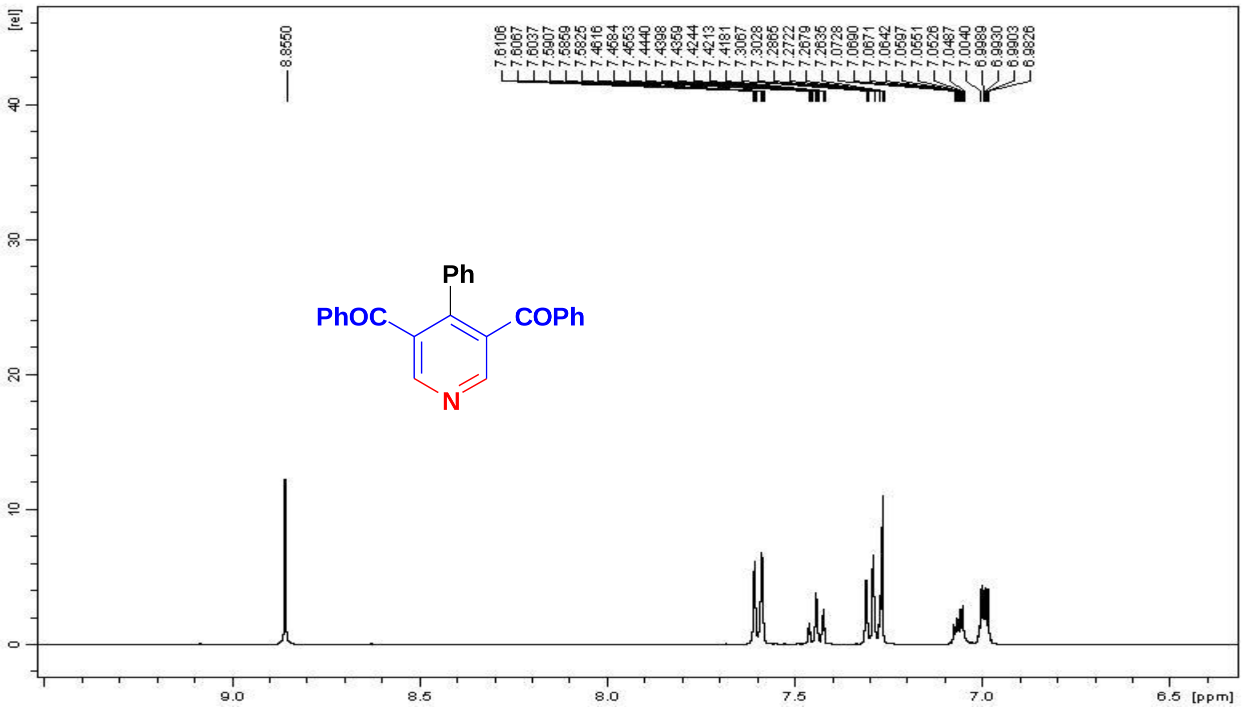

Compound **15a**

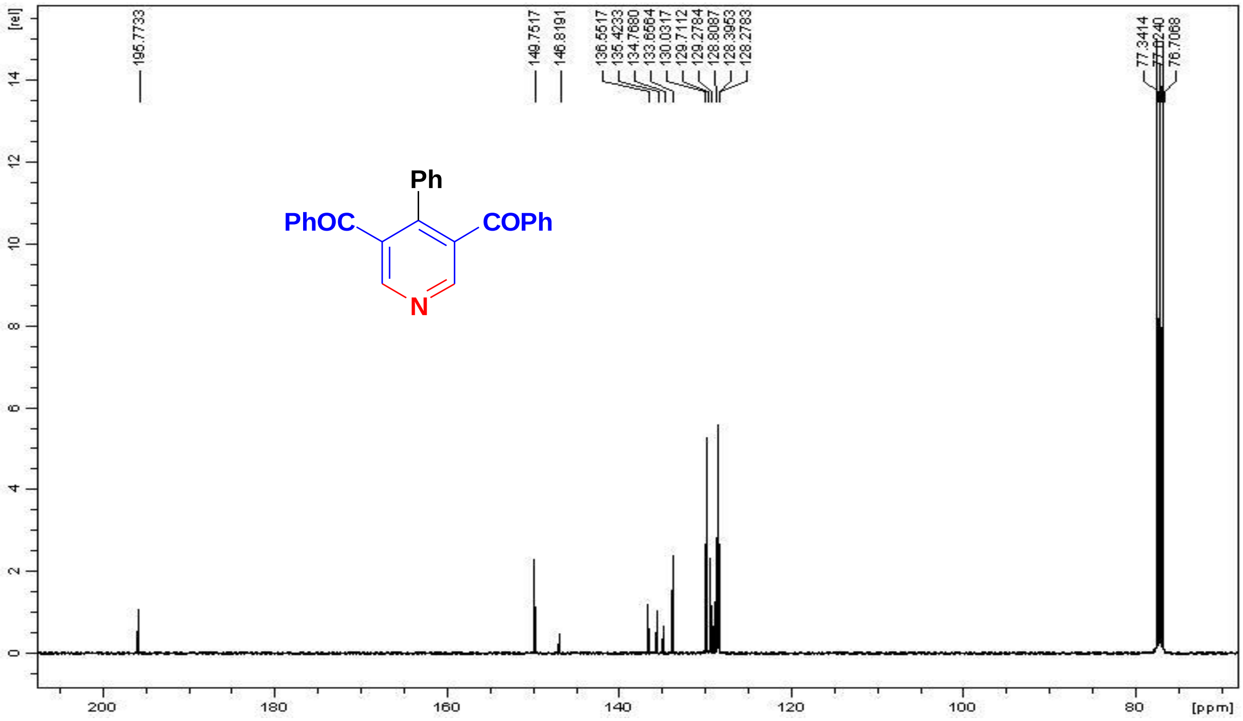

Compound **15a**

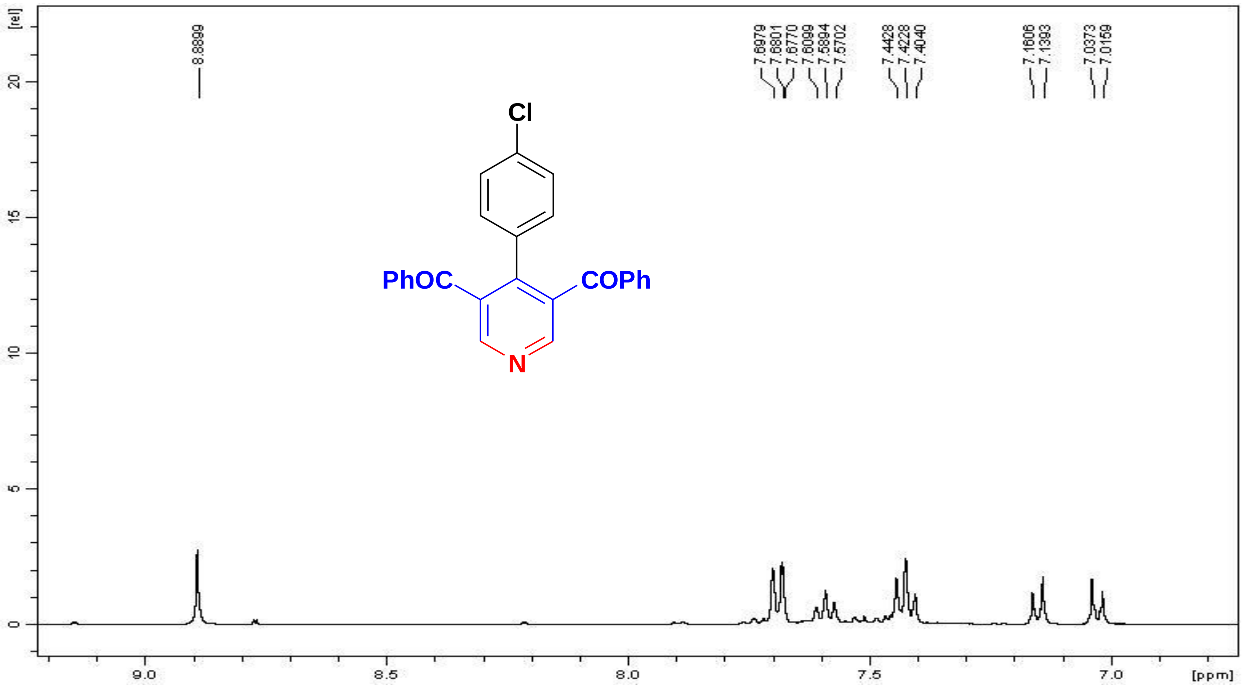

Compound **15b**

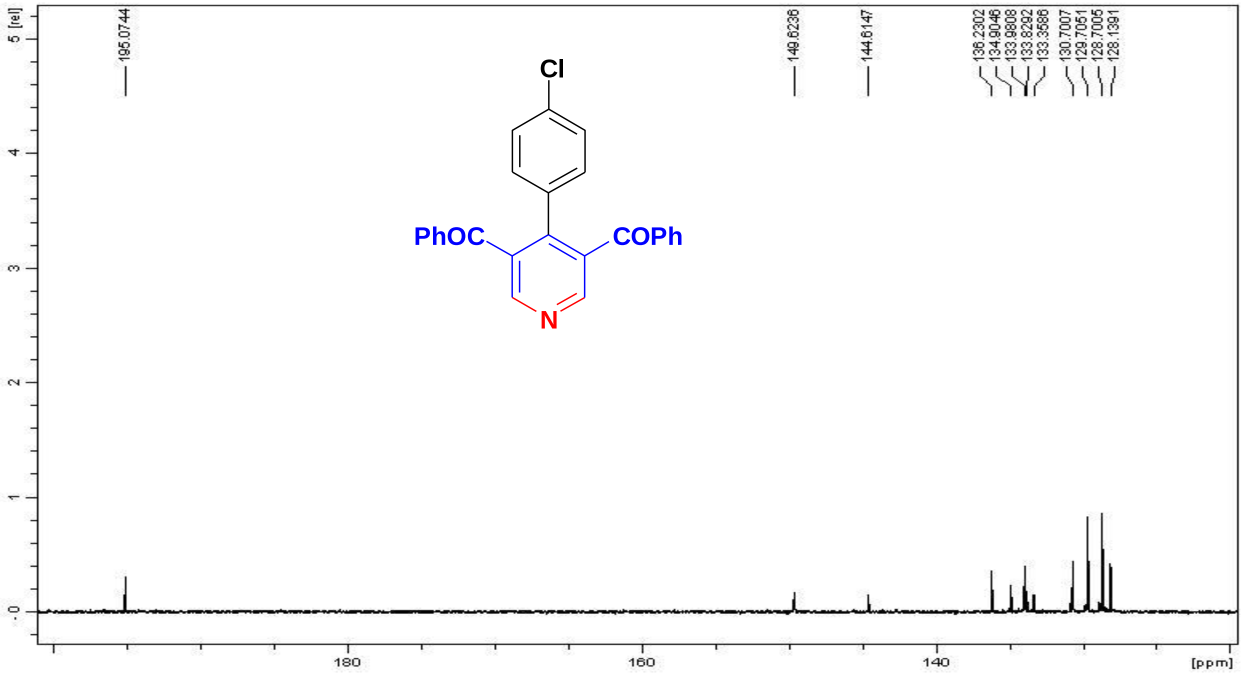

Compound **15b**

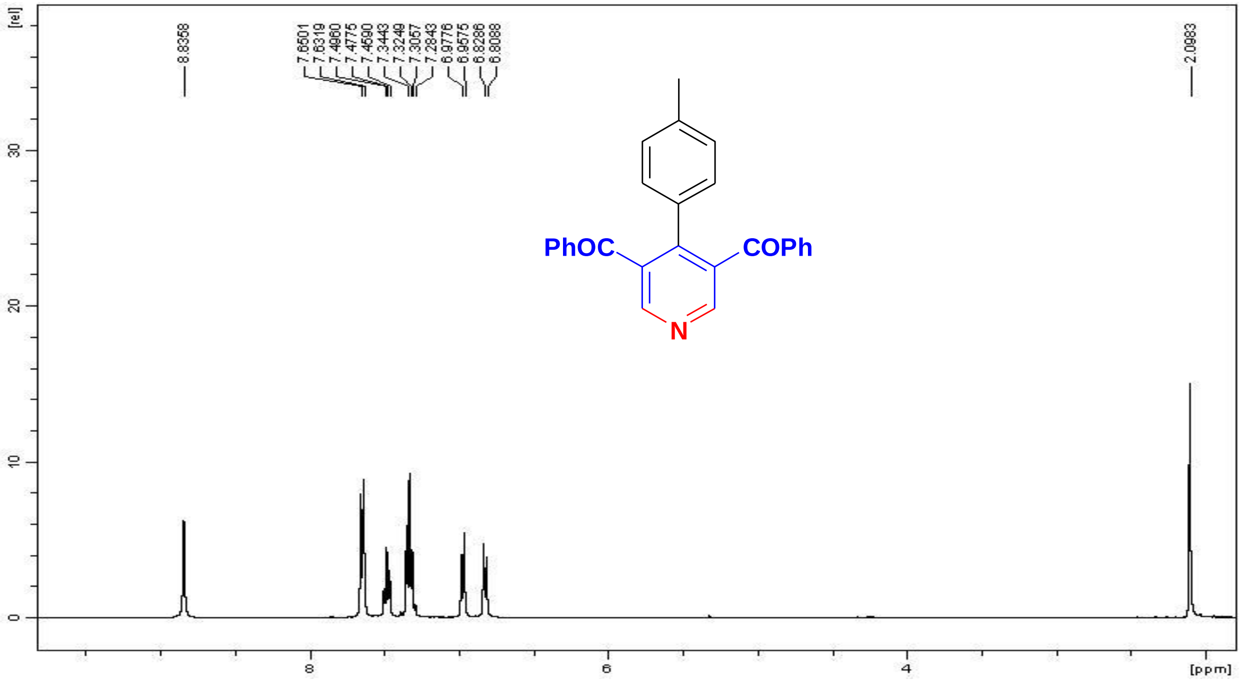

Compound **15c**

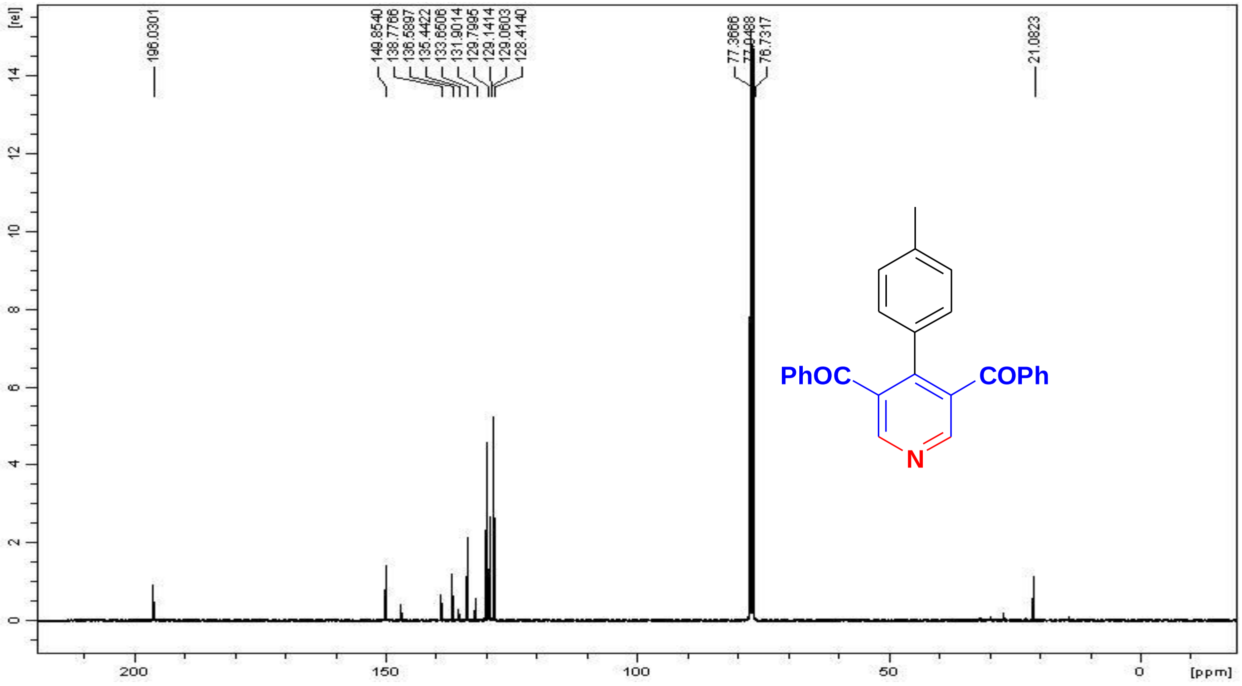

Compound **15c**

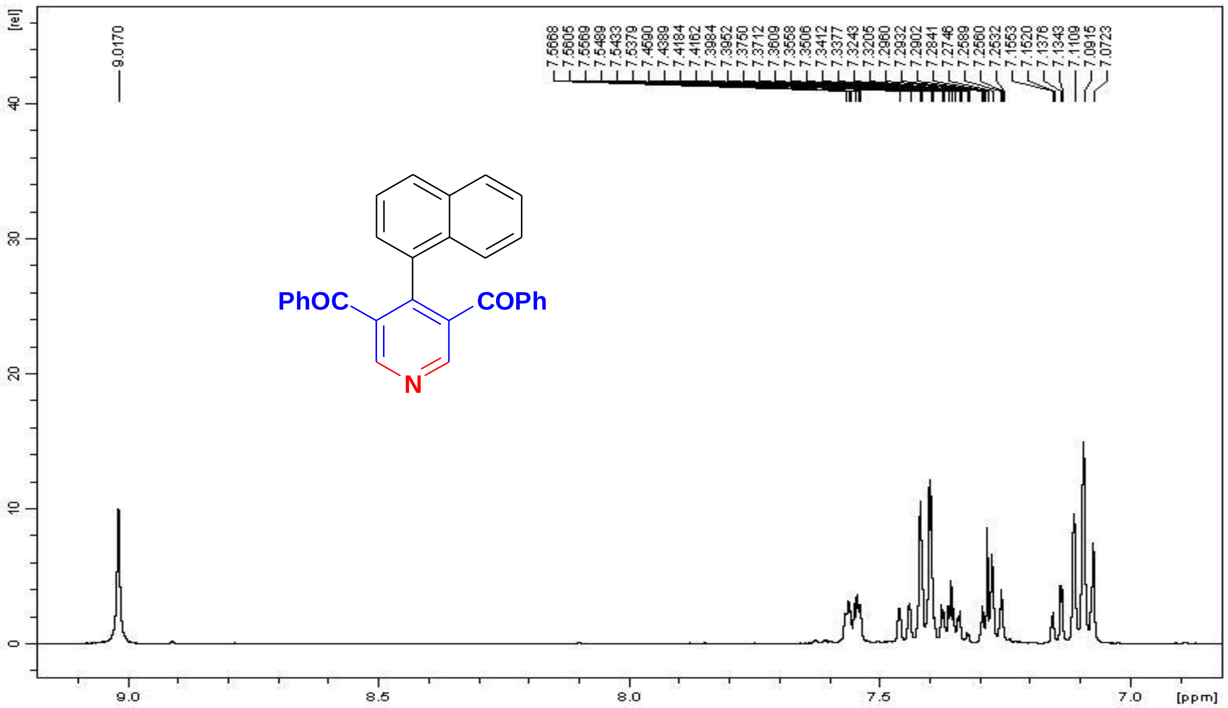

Compound **15d**.

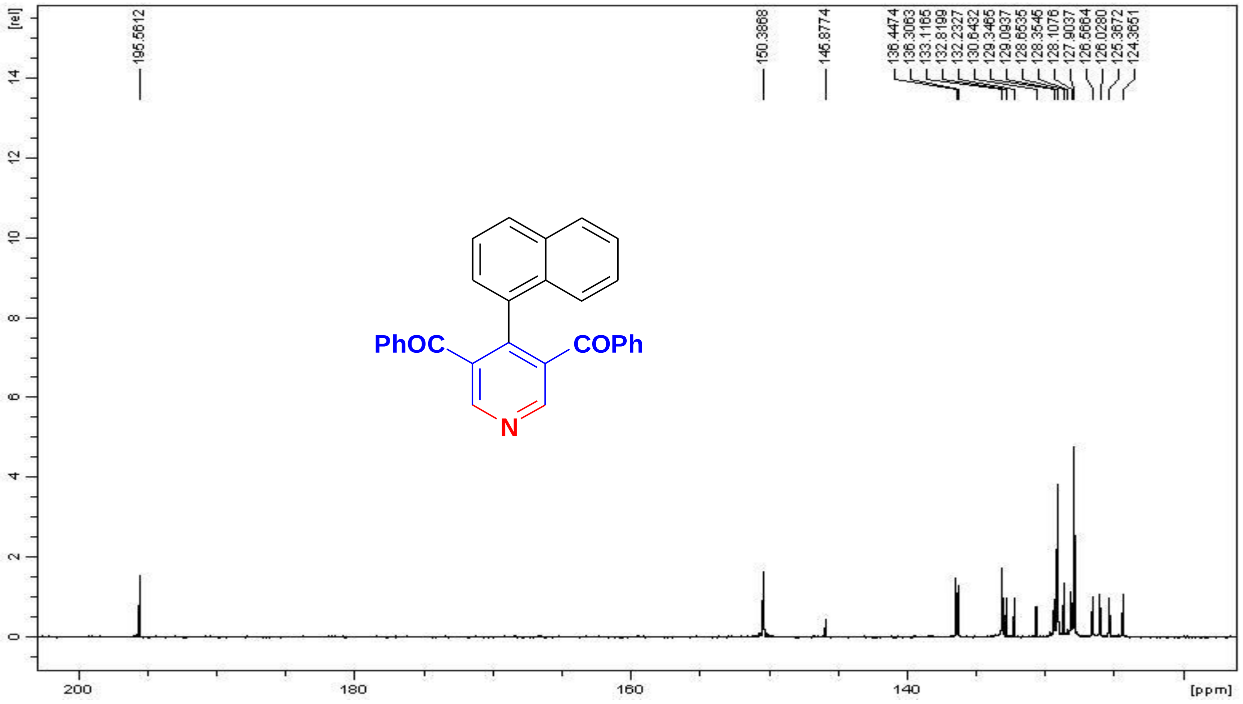

Compound **15d**.
